# Spliceosomal Intron Insertions in Genome Compacted Ray-Finned Fishes as Evident from Phylogeny of MC Receptors, Also Supported by a Few Other GPCRs

**DOI:** 10.1371/journal.pone.0022046

**Published:** 2011-08-05

**Authors:** Abhishek Kumar, Anita Bhandari, Rahul Sinha, Pankaj Goyal, Alessandro Grapputo

**Affiliations:** 1 Department of Biology, University of Padua, Padova, Italy; 2 Abteilung für Botanische Genetik und Molekularbiologie, Botanisches Institut und Botanischer Garten, Christian-Albrechts-Universität zu Kiel, Kiel, Germany; 3 International Graduate School (IHI) Zittau, Zittau, Germany; 4 Cold Spring Harbor Laboratory, Cold Spring Harbor, New York, United States of America; 5 Klinik und Poliklinik für Frauenheilkunde und Geburtshilfe, Universitätsklinikum, Der Ernst-Moritz-Arndt-Universität Greifswald, Greifswald, Germany; University of South Florida College of Medicine, United States of America

## Abstract

**Background:**

Insertions of spliceosomal introns are very rare events during evolution of vertebrates and the mechanisms governing creation of novel intron(s) remain obscure. Largely, gene structures of melanocortin (MC) receptors are characterized by intron-less architecture. However, recently a few exceptions have been reported in some fishes. This warrants a systematic survey of MC receptors for understanding intron insertion events during vertebrate evolution.

**Methodology/Principal Findings:**

We have compiled an extended list of MC receptors from different vertebrate genomes with variations in fishes. Notably, the closely linked MC2Rs and MC5Rs from a group of ray-finned fishes have three and one intron insertion(s), respectively, with conserved positions and intron phase. In both genes, one novel insertion was in the highly conserved DRY motif at the end of helix TM3. Further, the proto-splice site MAG↑R is maintained at intron insertion sites in these two genes. However, the orthologs of these receptors from zebrafish and tetrapods are intron-less, suggesting these introns are simultaneously created in selected fishes. Surprisingly, these novel introns are traceable only in four fish genomes. We found that these fish genomes are severely compacted after the separation from zebrafish. Furthermore, we also report novel intron insertions in P2Y receptors and in CHRM3. Finally, we report ultrasmall introns in MC2R genes from selected fishes.

**Conclusions/Significance:**

The current repository of MC receptors illustrates that fishes have no MC3R ortholog. MC2R, MC5R, P2Y receptors and CHRM3 have novel intron insertions only in ray-finned fishes that underwent genome compaction. These receptors share one intron at an identical position suggestive of being inserted contemporaneously. In addition to repetitive elements, genome compaction is now believed to be a new hallmark that promotes intron insertions, as it requires rapid DNA breakage and subsequent repair processes to gain back normal functionality.

## Introduction

Spliceosomal introns are associated with a well-defined splicing machinery in the nuclear genomes of all characterized eukaryotes, but the origin of these introns remains a puzzle [1]–[3]. To date, the most parsimonious explanation of their origin is that these introns are evolutionarily related to group II self-splicing introns [1], [4]. Insertion or deletions of spliceosomal introns are rare events in evolution. Intron insertions have been reported to be very rare in many metazoan lineages, including mammals and other vertebrates [5]–[7]. Limited information is available concerning mechanisms of intron gains [1], [3]. There are five models, which have been proposed for novel intron insertions such as intron transpositions [8], transposon insertions [9]–[16], tandem genomic duplications [9], intron transfers [17] and self-splicing type II introns [18]–[20]. Yet to date, definitive evolutionary processes governing novel intron acquisition in vertebrate genomes have largely remained elusive [1].

Fishes are the largest and the most diverse group of vertebrates that exhibit high level of biodiversity in their morphology, physiology, ecology and behaviour [21]–[23]. Such diversities are attributed to their hyper-variable genomic elements; there are several fishes whose genomes are severely compacted, for instance, hundreds of megabases in pufferfishes [24], [25], and highly expanded genomes, up to 130 gigabases for marbled lungfish, *Protopterus aethiopicus* [21]. These gigantic variations at the genomic level among fishes are very attractive as they serve as extremely useful models for the study of various evolutionary concepts such as tandem duplications, whole genome duplications and insertion/deletions of introns.

There are five different melanocortin receptors (MCR), melanocortin 1 receptor (MC1R), MC2R, MC3R, MC4R and MC5R, which have been reported from different tetrapod genomes. Taken together, they constitute the MC receptor protein family within the G-protein coupled receptor (GPCR) superfamily.The different MC receptor subtypes have very diverse physiological roles [26]–[28]. The MC1R is expressed mainly in the skin where it has a role in skin and hair/fur pigmentation in most mammals [29] and in feather pigmentation in chicken [30]. Further, MC1R also mediates the anti-inflammatory and immunomodulatory effects of the melanocyte stimulating hormone (MSH) peptides [28]. MC2R is exclusively found in the adrenal gland in mammals and mediates the effect of the adrenocorticotropic hormone (ACTH) on steroidoneogenesis [31]. The pharmacological profile of MC2R is different from that of the other MC receptors since it binds only to the ACTH and not the MSH peptides. MC3R and MC4R are also involved in regulating energy homeostasis and are important drug targets among GPCRs [32]. The activation of MC4R causes anorexia whereas inactivation of the MC4R leads to overeating and obesity [33]–[36]. The MC5R is expressed in wide array of tissues and has a role in exocrine gland secretion in mice [26], [37]. MC receptors are conserved from fish to mammals [38]. Various studies have contributed to the characterization of MC receptors from different fishes [38]–[41]. MC receptor genes have intron-less structures with a few exceptions noted in some fishes [38], [42]. Hence, MC receptors serve as a good system to study spliceosomal intron insertions.

In the current study, we assembled MC receptor orthologs/paralogs from vertebrates and we have demonstrated that fishes have a variable number of MC receptors with two in lampreys to six in zebrafish. Interestingly, the exon-intron structure analysis demonstrates dramatic changes in structures of MC2R and MC5R genes from a group of ray-finned fishes that underwent genome compaction. Upon extending these analyses to other GPCRs, we traced similar patterns of intron insertions in P2Y receptors and CHRM3 gene from these fishes. Furthermore, ultrasmall introns are found in the MC2R gene from selected fishes, indicating such introns exist at least in fishes.

## Results

### Characterization of the vertebrate repository of MC receptors

To understand the molecular evolution of any gene family or superfamily, defining the dataset is the critical initial step. In order to define our data set of MC receptors, we carried out similarity searches against different vertebrate genomes (see materials and method). The details of MC receptors are tabulated in **table S1**. The encoding protein sequences of these MCR genes are aligned as depicted in **figure S1**. There are five MC receptors namely MC1R–5R, which are present in mammals, birds, and other tetrapods. However, there are only four MC receptors– MC1R, MC2R, MC4R and MC5R in fishes such as *Takifugu rubripes*, *Tetraodon nigroviridis*, *Oryzias latipes*, and *Gasterosteus aculeatus*, with the exception of *Danio rerio* where there are six MC receptors with two copies of MC5Rs named as MC5aR and MC5bR and a copy of MC3R-like gene. When we traced for MC receptors in basal fishes, we detected three MC receptors, which are similar to MC1R (AAVX01456471.1, length- 854 bp), MC3R (AAVX01131452.1, length- 1728 bp) and MC5R (AAVX01069419.1, length- 4869 bp) from the elephant shark (*Callorhinchus milii*) genome. We further detected two MC receptors from draft assembly of lamprey, *Petromyzon marinus*. These were similar to MCA and MCB receptors, which have been described previously in another species of lamprey, *Lampetra fluviatilis* [43]. We did not detect MC receptor-like genes from non-vertebrate chordates; instead we found other closely associated GPCR homologs. Moreover, a single copy of MC receptor like gene is detected from *Branchistoma floridae* (JGI accession id: e_gw.70.133.1).

**Figure 1:**
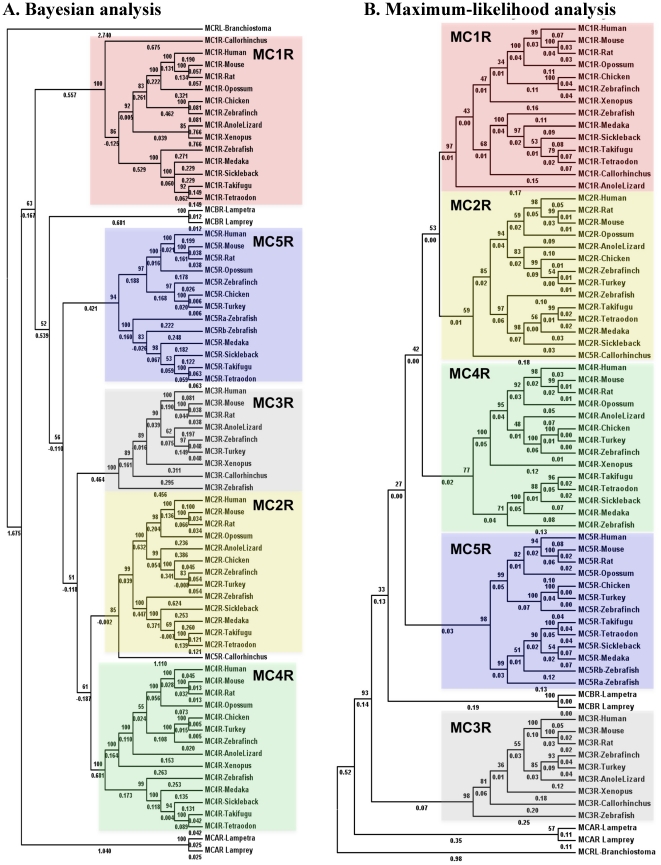
Evolutionary relationships of melanocortin receptors from selected vertebrates as presented by Bayesian analysis (A) and Maximum likelihood analysis (B). There are five major branches in these phylogenetic trees, dividing MC1R–5R into individual branches (distinct colors). The MC receptors from basal fishes are included in this phylogenetic tree such as MC A receptor (MCAR, ABB36647.1) and MC B receptor (MCBR, ABB36648.1) from lamprey *Lampetra fluviatilis* and corresponding genes from *Petromyzon marinus*, and MC receptors - MC1R (AAVX01456471.1) MC3R (AAVX01131452.1) and MC5R (AAVX01069419.1) from elephant shark *C. milii*. Outgroup in these phylogenetic trees is melanocortin like protein (MCRL) from *B. floridae* (JGI accession id - e_gw.70.133.1).

All MC receptor sequences have highly conserved DRY motif and a core domain of hepta-transmembranes, which share 70% or more similarity (marked in black background in **figure S1**). To understand the relationship among MC receptors, we reconstructed phylogenetic trees (**Figure 1**) based on the Bayesian and maximum likelihood methods. There are five main branches in these phylogenetic trees representing five MC receptor (MC1R–5R) genes that are found conserved from the teleost fishes to mammals. Two MC receptors (MCAR and MCBR) from lampreys branches out separately. The MCBR is found close to MC5R, as evident from this phylogenetic tree and further confirmed by BLAST searches of MCBR from lampreys against non-redundant (NR) database, where the best hits picked up are MC5Rs from teleost fishes.

#### MC2R and MC5R locus is conserved from teleost fishes to mammals

**Figure 2:**
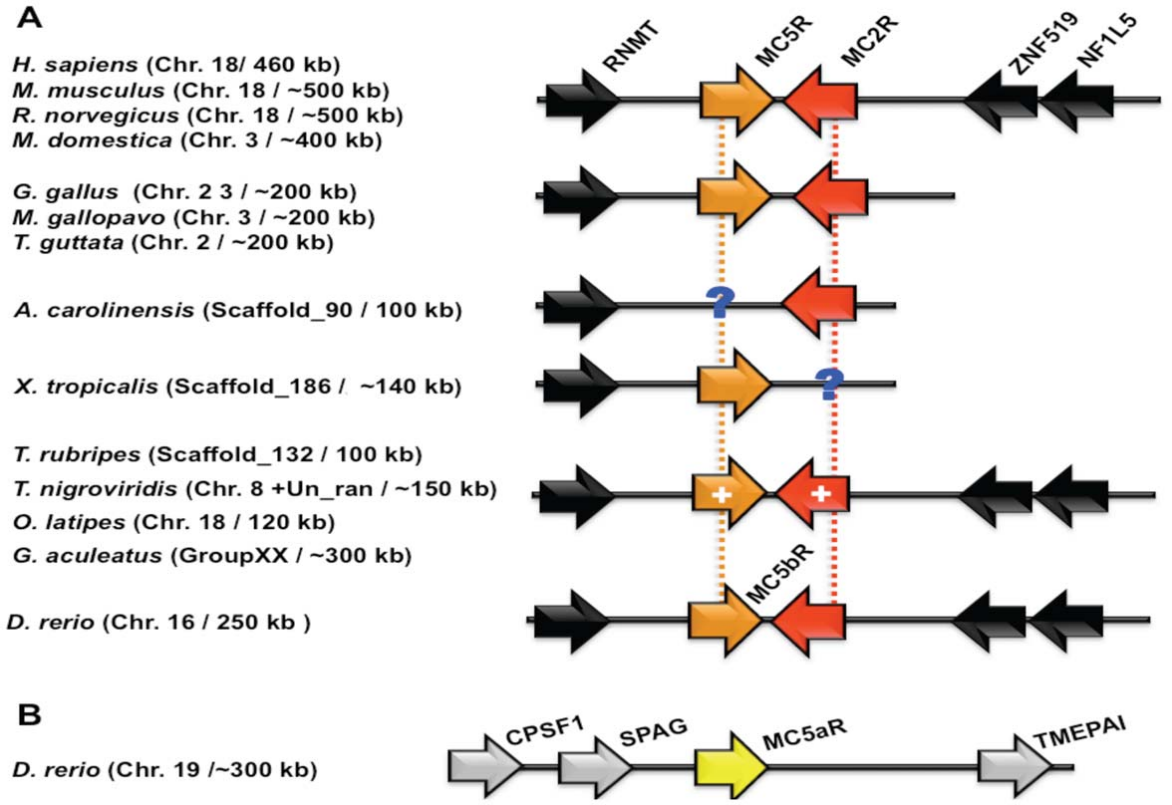
Orthology assessment of closely linked MC2-MC5Rs with help of different genome browsers. **A.** Orthologs of MC2-MC5Rs from fish to human are conserved flanking by a marker gene RNMT on one side and a dyad of ZNF519-NF1L5 on the other side. In a group of four ray-finned fishes, introns are inserted (marked by +), where as in tetrapods and in *D. rerio* (MC5bR), there are no intron insertions. **B.** A paralog of MC5R is found in independent locus in *D. rerio* named as MC5a receptor (yellow color) with different sets of marker genes (marked in grey).

To gain further insight into the evolutionary processes that generated the MC receptors, we compared the genomic locations and the gene orders surrounding the MC receptors. In the human genome, MC2R and MC5R genes are found to be closely linked on chromosome 18, flanked by the RNA (guanine-7-) methyltransferase (RNMT) gene on one side and a dyad of markers, zink finger protein 519 (ZNF519) and neurofibromin 1-like 5 (NF1L5), on the other side (**Figure 2A**). This genomic fragment is maintained in several mammalian genomes such as mouse (chromosome 18–∼500 kb fragment), rat (chromosome 18–500 kb fragment) and opossum (chromosome 3–400 kb fragment). This genomic organization is also conserved in birds, for example chicken (chromosome 2), turkey (chromosome 3) and zebra finch (chromosome 2) in a 200 kb region with the RNMT gene flanking on one side, however, we did not detect the conserved markers on the other sides. In the only available reptile genome, anole lizard, this fragment is present on scaffold_90 comprising of only RNMT and MC2R genes, whereas, the amphibian, *Xenopus. tropicalis* genome contains this conserved chromosomal fragment on scaffold_186 and consists of only RNMT and MC5R genes. The missing MC5R and MC2R from anole lizard and the frog genomes, respectively are due to the fragmental nature of current genome assembly of these two organisms. When we searched this genomic fragment in different fish genomes, we found that the closely linked MC5R and MC2R genes are conserved on a similar fragment in *T. rubripes* (scaffold_132, 100 kb), *O. latipes* (chromosome 16, 120 kb) and *G. aculeatus* (group XX, ∼300 kb). In the *T. nigroviridis* genome, MC2R and MC5R are located on two different chromosomes, chromosome Un_random and chromosome 8, respectively. Since this fragment is present in the closely related pufferfish *T. rubripes* on a single locus, it is highly unlikely that *T. nigroviridis* can have MC2R and MC5R on independent loci suggesting that it is a genomic assembly error rather than an evolutionary event. This architecture is maintained in *D. rerio* (chromosome 16, 250 kb) with closely associated MC5bR and MC2R, whereas, MC5aR is localized on chromosome 19 flanked by dyad of marker genes, sperm-associated antigen 1 (SPAG1) and cleavage and polyadenylation specific factor 1 (CPSF1) on one side and vertebrate transmembrane prostate androgen-induced (TMEPAI) protein on the other side (**Figure 2B**), corroborating that *Danio* MC5bR is an ortholog of MC5R, whereas *Danio* MC5aR is a paralog. It is clear from **Figure 2** that closely linked MC2R and MC5R genes have orthologs in different fishes, however, novel introns in these two genes are found only in a group of ray-finned fishes and not in tetrapods or *D. rerio* (details in later section).

#### Chromosomal localization of MC1R

We extended the micro-synteny analysis to other MC receptors in order to gain an insight into their origin. MC1R is located on chromosome 16 in the human genome flanked by Fanconi anemia, complementation group A (FANCA) and transcription factor 25 (TCF25) on one side, and differentially expressed in FDCP 8 (DEF8) and dysbindin (dystrobrevin binding protein 1) domain containing 1 (DBNDD1) on the other side in a 350 kb region (**Figure S2**). This genomic architecture is maintained in several mammalian genomes such as mouse (chromosome 8), rat (chromosome 19) and opossum (chromosome 1). This synteny is also conserved in birds such as chicken (chromosome 19) and zebra finch (chromosome 11) with an additional marker gene, programmed dead cell 5 (PDCD5). However, we could not detect MC1R gene in turkey genome probably due to the fragmental nature of the present genomic sequence. In the anole lizard genome, MC1R is present on small scaffold_2608, where it is not possible to analyse the surrounding marker genes. However, *X. tropicalis* genome contains this conserved chromosomal fragment on scaffold_303. Upon searching this genomic fragment in different fish genomes, we found that it is conserved in *T. rubripes* (scaffold_14), *T. nigroviridis* (chromosome 5), *O. latipes* (chromosome 3), *G. aculeatus* (group II) and *D. rerio* (chromosome 18).

#### Status of MC3R orthologs in vertebrates

In the human genome, MC3R gene is surrounded by Cerebellin 4 Precursor (CBLN4) gene on one side and a tetrad of markers, AURKA, CSTF1, CASS4 and GCNT7, on the other side in a region of about 550 kb (**Figure S3A**). This chromosomal fragment is present in several mammalian genomes such as mouse (chromosome 2: 450 kb fragment), rat (chromosome 3: 470 kb fragment) and opossum (chromosome 1: 700 kb fragment). Surprisingly, even though this fragment is present in chicken genome on chromosome 20 in a region of ∼190 kb. However, the MC3R gene is missing at this locus. To understand this loss of MC3R gene, we searched this genomic fragment in two different bird genomes such as zebra finch and turkey using genome browsers. We found that this syntenic architecture is present in these two birds along with the MC3R gene on chromosome 20 in zebra finch and chromosome 22 in turkey, respectively with fragment size of ∼180 kb in both cases. We detected the MC3R protein sequence from chicken upon similarity searches against GenBank (Accession id: BAA32555.1), indicating the presence of a MC3R homolog in chicken genome, though this homolog is not yet located on the chromosomal map due to sequencing error in current genome assembly. In the anole lizard genome, MC3R is present on small scaffold_168, where it is not possible to analyse the flanking marker genes. However, *X. tropicalis* genome contains this conserved chromosomal fragment on scaffold_148 in a 280 kb region. On searching this genomic fragment in different fish genomes, we did not find MC3R to be conserved in *T. rubripes*, *T. nigroviridis*, *O. latipes*, *G. aculeatus* and *D. rerio*. However, the previously reported MC3R of *D. rerio* is localized on a separate location characterized by different sets of markers flanking both ends of this MC receptor. This fragment contains the MC receptor homolog flanked by a triad of PTP1B, CEBPB and SRXN1 on one side and a dyad of SLC13A3 and GRID2 on the other side (**Figure S3B**). We investigated the existence of this loci in other ray-finned fishes and we found that this loci is maintained with same sets of markers in other ray finned fishes such as *T. rubripes* (scaffold_226), *T. nigroviridis* (chromosome 9), *O. latipes* (chromofsome 7), and *G. aculeatus* (group XII). However, this loci has another GPCR, thyrotropin-releasing hormone receptor 3 (TRHR3) as corroborated by homology searches (Ensembl id are listed in **table S2**). There are two duplicates of TRHR3 in *T. rubripes* and we named it as TRHR3a and TRHR3b, respectively. Conservation of this loci in different fishes, which is drastically different than the tetrapod MC3R loci, corroborates that it might be a consequence of fish-specific genome duplication event [44], During evolution it was further coupled by loss and translocation events that represent a complex evolutionary origin today. Hence, the MC3R and TRHR3 genes have a complex evolutionary history in fishes that appears to have been influenced by a combination of loss, duplication and/or translocation.

#### MC4R loci are conserved from fishes to mammals

In the human genome, MC4R is localized on chromosome 18 flanked by dyad of markers, CDH6 and RNASEN on one side, and CDH20 on the other side. This genomic organization is conserved from fishes to mammals (**Figure S4**). Other mammals, which are known to carry this fragment, include mouse (chromosome 18), rat (chromosome 18) and opossum (chromosome 3). This fragment is maintained in bird genomes such as chicken (chromosome 2), turkey (chromosome 3) and zebra finch (chromosome 2). The anole lizard genome carries this fragment in scaffold_168; however, we could not localize the CDH20 marker in the current draft version of this genome. MC4R is also found at similar locus in *X. tropicalis* (scaffold_95). Five analysed fish genomes contain this architecture, *T. rubripes* (scaffold_96), *T. nigroviridis* (chromosome 5), *O. latipes* (chromosome 20), *G. aculeatus* (group XXI) and *D. rerio* (chromosome 2).

Unfortunately, to date, the genome assemblies of elephant shark (cartilaginous vertebrates) and lamprey (jawless vertebrates) remain too fragmentary to get more insights into the micro-synteny architecture of the MC receptor clusters (data not shown). However, when we summarize the orthology assessment of MC receptors, it is noteworthy to mention that there are only four MC receptor orthologs conserved in different fishes. Although *D. rerio* contains six MC receptors, it lacks one-to-one or the true ortholog of tetrapod MC3R. As described above, the previously believed MC3R from zebrafish is not a genuine ortholog of MC3R. Two additional MC receptors from *D. rerio* are paralogs of MC3R and MC5R, respectively. Hence, it is expected that these paralogs may have different physiological function in *D. rerio* supported by the hypotheses about fates of duplicated genes.

Moreover, it is clear that from teleost fishes to human, MC receptors are maintained as such without much expansion or reduction over a period of 450 MYA. Overall, MC receptors are vertebrate-specific genes, since no homologs have been detected with requisite similarities to confirm its existence in invertebrate genomes, as we described above. A recent review also supported this view [45].

### Gene structures of MC receptors

**Figure 3:**
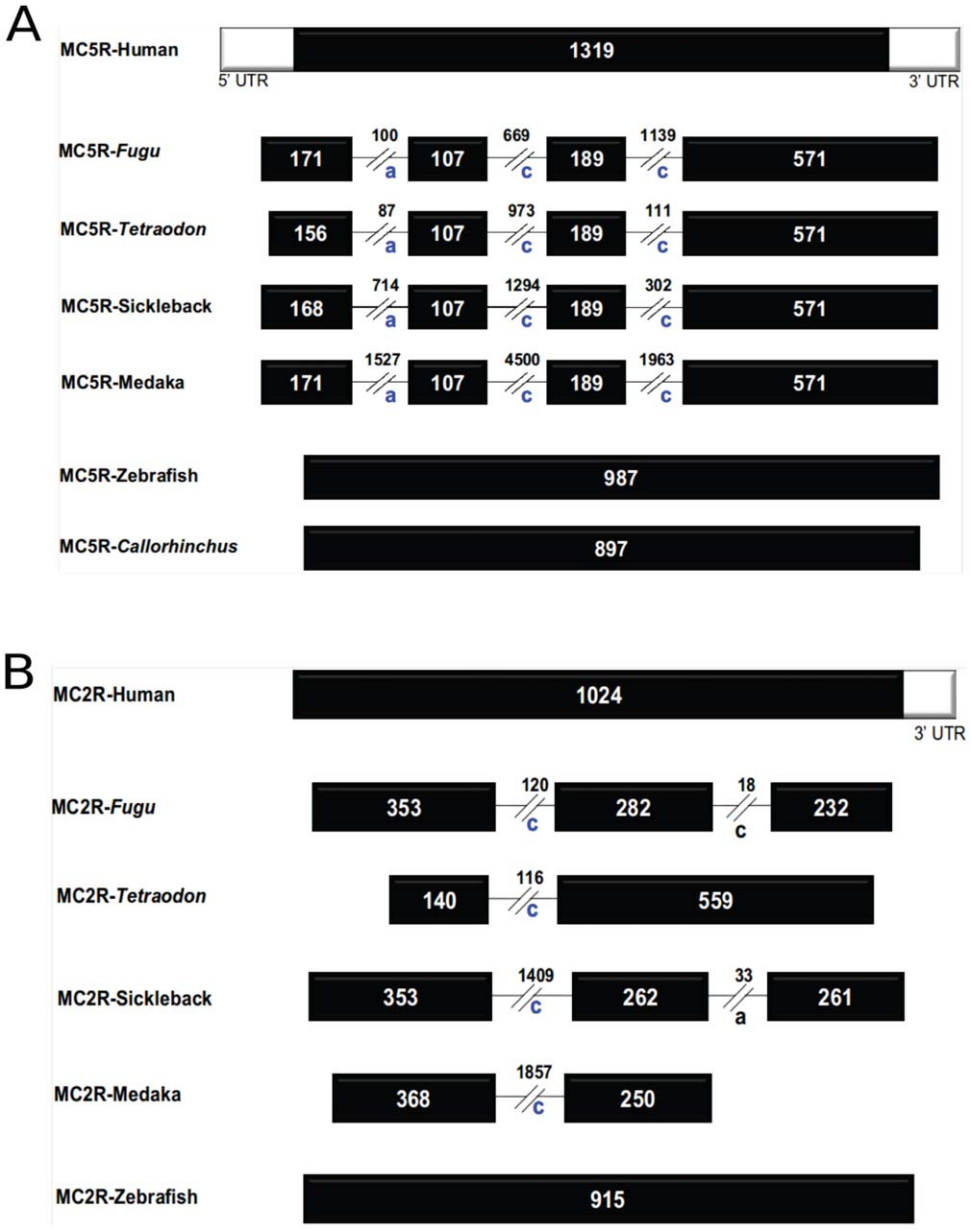
Exon-intron architecture of selected MC receptors. **A. Gene structure of MC5 receptors.** **B. Gene structure of MC2 receptors.** Black bars represent exons in coding regions and white bars represent exons in UTR regions. Introns are represented by black lines. Size of introns and exons are given in values in respective introns and exons. Intron phasing is marked by a–c, indicating intron phasing according to their location after the first, second, or third base of the corresponding codon, respectively. The intron phasing of conserved intron is marked by blue color while black color for non-conserved introns as found in case of MC2Rs from *Takifugu* and stickleback.

To elaborate on the mechanism(s) of intron insertions, gene structures of MC receptors from different vertebrates are illustrated in **Figure 3**. Primarily, the intron-less gene structure is characteristics of MC receptors from tetrapods, *D. rerio*, elephant shark and lampreys, with MC5R and MC2R from four ray-finned fishes being the only exceptions.

In order to evaluate the novel introns on MC5R, we mapped these introns onto the amino acid alignment of MC5Rs (**Figure S5**). There are three introns inserted at positions 41a, 77c and 140c (full-length human MC5R amino acid numbering with suffix a–c for intron phasing) in MC5Rs of four fishes, *T. rubripes*, *T. nigroviridis*, *O. latipes*, and *G. aculeatus*. However, these novel introns were not traced in MC5R like genes from *D. rerio*, elephant shark (*C. milii*) and tetrapods (**Figure 3A**). These novel introns at positions 41a, 77c and 140c are all found in the conserved domain of MC receptors with over 70% identical regions such as TM1, TM2 and in the highly conserved DRY motif at the end *of the TM1*, respectively.

**Figure 4:**
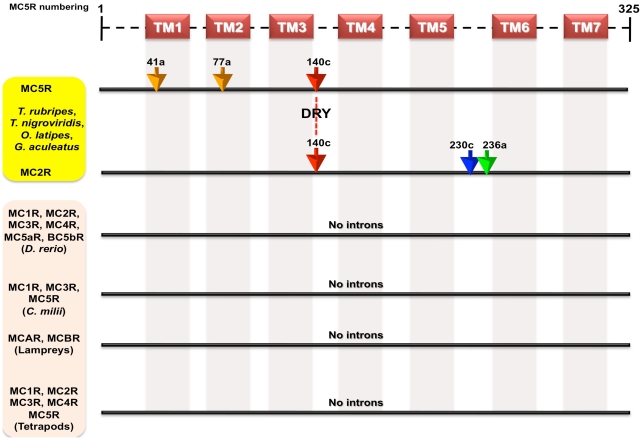
Schematic illustration of gene organizations of different MC receptors from different vertebrates depicting novel intron insertions in MC5R and MC2R from selected ray finned fishes. There are three intron inserted at positions 41a, 77c and 140c (human MC5R amino acid numbering with suffix a–c for intron phasing) in MC5Rs of four fishes - *Takifugu*, *Tetraodon*, stickleback and medaka. There is one intron inserted at position 140c in MC2Rs of same four fishes. The intron at position 140c is found in the middle of highly conserved DRY of both MC5R and MC2R. Takifugu and stickleback has one intron inserted in their MC2R at intron positions 230c (blue arrow) and 236a (green arrow), respectively. These introns are not found in any other MC receptors from zebrafish, lampreys, elephant shark and tetrapods. Transmembrane regions are marked as TM1–7 as predicted by TMHMM2.0 [106].

Similarly to understand intron insertions in MC2Rs, we mapped the novel introns to the MC2R protein sequences (**Figure S6**). We found only one intron inserted at the position 140c (full-length human MC5R amino acid numbering) in MC2Rs of the four fishes, *T. rubripes*, *T. nigroviridis*, *O. latipes*, and *G. aculeatus* (**Figure 3B**). This intron like the MC5R intron at position 140c is also found in conserved domain of MC receptors with over 70% identical regions and in the middle of highly conserved DRY motif at the end of the TM3. MC2R also has an additional species-specific intron each at positions 230c in *T. rubripes* and 236a in *G. aculeatus*, respectively. These novel introns are not found in MC2R orthologs from *D. rerio* and tetrapods. As mentioned above, we found that one intron at position 140c is faithfully maintained in the two closely linked MC5R and MC2R genes in four different fishes. These two genes from zebrafish, which share a common ancestor with the four ray-finned fishes remained intronless on a similar locus, suggesting the notion of novel intron insertions in parallel in these genes. These findings are summarized in **Figure 4**, where we have mapped the intron positions on protein sequences (as per human MC5R amino acid numbering). The existence of introns in closely linked MC2R and MC5R have been reported previously in pufferfishes [38], [46] and in *O. latipes* and *G. aculeatus* [47].

#### Characteristics of MC2R and MC5R introns

**Table 1** summarises some properties of the novel introns identified from MC5R and MC2R from a specific lineage of ray-finned fishes. Their sizes vary from 87 to 4500 bp (**file S1**). These novel introns are bordered by canonical GT-AG splice signals, the overall GC content extends from 37 to 55.9%. Two out of the three conserved introns interrupt the open reading frame between codons (phase c), and remaining one after the first nucleotide (phase a), respectively. We did not detect any significant similarities to known complex repetitive elements in these novel introns from pufferfishes. However, we found some repetitive elements in *O. latipes*, and *G. aculeatus*. This explains the increase in intron size in these two fishes as compared to the shorter introns from the pufferfishes. All of the novel introns of MC5 receptors interrupt a highly conserved domain of a typical GPCR with introns at positions 41a, 77c and 140c, respectively, without any insertions or deletions (**Figure S1**). The GC content of novel introns from these MC5R genes is ranging from 37% to 55.9%. In contrast, MC2R has only one conserved novel intron at position 140c with GC content 37.6 to 44%. MC2R from *T. rubripes* contains an additional intron located at position 230c that in the loop region between helices TM5 and TM6, which is a predominant region of insertion and deletions (**Figure S1**). Similarly, MC2R from *G. aculeatus* also contains an additional intron at position 236a again in the loop region between helices TM5 and TM6, albeit closer to the beginning of TM6 region in this case. Both these non-conserved novel introns from *T. rubripes* and *G. aculeatus* are exceptionally smaller in size, 18 and 33 bp, and GC content 66.7% and 93.9%, respectively.

**Table 1:** Features of novel introns inserted in MC2R and MC5R genes from a group of ray-finned fishes.

Fishes	Gene	Intron#	Intron	Flanking	Percentage	Repetitive elements€		
			size	sequences*, @	GC content	Type (Class) [Diection, d/c$]	From	To
***T. rubripes***	MC5R	41a	100	**A** ***G*** **AG↑GTCT**	37	N.A.	N.A.	N.A.
***T. nigroviridis***	MC5R	41a	87	**A** ***G*** **AG↑GTAA**	55.2	N.A.	N.A.	N.A.
***G. aculeatus***	MC5R	41a	714	**C** ***G*** **AG↑GTAT**	34.5	N.A.	N.A.	N.A.
***O. latipes***	MC5R	41a	1527	**G** ***G*** **AG↑GTCT**	47.5	SINE_FR1D (NonLTR/SINE) [d]	1157	1243
***T. rubripes***	MC5R	77c	669	**GCAG↑GTGA**	41.3	N.A.	N.A.	N.A.
***T. nigroviridis***	MC5R	77c	973	**GCAG↑GTGA**	41.1	(TG)_n_ (Simple repeat) [d]	301	360
***G. aculeatus***	MC5R	77c	1294	**GCAG↑GTAA**	41.4	UnaL2 (NonLTR/L2) [d]	2858	2929
***O. latipes***	MC5R	77c	4500	**GCAG↑GTGA**	38.5	Crack-1_HM (NonLTR/Crack) [d]	31	108
						DNA-8-23_DR (DNA) [c]	457	552
						MRE1_OL (Interspersed repeat) [d]	942	997
						FUROUSHA2 (DNA/hAT) [d]	1416	1530
						DNA-8-14_DR (DNA) [c]	1675	1778
						FUROUSHA2 (DNA/hAT) [d]	2056	2121
						FUROUSHA2 (DNA/hAT) [d]	2187	2299
						ERV46_MD_I (ERV/ERV1) [d]	2526	2578
						Polinton-1_DR (DNA/Polinton) [d]	2871	2945
						CR1-57_HM (NonLTR/CR1) [c]	3331	3393
						piggyBAC-N1_OL (DNA/piggyBac) [d]	4080	4285
***T. rubripes***	MC5R	140c	1139	**ACAG↑GTAA**	40	N.A.	N.A.	N.A.
***T. nigroviridis***	MC5R	140c	111	**ACAG↑GTGA**	55.9	N.A.	N.A.	N.A.
***G. aculeatus***	MC5R	140c	302	**ACAG↑GTAA**	38.7	DNA-8-14_DR (DNA) [c]	176	236
***O. latipes***	MC5R	140c	1963	**ACAG↑GTAA**	40.2	SINE_FR2 (NonLTR/SINE) [d]	434	795
						hAT-2_NV (DNA/hAT) [d]	1836	1883
***T. rubripes***	MC2R	140c	120	**ACAG↑GTTT**	40	N.A.	N.A.	N.A.
***T. nigroviridis***	MC2R	140c	116	**ACAG↑GTAT**	44	N.A.	N.A.	N.A.
***G. aculeatus***	MC2R	140c	1409	**AC** ***C*** **G↑GTAC**	37.6	UnaL2 (NonLTR/L2) [c]	554	599
						Chapaev-12_HM (DNA/Chapaev) [c]	634	683
						hAT-14_HM (DNA/hAT) [c]	914	1005
***O. latipes***	MC2R	140c	1857	**AT** ***C*** **G↑GTAC**	43.5	RTE-1_TD (NonLTR/RTE) [d]	220	297
						DNA-TA-5_DR (DNA) [c]	668	789
						RTE-2_AFC (NonLTR/RTE) [d]	834	1201
						RTE-2_AFC (NonLTR/RTE) [d]	1213	1698
***T. rubripes***	MC2R	230c%	18	**G** ***GC*** **G↑GTGG**	66.7	N.A.	N.A.	N.A.
***G. aculeatus***	MC2R	236a	33	**T** ***GGT*** **↑GGCC**	93.9	(CGG)_n_ (Simple repeat) [d]	4	33

*The intron insertion points are indicated by **↑** sign.

@ Bases deviating from the proto-splice site sequence (MAG↑R), where M = A/C and R = A/G [48], are marked in italics.

# Numbering of human MC5 receptor is followed as standard MC receptor.

% 225c-FRU corresponding numbering according to MC2R from *T. rubripes*.

€ Repetitive elements were predicted using Repeat Masker package, version 3.2.6 and RepBase Censor (http://www.girinst.org/censor/index.php) at default settings.

$Direction - d - direct or c – complementary.

Intron insertions have been proposed to occur at preferred locations (C/AAG↑) referred to as proto-splice sites [48]. We examined sequences enclosing the insertion points of novel introns in these two MC receptor genes (**Table 1**). The proto-splice site is mostly maintained, however, exceptions from this is also evident in the cases of the intron at position 41a (GAG↑GT) of MC5R in all four fishes and the intron at position 140c (CG↑GT) of MC5R in the case of *G. aculeatus* and *O. latipes*. Similar mutations are also found in non-conserved novel introns of MC2Rs from *T. rubripes* and *G. aculeatus*. Therefore, with a few mutations at some of the proto-splice sites, the intron insertion sites are largely maintained.

### Presence of other novel GPCR introns in genome compacted fishes

**Figure 5:**
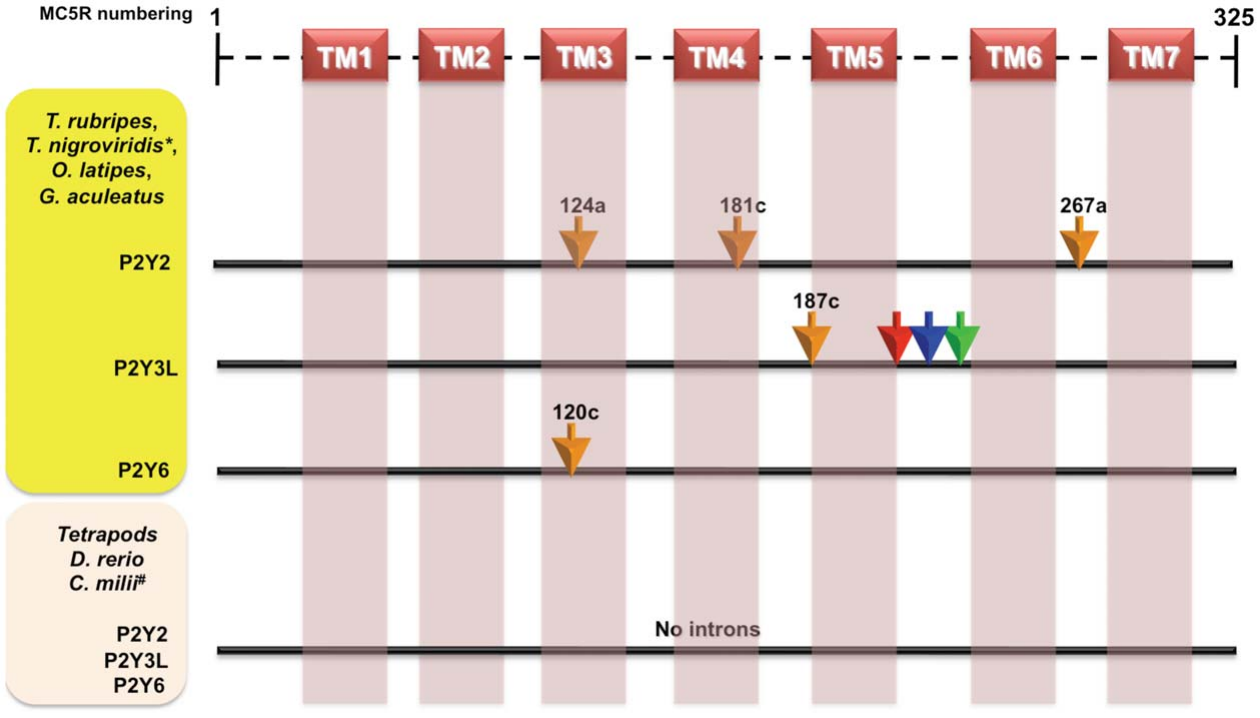
Gene structures of different P2Y receptors from different vertebrates depicting novel intron insertions in P2Y2, P2Y3L and P2Y6 receptors from selected ray finned fishes. There are three intron inserted at positions 124a, 181c and 267a (human MC5R amino acid numbering with suffix a–c for intron phasing) in P2Y2 receptors of four ray-finned fishes (*Takifugu*, *Tetraodon*, stickleback and medaka). There is one intron inserted at position 187c in P2Y3L of same fishes and there is one each species-specific intron S212a (stickleback; red arrow), M (medaka; blue) and T (Takifugu; green). The intron at position 120c is found in P2Y6 receptors of these fishes. These introns are not found in any other P2Y receptors from zebrafish, elephant shark and tetrapods. Transmembrane regions are marked as TM1–7 as predicted by TMHMM2.0 [106]. * indicates that novel P2Y3L is missing in Tetraodon genome. # indicates that P2Y6 receptor was not found in current assembly of elephant shark (*C. milli*).

To find more cases as described above for the MC2Rs and the MC5Rs, we scanned various other GPCRs. Indeed, we found introns, which have been inserted in similar fashion as above in some other GPCRs. Purinergic P2Y receptors, such as P2Y2, P3Y3-like (P2Y3L) and P2Y6 receptors also have novel introns inserted in the genome compacted ray-finned fishes **(** **Figure 5**). More specifically, in all these fishes, we found that there are three intron insertions in the respective P2Y2 receptors, at the positions 124a (in TM3), 181c (in TM4) and 267a (in the middle of loop joining TM6 and TM7); one novel intron insertion in the P2Y3L receptors, at position 187c (Figure S7); one species-specific intron insertion in each of the species, restricted only to the loop between TM5 and TM6 of the P2Y3L receptors (**Figure S8**); and a single intron insertion at position 120c in the P2Y6 receptors (**Figure S9**). These introns are not found in the P2Y receptors from zebrafish, elephant shark and tetrapods.

We also found that there are two introns insertions at positions 67a (close to the TM1), 166c (at the end of the TM3; DRY intron or 140c by human MC5R numbering) in CHRM3 receptor of four fishes - *Takifugu*, *Tetraodon*, stickleback and medaka (blue background), but not in CHRM3 genes from zebrafish, elephant shark and tetrapods (Figure S10). Additionally, CHRM3 from each of the four fishes have species-specific intron insertions (red background in **Figure S10**), confined only in the large loop between TM5 and TM6.

We scanned for the proto-splice site is conservation in all these novel introns in P2Y and CHRM3 receptors as done for MC receptors. These receptors with their respective proto-splice sites are listed in **table S3**.

Taken together, our data indicate that these are novel intron insertions that occurred after separation of genome compacted fishes (or superorder *Acanthopterygii*) from *D. rerio* (or superorder *Ostariophysi*) just like the intron insertions described above for the MC2Rs and the MC5Rs, probably via a similar mechanism.

### DRY introns repertoire of GPCR from vertebrates with non-compacted and compacted genomes

To gain insight into the repertoire of DRY introns from non-compacted and compacted vertebrate genomes, we carried out further analyses of previously reported other GPCRs that possess DRY introns in vertebrates [49]. We surveyed these GPCRs from (a) vertebrates with non-compacted genomes including human, mouse, rat and zebrafish, and (b) vertebrates with compacted genomes including *Takifugu*, *Tetraodon*, medaka and stickleback. We have summarized the GPCRs, each with their respective accession id, the codon usage of DRY motif, and the variant of DRY motif present in a particular GPCR, in **Table S4**. Among these GPCRs, we found that there are three dopamine receptors, D2–D4, which share DRY introns in many vertebrate species and have undergone species-specific gene duplications. Dopamine receptor D2 has two copies in *D. rerio*, whereas dopamine receptor D4 has two copies each in *Takifugu* and medaka, and four copies in *Tetraodon*. Similarly, two endothelin receptors (type A and B) have DRY introns shared from fishes to mammals with evidences of gene duplication in fishes. We also identified a DRY intron in GPR161 gene that has two copies in *D. rerio*. In contrast, GPR176 has single copy in all of the species with the exception of medaka, which has no GPR176 gene. Two galanin receptors, GALR2 and GALR3, also share DRY intron, but only the GALR2 intron is conserved in all of the species whereas the DRY intron of GALR3 is limited only to mammals. The DRY intron in Gastrin-releasing peptide receptor (GRPR) gene is shared in all of the considered species with evidence of gene duplication in fish genomes. Melanin-concentrating hormone receptor 2 (MCHR2) gene has DRY intron in human and fishes, whereas rodents have no MCHR2 gene. Neuromedin B receptor (NMBR) gene has DRY intron in all of the species. Two Neuropeptide FF receptors (NFFR1–2) also possess this intron in all of the analysed genomes and NFFR2 has gene duplications in fishes. Two prokineticin receptors (PROKR1–2) share this intron in all of the genomes with a single copy of each gene. Prolactin releasing hormone receptor (PRLHR/GPR10) gene has DRY intron only in the fish genomes but not in the mammalian genomes that were taken into consideration. Two serotonin (5-hydroxytryptamine) receptors, HTR4 and HTR7 contain DRY intron in all of the analyzed vertebrates with two copies of HTR7 genes in *D. rerio*. Three tachykinin receptors (TACR1–3) have a DRY intron that is conserved from fishes to mammals with duplication of TACR2 and TACR3 in different fish genomes. We found that thyrotropin releasing hormone receptor 2 (TRHR2) has a DRY intron in the rodents and all of the fishes with two copies of this gene in *D. rerio*; however, the TRHR2 gene is missing from the human genome. Until now, the TRHR2 gene carrying DRY intron has not been reported in fishes [49]. Bombesin-like receptor 3 (BRS3) gene has a DRY intron in mammals; however, BRS3 gene was not found in any of the analyzed fish genomes.

**Figure 6:**
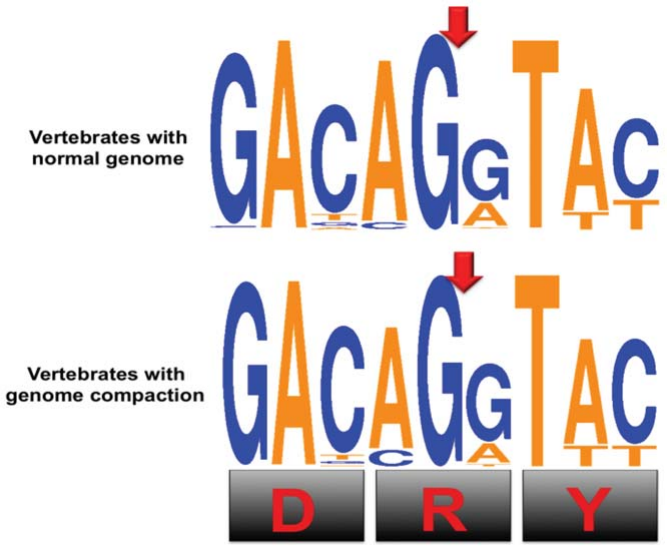
Comparison of codon usage for DRY motifs of GPCRs from vertebrates with non-compacted genome versus compacted genome. Red arrow indicates the site of intron insertion. Orange and blue colors represent nucleotides A/T and C/G, respectively. The codon usage of DRY motifs were represented using sequence logo generated by weblogo 3.0 [112].

In summary, we conclude that out of 24 common GPCRs that possess DRY introns 23 are maintained from fishes to mammals with a few exceptions. We observed that duplications in these GPCR genes occur only in the fish genomes. Furthermore, we compared the variation in the codon usage of the DRY intron between non-compacted and compacted genomes of vertebrates and we noted that there was no much variation in the DRY codon usage (**Figure 6**).

## Discussion

We carried out a comprehensive analysis of MC receptors using genomic fragments, gene structures and protein sequences to unravel orthologs and paralogs across a wide range of evolutionarily distant vertebrates. Contrary to the tetrapods, we found that fishes have a variable number of MC receptor genes. There are four MC receptors, which are conserved from fishes to human, MC1R, MC2R, MC4R and MC5R. The syntenic organization is strongly conserved for the majority of MC receptors and previously, this remarkable synteny conservation between chicken and mammals has been experimentally validated [50]. Selz *et al.* (2007) have also reported that fishes have variable repertoire of these receptors and they further observed that the expression profile of MC1R was different in divergent fish lineages [47]. The MC3R of *D. rerio* does not share genomic organization with MC3Rs of tetrapods, inferring that it might not be an ortholog of tetrapod MC3R. Moreover, zebrafish MC3R branches out earlier than elephant shark MC3R in a highly accurate Bayesian phylogenetic tree (**Figure 1A**) providing further hint that zebrafish MC3R is of a different origin. Notwithstanding, the sequence similarities that closely related GPCRs possess in general, the notion of their common origin is overhauled by such differences. This is further supported by the absence of γ-MSH, the main ligand for MC3R in ray-finned fishes [46], [47]. There is no MC3R like gene in other fish genomes that are available. We detected three and two MC receptors from elephant shark and lamprey, respectively. With no clear orthologs from invertebrate species, it is evident that these MC receptors originated at the beginning of vertebrate evolution at around 450/500 MY ago. The presence of MC receptors at different loci across the vertebrate spectrum, from teleost fishes to humans, suggests that they evolved by a process of duplication that happened very early during vertebrate evolution. Other than *D. rerio*, none of the teleost fishes contain fish-specific loci of MC receptors.

*D. rerio* possesses two copies of MC5 receptors. Previously, it was reported that these two *D. rerio* MC5R sequences are not a very recent duplicates and the duplication event that created these two receptors took place in the teleost lineage after the divergence of tetrapods, which is usually dated to 300 MY ago as estimated by molecular clock calculations [51]. These two MC5Rs from *D. rerio* have similar pharmacological and expression profile in different tissues [52] supporting the developed degeneration complementation model for the fates of duplicated genes [53].

Further, we found that MC2Rs and MC5Rs of selected group of fishes have unusual gene structures when compared to the orthologous receptors from tetrapods (**Figures 3** **–** **4**). Normally, MC receptors do not possess introns, but MC5R and MC2R from *T. rubripes*, *T. nigroviridis*, *O. latipes*, and *G. aculeatus* contain one and three introns, respectively, at identical positions. However, we did not detect introns in MC2R and MC5R receptors from *D. rerio*, where all MC receptors were intron-less as found in tetrapod MC receptor gene structures.

In different studies, unusual exon-intron boundaries of MC5R and MC2R have been reported previously in pufferfishes [38], [46] and in *O. latipes* and *G. aculeatus* [47] for different purposes. In this study, we have systematically studied the properties of these introns for possible explanations for novel intron insertions. Intron insertions are considered to be rare genomic changes (RGC) [54] across a wide range of metazoan lineages such as mammals [5] and puffer fishes [6]. Our data suggest that the MC5R genes of the four ray-finned fishes represent a clear proof of novel intron insertions, which is absent in tetrapods, *D. rerio*, and in elephant shark; all of which possess the typical single intron-less gene structure. There are three introns (at positions 41a, 77c and 140c) in orthologs of MC5R from a group of selected ray-finned fishes that are found after the split of the *D. rerio* lineage (or superorder *Ostariophysi*) from the superorder *Acanthopterygii* (other four ray-finned fishes belong to this superorder). Similarly, unusual gene structures of MC2R from the same group of ray-finned fishes were also found after diversification of *D. rerio* from these fishes. Some introns are ultrasmall in size as found in case of novel insertion at position 230c and 236a in MC2R from *T. rubripes*, and *G. aculeatus*, respectively. Such small introns are rare; however, it cannot be completely excluded as the *T. rubripes* genome has some smaller introns [24]. Indeed, some recent reports suggest for the existence of such introns in different mesozoa [55], [56]. Though splicing is not strictly dependent on the size of the introns, it may be critical in controlling splicing efficiency [56].

MC5R and MC2R from the four fishes share an intron at position 140c (**Figure 4**) indicating that this intron is inserted at the same time in parallel within these genes. It was debated whether this intron is an ancient intron [38], [49] or a recently inserted intron [42]. It is a notorious task to unravel a common origin of 140c (or DRY) intron, since it is found in phylogenetically non-clusterable rhodopsin GPCRs [42]. To resolve this issue, fourteen ancestral receptor groups (ARG) were determined by the presence of the human receptor in all vertebrates plus least in two invertebrate species (*C. intestinalis*, *C. savignyi*, *C. elegans*) and Fridmanis *et al.* (2007) suggested that the DRY intron may have been inserted several times independently in different GPCRs [57]. All novel introns in MC receptors are examples of recent insertion as no MC receptor was detected in invertebrate species. In addition, sharks and zebrafish do not have these introns, corroborating that it is an exclusive feature of the four genome-compacted fishes in the superorder *Acanthopterygii*. Consequently, our data support that these introns are recently inserted in these selected GPCRs after separation of *D. rerio* from other ray-finned fishes. Fridmanis et al. (2007) also briefly suggested that the DRY intron of these two MC receptors are recent rather than ancient [57]. We found similar pattern of insertions of novel introns in some other GPCRs, which further support the late creation of introns in selected ray-finned fish genomes.

We scanned updated genomes of vertebrates for differentiating DRY intron repertoire of non-compacted versus compacted genomes. We upgraded the existing catalogue of the DRY introns shared among different vertebrates [49]. We found that the codon usage of the DRY motif had not much variation between non-compressed versus compressed genomes of vertebrates (**Figure 6**).

We surveyed DRY motifs with/without an intron in different GPCRs and some examples are given in tables S2, S3, S4. We found that DRY introns are not only found in the conserved DRY motifs but also elsewhere in different GPCRs where DRY-like motifs were conserved, again with similar intron phasing (phase c). For example, MCHR2 from zebrafish has this intron in DRY motif and also has a similar intron in HRY motif (about 104 amino acids downstream from the typical DRY motif). Previously, Fridmanis et al. (2007) have also reported such cases where they found that both conserved DRY and DRY-like motifs possess introns with conserved proto-splice sites [57]. Based on the variations present in all of the characterized DRY motifs, we observed that [DEHLIS] [R] [YFSN] includes all the variations allowed in a typical DRY motif, where at the first position five others substitutions are observed with roughly equal frequencies, the second position has highly conserved arginine (R) and the third position has predominantly a tyrosine (Y) with some less frequent permitted variations. These multiple reoccurrences of DRY introns can be explained by the codon usage of the first two amino acids of DRY motifs (**Table 2**). The arginine (R) has two types of codon usages such as AGU, where U = A/G, and CGN, where N = any nucleotide, with the former being more frequent at the exon junctions of the respective DRY introns. The third base of the first amino acid codon and the first two bases of the second amino acid codon of the DRY motif are jointly instrumental in the formation of the proto-splice site (MAG, where M = A/C) with a few exceptions. This view was supported previously by others with limited data [42].

**Table 2:** Codon usage for DRY motifs with possible amino acid substitutions.

Position 1		Position 2	Position 3	
		Arg (R)		
Asp (D)	G-A-[*C*T]	*A*-*G*-[AG]C-*G*-[ACGT]	T-A-[CT]	Tyr (Y)
Glu (E)	G-A-[AG]			
His (H)	C-A-[T*C*]		T-T-[CT]	Phy (F)
Leu (L)	C-T-[*AC*GT]			
Ile (I)	A-C-[*AC*T]		A-A-[CT]	Asn (N)
Ser (S)	A-G-[*C*T]T-C-[*AC*GT]			
			A-G-[CT]C-G-[ACGT]	Ser (S)

Italics marked nucleotides are standard nucleotide substitution allowed at proto-splice site.

**Figure 7:**
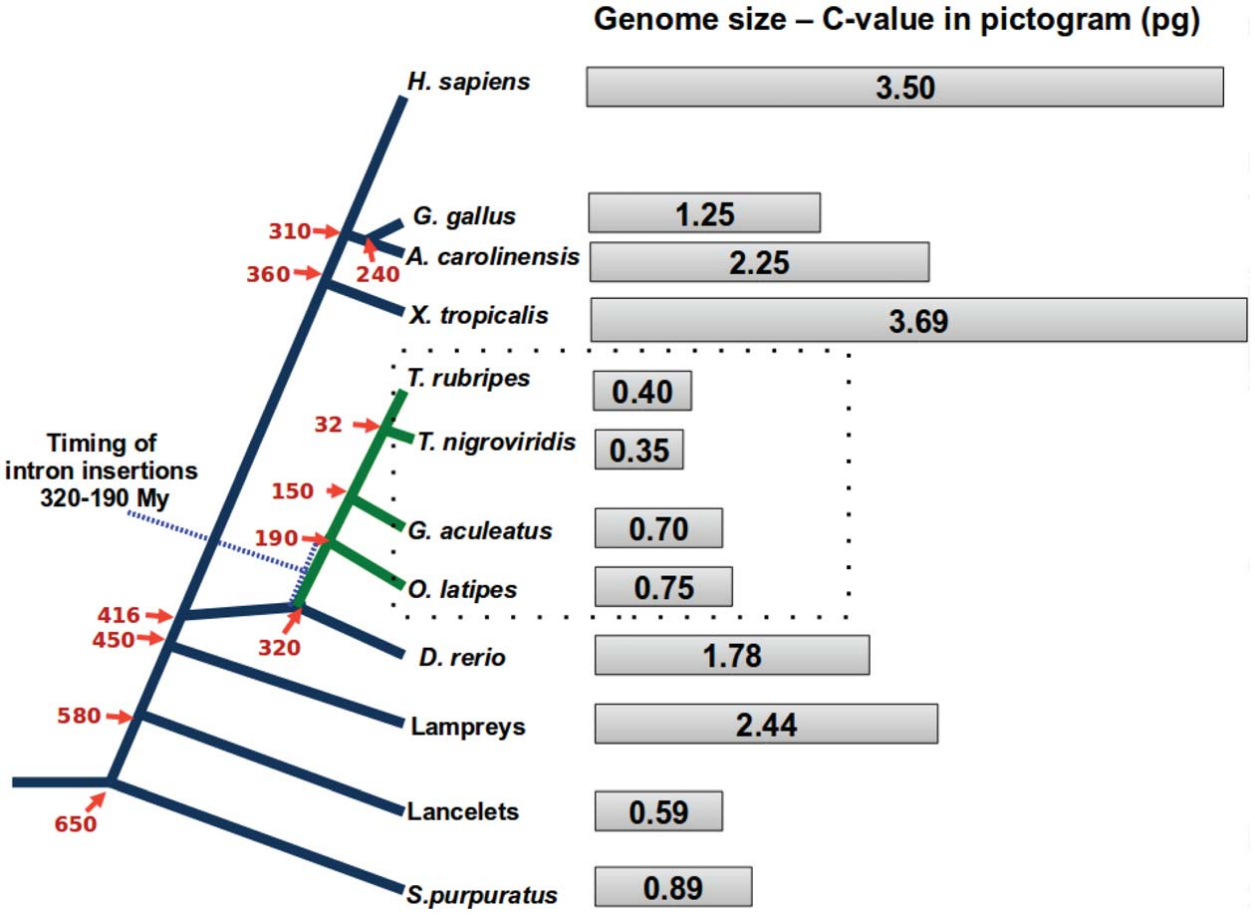
Phylogenetic history of vertebrates depicting intron insertions in some GPCR genes in selected ray-finned fishes that underwent genome compactness. The estimated divergence times in MYA (marked with red arrows) are used from ref. [61]. The time interval of intron insertions in ray-finned fishes is 320–190 MYA (marked by tilted blue T). Genome sizes of selected evolutionary important organisms were primarily taken from animal genome size database [71]. Genome size of anole lizard is calculated using values 1 pg = 978 Mb and estimated genomic DNA content of 2.2 Gb as reported in the proposal of anole lizard genome sequencing project [113].

Previously reported cases of novel introns in vertebrates are also associated with the diversification of these fishes [7], [58]–[60]. To deduce the timing of such intron insertions in some of the GPCRs from the selected group of ray-finned fishes, we reconstructed an evolutionary history of selected organisms using time scale as provided by Chris Ponting [61]. Our data supports the idea that these insertions appear to have happened at about 320 to 190 MYA (**Figure 7**), before or during the emergence of the superorder *Acanthopterygii*. The emergence of introns can be explained by several mechanisms such as involvement of transposons [1], [62]. The presence of remarkably diverse retrotransposable elements with hints of recent activities and it is one of the prominent features of fish genomes [23], [24], [63]. We found repetitive elements in novel introns from stickleback/medaka lineage but not in tetraodontidae; therefore, it is highly likely that there is a loss of these repeat elements from newly acquired introns preferentially in pufferfishes.

The origin of these newly acquired introns remains open for investigations. Largely, due to the fact that during our searches using different homology search suites, these novel introns do not have significant homology either to the flanking sequences within the locus or anywhere else in overall sequenced parts of these fish genomes. Every genome sequencing project faces some problems in sequencing process, which result in unavailability of small proportion of genomic sequences. Therefore, fractions of genomic sequences from these selected fish genomes, if missing, are likely to remain unsequenced. Thus, a remote possibility exists that the novel introns are derived from some unsequenced portion of these fish genomes. However, this possibility is highly unlikely as these hypothetical unsequenced portions escaped detection in multiple fish genomes. The probability that all the four genomes have similar sequencing errors is extremely low, unless all of them carry some sequence features that make sequencing difficult in these regions. Similarly, the source of introns is unknown for a documented case of strain specific novel intron creation in rab4 gene from *Daphnia pulex* genome [64].

There are different types of processes that may be accountable for intron insertions with/without events responsible for the primordial emergence of spliceosomal introns. Proto-splice site is the hotspot for these intron insertions [65]–[69] as other insertions may not be compatible with life in general. The minimal requirement for proper spliceosomal performance is the presence of both authentic cis splice signals and core trans factor, The function of spliceosomes does not depend on how introns have evolved, which could be either by the insertion of intron sequences, probably created by expansion of short simple repeats or more complex repetitive elements [58], [70], or by intronisation of exonic sequences due to point mutations [62]. Genome compaction in many actinopterygians after the fish-specific whole genome duplication [24], [71]–[73] is considered to have promoted intron insertions in these fishes [7]. DNA double-stranded breaks (DSBs) and recombination, which involve repair and recombination processes are essential components of genome compactness. Furthermore, intron insertions are now considered to be resultant of error-based repair of DBSs that is predominantly mediated by non-homologous end joining (NHEJ) as recently reviewed [74], [75]. We report that missing repeats in introns from pufferfishes are most likely due to a higher degree of genome compactness in the tetraodontidae lineage when compared to the medaka/stickleback lineage (**Figure 7**).

Gene level novelties are created either by whole genome duplication or by segmental duplications, which may conceivably lead to the gain of novel introns, as an unaffected gene copy remains within the genome. The appearance of novel introns in the MC5R and MC2R cannot be directly coupled to gene or genome duplication, as none of these introns are found in MC receptors from *D. rerio*, which diverged at a time period closer to the fish-specific genome duplication event [76] than the other ray-finned fishes selected in this study. Whether intron insertions occur preferentially in multi-gene families or take place at random is still controversial [77], [78].

A recent study of multiple intron gains in serine protease inhibitor superfamily in the lineages of selected ray-finned fishes [7] illustrates similar results where the same four fishes have intron gains in selected serpin genes. Vertebrate serpins are classified by the presence of conserved intron positions, which are well-documented and are used in classification of vertebrate serpins into six groups V1–V6 [7], [79]. In contrast, the majority of MC receptors are primarily described by intron-less gene structures. However, it is interesting that genes with novel intron from both these superfamilies encode for single domain protein with peptide length of 300–400 amino acids with additional N-/C-terminal extensions. Upon comparisons of novel introns from both these superfamilies, we found that reported novel introns from serpins are considerably smaller than those of MC receptors. Further, there are no complex repeats detected in novel introns of serpins whereas novel introns from medaka and stickleback lineages possess complex repeats. The nucleotides that are flanking the novel introns from these MC receptors form a typical proto-splice site [48] with deviations at the 5′end, contrasting the results from novel introns of serpins. However, we found that intron phasing of majority of novel intron insertions in both MC receptors and serpins is same (phase c or phase 2). Previous large scale analyses of DRY introns indicate that repeated insertions at the same position and with phase c has occurred in different lineages of animals for these introns [49], [57]. After combining these data with ours for novel intron insertions, it appears that intron phasing c is selected during intron insertions, probably due to extra flexibility provided by the wobble at the third base of codons. It is clear that intron insertions have occurred in genomes of ray-finned fishes which underwent genome downsizing and these introns are inserted during period at about 320–190 MYA. It is now well established in two independent multi-gene superfamilies such as GPCR and serpins [7].

In summary, a group of ray-finned fishes exhibits multiple intron insertions in selected GPCRs and serpins [7] at ∼320–190 MYA, which are not present in any other vertebrate. Compaction in genomic contents of these fishes may have played a crucial role in these intron acquisitions. Fishes exhibit a high diversity after separation from last common ancestor of tetrapods/fishes lineage and these diversities can be explained by rapid change in DNA contents by processes such as whole genome duplication and genome compaction. Losses/gains in gene contents, introns, and intergenic regions are crucial to these events. Hence, genome compaction may be responsible for novel intron insertions at least in ray-finned fishes. Though one may argue against such possibility given our small sample size of four fishes, novel intron insertions are rare genomic events [54] and they are extremely rare during vertebrate evolution [6], probably because most such events would lead to deleterious effects.

### Conclusions

We have compiled a comprehensive catalogue of MC receptors from evolutionary distant vertebrates in this study. With a complete repository of MC receptors at hand, we carried out a study of introns in these genes. We found that introns are inserted in closely linked MC5R and MC2R from a selected set of actinopterygian fishes (*T. rubripes*, *T. nigroviridis*, *O. latipes*, and *G. aculeatus*) whose genomic content is reduced. Such insertion events are absent in genomes of zebrafish, lamprey, elephant shark and other tetrapods. Both these receptors share one intron inserted at common site in conserved DRY motif. We demonstrated that P2Y receptors and CHRM3 also had novel intron gains, suggesting that this mechanism is not restricted only to the MC receptors. Overall, we found that all novel intron insertions are found only in fish genomes, which had undergone reduction in genomic contents close to ∼320–190 MYA.

## Materials and Methods

### Retrieving sequences of the MC receptors from the draft/nearly completed genomes

We extracted the genomic DNA/cDNA/protein sequences from different eukaryotes via BLAST suite [80]–[82] searches using either MC1R or MC5R as a query sequence from the different genomes such as human [83], mouse [84], and rat [85] from National Centre for Biotechnology Information (NCBI) [86], *T. rubripes* [24], *X. tropicalis* [87] and *B. floridae* [88], *Nematostella vectensis* [89], *Helobdella robusta*, and *Lottia gigantean* from the Department of Energy's Joint Genome Institute (JGI; http://genome.jgi-psf.org/), *G. gallus* [90], *T. guttata* [91], *M. gallopavo* [92], *A. carolinensis*, *T. nigroviridis* [25], *O. latipes* [93], *G. aculeatus*, *D. rerio* and *M. domestica* [94] genomes from Ensembl [95], [96], elephant shark *C. milii* genome *from* the Elephant Shark Genome Project Web site (Eshark 1.4× assembly, website: http://esharkgenome.imcb.a-star.edu.sg/) and *S. purpuratus* genome from the Human Genome Sequencing Center (HGSC), Baylor College of Medicine (http://www.hgsc.bcm.tmc.edu/). All genomes used in this study are listed in **table S5**.

### Gene structure prediction and mapping introns positions

To ensure correct gene structures of all putative GPCR genes, we predicted gene structures using GENSCAN [97], [98] and predictions were repeated using GENOMESCAN [97], [98], GENEWISE [99] and FGENESH/FGENESH+ [31]. Intron-exon structures were determined with the aid of GENEWISE [99] and/or PROT_MAP module of the Softberry software suite (website: www.softberry.org). We used human MC5R as the standard sequence for projection of intron position and corresponding numbering of human MC5R is followed by suffixes a–c, indicating intron phasing according to their location after the first, second, or third base of the corresponding codon, respectively as described previously [7], [79]. If due to sequencing errors, non-human numbering was used; it is stated in that specific case. We created the pairwise alignment of mature human MC5R protein and specific GPCR proteins from different eukaryotes using MUSCLE alignment software [100], [101] and we marked intron positions, semi-automatically with manual inspection.

### Surveying repetitive elements in novel introns

We traced the repetitive elements in novel introns of MC5R and MC2R using Repeat Masker package version 3.2.6 (http://www.repeatmasker.org) and RepBase Censor (http://www.girinst.org/censor/index.php) [102] using default settings.

### Detection of syntenic conservation

To detect the orthologs or paralogs of the MC receptors, We analyzed micro-synteny across different genomes using NCBI mapviewer [103] and the following genome browsers: ENSEMBL [95], [96], JGI, *Tetraodon* at the Genoscope and UCSC [104].

### Protein sequence analyses

We generated protein alignments of different GPCRs with MUSCLE alignment program [100], [101] with default setting. We edited and visualized these alignments for different sequence characteristics using GENEDOC [105]. We predicted transmembrane domains of putative MC receptors using the Transmembrane Hidden Markov Model (TMHMM) server version 2 [106].

### Phylogenetic analyses

We used MUSCLE alignment program [100], [101] to align the amino acid sequences of MC receptors for the purpose of phylogenetic analyses. We constructed a phylogenetic tree by the Bayesian approach (5 runs, 100, 000 generations, 25% burn-in-period, JTT matrix-based model [107]) in the MrBayes program [108], [109] under the best selection model (SYM) within the TOPALi V2.5. software package [107], [110]. In parallel, we reconstructed another phylogenetic tree with maximum likelihood method, based on the JTT matrix-based model [107] with 1000 bootstrap replicates. We imported all consensus trees to MEGA 4 software [111], where we edited and visualized these trees as required.

### Sequence logo creation

We created sequence logos using weblogo 3.0 [112] for the purpose of representing codon usage of DRY motifs.

## Supporting Information

Figure S1 **Comprehensive protein alignment of MC receptors from evolutionary important organisms.** There are three MC receptors such MC1R, MC3R and MC3R were detected from elephant shark (*C. milii*) genome. Lampreys *L. fluviatilis* and *P. marinus* have two copies of MC receptors named as MCAR and MCBR. There are total 69 protein sequences of MC receptors used in this alignment and all of these MCR have conserved DRY motif (marked as ###) at the end of transmembrane helix 3 (TM3). Seven transmembrane regions are marked as TM1–TM7 (yellow bars) as predicted by TMHMM2.0 [106]. Residues conserved above 70% are marked by white on black background.
(PDF)

Figure S2 **Chromosomal mapping of MC1 receptors.** This figure illustrates that MC1R ortholog is found conserved from teleost fishes to mammals.
(TIFF)

Figure S3 **Micro-synteny analysis of MC3 receptors.** **A.** Ortholog of MC3R is conserved in tetrapods. **B.** MC3R like gene is found in only zebrafish whereas other ray-finned fishes have another thyrotropin-releasing hormone receptor 3 (TRHR3) gene at this locus instead of MC3R. *Takifugu* has two copies of TRHR3, which are named as TRHR3a-b.
(TIFF)

Figure S4 **Micro-synteny analysis of MC4R genes depicting presence of orthologs of MC4R gene from fish to mammals.**
(TIFF)

Figure S5 **Intron insertions in MC5 receptor during diversification of ray-finned fishes.** There are three intron inserted at positions 41a, 77c and 140c (numbering human MC5R with suffix a–c for intron phasing; blue background) in MC5Rs of four fishes - *Takifugu*, *Tetraodon*, stickleback and medaka, but not in MC5R like genes from zebrafish, elephant shark and tetrapods. Transmembrane regions are marked as TM1–TM7 (yellow bars) as predicted by TMHMM2.0 [106]. Residues conserved above 70% are marked by white on black background. In mouse and rat MC5R, there is one intron inserted in N-terminal extension marked as 34c-MMU (numbering according to mouse MC5R; red background).
(PDF)

Figure S6 **Analysis of intron insertions in MC2R from selected ray-finned fishes.** There is one intron inserted at position 140c (numbering human MC5R with suffix a–c for intron phasing; blue background) in MC2Rs of four fishes - *Takifugu*, *Tetraodon*, stickleback and medaka, but not in zebrafish and tetrapods. This intron is also conserved in MC5Rs of these selected ray-finned fishes. Takifugu and stickleback has one intron inserted in their MC2R at intron positions 230c (or 225c-TRU, according to *Takifugu* MC2R numbering) and 236a, respectively. Transmembrane regions are marked as TM1–TM7 (yellow bars) as predicted by TMHMM2.0 [106]. Residues conserved above 70% are marked by white on black background.
(PDF)

Figure S7 **Intron insertions in P2Y2 receptor during diversification of ray-finned fishes.** There are three introns inserted at positions 124a, 181c and 267a (human MC5R amino acid numbering with suffix a–c for intron phasing) in P2Y2 receptor of four fishes - *Takifugu*, *Tetraodon*, stickleback and medaka (blue background), but not in P2Y2 genes from zebrafish, elephant shark and tetrapods. Residues conserved above 70% are marked by white on black background. ### indicates location of highly conserved DRY motif. Transmembrane regions are marked as TM1–TM7 (yellow bars) as predicted by TMHMM2.0 [106].
(PDF)

Figure S8 **Intron insertions in P2Y3-like (P2Y3L) receptor during diversification of ray-finned fishes.** One novel intron is inserted at position 187c (human MC5R amino acid numbering with suffix a–c for intron phasing) in the P2Y3L receptor of four fishes - *Takifugu*, *Tetraodon*, stickleback and medaka (blue background), but not in P2Y3L genes from zebrafish, elephant shark and tetrapods. There is also species-specific introns in the P2Y3L gene such as S212a for stickleback, M234a in medaka and T252a for *Takifugu* (numbering is species specific numbering, due to gaps in the region and red background), localized in the loop between TM5 and TM6. Residues conserved above 70% are marked by white on black background. ### indicates location of highly conserved DRY motif. *Gallus* P2Y3 receptor was used as it a typical representative of tetrapod P2Y3. Transmembrane regions are marked as TM1–TM7 (yellow bars) as predicted by TMHMM2.0 [106].
(PDF)

Figure S9 **Intron insertions in P2Y6 receptor during diversification of ray-finned fishes.** One novel intron is inserted at positions 120c (human MC5R amino acid numbering with suffix a–c for intron phasing) in the P2Y6 receptor of four fishes - *Takifugu*, *Tetraodon*, stickleback and medaka (blue background), but not in P2Y2 genes from zebrafish, and tetrapods. Residues conserved above 70% are marked by white on black background. ### indicates location of highly conserved DRY motif. Transmembrane regions are marked as TM1–TM7 (yellow bars) as predicted by TMHMM2.0 [106].
(PDF)

Figure S10 **Intron insertions in CHRM3 receptor during diversification of ray-finned fishes.** There are two introns inserted at positions 67a, 166c (DRY intron or 140c by human MC5R numbering) in CHRM3 receptor of four fishes - *Takifugu*, *Tetraodon*, stickleback and medaka (blue background), but not in P2Y2 genes from zebrafish, elephant shark and tetrapods. Various species-specific introns in the large loop between TM5 and TM6 are marked by red background. Residues conserved above 70% are marked by white on black background. ### indicates location of highly conserved DRY motif. In this case, human CHRM3 amino acid numbering was followed as human MC5R was not suitable due to variable size of these receptor proteins with suffix a–c for intron phasing. Transmembrane regions are marked as TM1–TM7 (yellow bars) as predicted by TMHMM2.0 [106].
(PDF)

Table S1 **List of MC receptors from selected genomes, identified from Ensembl database release 59 (August 2010).** At times data is gathered from additional databases as indicated.
(XLS)

Table S2 **List of CHRM3 genes from fishes.** Codon usage in DRY motif is shown and codons of the R residue is marked by red color. The absence of intron is marked by grey background in R residue.
(DOC)

Table S3 **List of selected GPCRs used in this study, other than MC receptors.** Codon usage in the DRY motif is shown and codon usage of the R residue is marked by red color. Proto-splice site forming codons are indicated by bold letters. The presence and the absence of intron is marked by blue background and grey background for R residue respectively.
(DOC)

Table S4 **List of GPCRs sharing a common DRY intron from different vertebrates.** Vertebrates with compacted genomes are marked in yellow background. Codon usage of DRY motif is shown with | as point of intron insertion. Only mammalian PRLHR gene has no intron, shown in orange background. # Only partial sequence available that renders detection of DRY motif. *Upon BLAT search in Tetraodon Genome browser (http://www.genoscope.cns.fr/externe/tetranew/) using Takifugu NMBR, we obtained putative full length NMBR from Tetraodon localized as fragements on chrUn_random in current assembly of tetraodon genome. ^@^Accession id from Ensembl. ^$^Gene is partial in databases starting from the middle of DRY motif.
(XLSX)

Table S5 **List of genomes used in this study.**
(DOC)

File S1 **Sequences of novel introns inserted into MC receptors of selected ray-finned fishes.** Only introns mapping to core MCR domain (TM1–7) are considered. The numbering of intron positions refers to the full length amino acid numbering of human MC5R.
(PDF)
