# Supplementary material for: Spliceosomal Intron Insertions in Genome Compacted Ray-Finned Fishes as Evident from Phylogeny of MC Receptors, Also Supported by a Few Other GPCRs
Source: PLoS One. 2011 Aug 5;6(8):e22046. doi: 10.1371/journal.pone.0022046 (PMC3151243; doi:10.1371/journal.pone.0022046)
Supplement: Figure S1 — Comprehensive protein alignment of MC receptors from evolutionary important organisms. There are three MC receptors such MC1R, MC3R and MC3R were detected from elephant shark (C. milii) genome. Lampreys L. fluviatilis and P. marinus have two copies of MC receptors named as MCAR and MCBR. There are total 69 protein sequences of MC receptors used in this alignment and all of these MCR have conserved DRY motif (marked as ###) at the end of transmembrane helix 3 (TM3). Seven transmembrane regions are marked as TM1–TM7 (yellow bars) as predicted by TMHMM2.0 [106]. Residues conserved above 70% are marked by white on black background. (PDF) [file pone.0022046.s001.pdf]

Figure S1.

```
MC1R-Human        -----------------------------------------MAVQG----    5
MC1R-Mouse        -----------------------------------------MSTQE----    5
MC1R-Rat          -----------------------------------------MPTQG----    5
MC1R-Opossum      -----------------------------------------MPMPG----    5
MC1R-Chicken      -----------------------------------------MSMLA----    5
MC1R-Zebrafinch   -----------------------------------------MATLA----    5
MC1R-AnoleLizard  -----------------------------------------NATLA----    5
MC1R-Xenopus      --------------------------------------------------    -
MC1R-Takifugu     -----------------------------------------YSTLT----    5
MC1R-Tetraodon    ---------------------------------------MEISNRSLHGS   11
MC1R-Medaka       -----------------------------------------------LFE    3
MC1R-Sickleback   -------------------------------------MELTNRSRL----    9
MC1R-Zebrafish    -----------------------------------------MNDSSRHHF    9
MC1R-Callorhinchus --------------------------------------------------    -
MC2R-Human        --------------------------------------------------    -
MC2R-Mouse        --------------------------------------------------    -
MC2R-Rat          ----------------------------------------MKHIIN----    6
MC2R-Opossum      --------------------------------------------------    -
MC2R-Chicken      -----------------------------------------MSTEK----    5
MC2R-Zebrafinch   -----------------------------------------MSTER----    5
MC2R-Turkey       --------------------------------------------------    -
MC2R-AnoleLizard  --------------------------------------------------    -
MC2R-Takifugu     --------------------------------------------------    -
MC2R-Tetraodon    --------------------------------------------------    -
MC2R-Sickleback   --------------------------------------------------    -
MC2R-Medaka       --------------------------------------------------    -
MC2R-Zebrafish    --------------------------------------------------    -
MC3R-Human        ----------------------------------------MNASCC----    6
MC3R-Mouse        ----------------------------------------MNSSCC----    6
MC3R-Rat          ----------------------------------------MNSSCC----    6
MC3R-AnoleLizard  --------------------------------------------------    -
MC3R-Xenopus      -----------------------------------------MNTTN----    5
MC3R-Zebrafinch   --------------------------------------------------    -
MC3R-Turkey       ----------------------------------------MNSTHF----    6
MC4R-Xenopus      ----------------------------------------MNFTHH---H    7
MC3R-Zebrafish    ----------------------------------------MNDSHL----    6
MC3R-Callorhinchus ----------------------------------------MNSTHF----    6
MC4R-Human        ----------------------------------------MVNSTH-RGM    9
MC4R-Mouse        ----------------------------------------MNSTHH-HGM    9
MC4R-Rat          ----------------------------------------MNSTHH-HGM    9
MC4R-Opossum      ----------------------------------------MNSTHYYHGM   10
MC4R-Chicken      ----------------------------------------MNFTQH-RGT    9
MC4R-Turkey       ----------------------------------------MNFTQH-RGT    9
MC4R-Zebrafinch   ----------------------------------------MNFTQH-RGT    9
MC4R-AnoleLizard  ----------------------------------------MNFTYH-YRV    9
MC4R-Takifugu     --------------------------------------------------    -
MC4R-Tetraodon    -----------------------------------------MNATD----    5
MC4R-Sickleback   ----------------------------------------MNSTQS--GG    8
MC4R-Medaka       ----------------------------------------MNSTLP----    6
MC4R-Zebrafish    ----------------------------------------MNTSHH----    6
MC5R-Human        ----------------------------------------MNSSFH----    6
MC5R-Mouse        MQDQSPVNRRFNSQKPPGTREESCLPLRGAEQNGKSDAKKWGHSLPAMNS   50
MC5R-Rat          ------------SQKPPGTREESCLPLRGAEQNGKFEAKKWRHFLPAMNS   38
MC5R-Opossum      ----------------------------------------MNSSIF----    6
MC5R-Chicken      ----------------------------------------MNTSSQ----    6
MC5R-Zebrafinch   --------------------------------------------------    -
MC5R-Turkey       ----------------------------------------MNTSSQ----    6
MC5R-Takifugu     ---------------------------------MNTSHRSSDPQEG----   13
MC5R-Tetraodon    ---------------------------------MNATHGSSNPQEG----   13
MC5R-Sickleback   ---------------------------------MNLSDESSYREELLLGN   17
MC5R-Medaka       --------------------------------TAPMEVTDKTNSLHKAQL   18
MC5Rb-Zebrafish   ----------------------------------------MNSSEW----    6
MC5Ra-Zebrafish   ----------------------------------------MNTSET----    6
MC5R-Callorhinchus --------------------------------------------------    -
MCAR-Lampetra     ----------------------------------------MNLSEA----    6
MCAR_Lamprey      --------------------------------------------------    -
MCBR-Lampetra     ---------------------------------------MTFSAGGVGGV   11
MCBR_Lamprey      --------------------------------------------------    -
MCRL-Branchiostoma --------------------------------------------------    -
```

```
MC1R-Human          -SQRRLLGSLNSTPTAI---PQLGLAANQTGARC-LEVSISDGLFLSLGL     50
MC1R-Mouse          -PQKSLLGSLNSNA--T---SHLGLATNQSEPWC-LYVSIPDGLFLSLGL     48
MC1R-Rat            -PPKRLLGSLNSTSITT---SHLGLATNQTGSWC-LHVSIPDGLFLSLGL     50
MC1R-Opossum        -QQKRLFNSLNSTSPDT---LHQAVPTNQTDISC-QGLFIPDELFLTLGL     50
MC1R-Chicken        --PLRLLREPWNASE-G---NQSNATAGAGGAWC-QGLDIPNELFLTLGL     48
MC1R-Zebrafinch     --PLRLLREPSNASE-G---NQSNATVGASAGRC-QGLDIPNELFLALGL     48
MC1R-AnoleLizard    ----------------G---NLSVNGTEGGNCSTDNPLMIPHEVFLILGA     36
MC1R-Xenopus        -----MLHSTVNSTNAT---INVGTELKPTNTSD-TVMDVPEELFLFLCV     41
MC1R-Takifugu       ---HTLLLCPLIEFMDD---NETNITNGEQNLGC-VQILIPQELFLTLGL     48
MC1R-Tetraodon      NILHMEFSPLIEFMEDN---ETNITNGDQNTLGC-VQTRIPQELFLTLGI     57
MC1R-Medaka         LKPLKILSQCFLKEKYN---ETNSTAGERNLLGC-FQIRIPQELFLTLGL     49
MC1R-Sickleback     GPSIYHMELSPQDYFME---DNETNSTAGERNGC-VQIRIPQELFLALGL     55
MC1R-Zebrafish      SMKHMDYMYNADNNITL---NSNSTASDINVTGI-AQIMIPQELFLMLGL     55
MC1R-Callorhinchus  ----------------------------MSAVAC-QHVSVPEEVFLSLGI     21
MC2R-Human          --MKHIINS-----------YENINNTARNNSDC-PRVVLPEEIFFTISI     36
MC2R-Mouse          --MKHIINS-----------YEHTNDTARNNSDC-PDVVLPEEIFFTISV     36
MC2R-Rat            ----------------S---YEHTNNTARNNSDC-PDVVVPEEIFFTISI     36
MC2R-Opossum        -ERASELIKILGHNGNP---PENITDNATNNTDC-IQVVVPEEVFFAISI     45
MC2R-Chicken        PFNLILSAHAGQTSIPS---LENITDFSLNITDC-NQVVVPEEVFFTVAA     51
MC2R-Zebrafinch     --PSILIKHPGQTSIPS---LENISDFSLNITDC-TQVVVPEEVFFTVAA     49
MC2R-Turkey         ------IKHAGQTSIPS---LENITDFSLNITDC-NQVVVPEEVFFTVAA     40
MC2R-AnoleLizard    ---------------------------------------VPEEIFFIIAT     11
MC2R-Takifugu       -------SSKLQVLKFT---SLAMNATTVNRSDC-PEVNVPIHVFFTIGF     39
MC2R-Tetraodon      --------------------------------------------------      -
MC2R-Sickleback     -----------------------MDTAAANRSDC-PEVRVPVPLFFTIGV     26
MC2R-Medaka         ---------------SN---FEVMNTNSVNRTDC-PEVKVPFLIFFTVGI     31
MC2R-Zebrafish      ----------------M---NPSAESPSSIHTDC-AEVQVPGQVFLVIAV     30
MC3R-Human          --LPSVQPTLPNGSEHL---QAPFFSNQSSSAFC-EQVFIKPEVFLSLGI     50
MC3R-Mouse          --LSSVSPMLPNLSEHP---AAPPASNRSGSGFC-EQVFIKPEVFLALGI     50
MC3R-Rat            --PSSSYPTLPNLSQHP---AAPSASNRSGSGFC-EQVFIKPEVFLALGI     50
MC3R-AnoleLizard    -------------------------------GVC-ELVFIKADVFLSLGI     18
MC3R-Xenopus        VFSVQAVLANATLDP-N---ETLFLSNLSRIGFC-EQVLIKTEVFLTLGI     50
MC3R-Zebrafinch     -------------------------TNRSGDGFC-EQVFIKAEVFLTLGI     24
MC3R-Turkey         --AFSFQPVLLNVTEDA---NDSILNNRSSDGFC-EQVFIKAEVFLALGI     50
MC4R-Xenopus        EPHHLHYRNHSRTVGAG---ANDTKEKGHHSGGCYEQLFVSPEVFVILGI     54
MC3R-Zebrafish      --QFLKGQKSVNSTSLP---PNGSLADSPAGTLC-EQVQIQAEVFLTLGI     50
MC3R-Callorhinchus  -----LFDLQLNGSGDL---NKSSILNRSNPGFC-EQVPIKSEVFLTLGI     47
MC4R-Human          HTSLHLWNRSSYRLHSN---ASESLGKGYSDGGCYEQLFVSPEVFVTLGV     56
MC4R-Mouse          YTSLHLWNRSSYGLHGN---ASESLGKGHPDGGCYEQLFVSPEVFVTLGV     56
MC4R-Rat            YTSLHLWNRSSHGLHGN---ASESLGKGHSDGGCYEQLFVSPEVFVTLGV     56
MC4R-Opossum        HPTLHFWNHSYVLHS-S---ANDTIGKGYLDGGCYEQLFVSPEVFVTLGI     56
MC4R-Chicken        LQPLHFWNHSNGLHR-G---ASEPSAKGHSSGGCYEQLFVSPEVFVTLGI     55
MC4R-Turkey         LQPLHFWNHSNGLHR-G---ASEPSAKGHSSGGCYEQLFVSPEVFVTLGI     55
MC4R-Zebrafinch     LQPLHFWNHSYRLHG-G---ASERTVKGHSSGGCYEQLFVSPEVFVTLGI     55
MC4R-AnoleLizard    HQHLDLWNHSYRLRGGA---MNRSLGKSYTSEGCYEQLFVSPEVFVTLGF     56
MC4R-Takifugu       ----------------------------ESSTGCYEQMLISTEVFLTLGI     22
MC4R-Tetraodon      PPGLIQDYNNGSQTLEA---DFPDEEEKESSAGCYEQLLISTEVFLTLGI     52
MC4R-Sickleback     LIQGYHNRSASGVSPPD---NDLAAEGKDASAGCYEQLLISTEVFLTLGI     55
MC4R-Medaka         ---YGSVPNRSLSSATL---PPDLGGQKDSSAGCYEQLLISTEVFLTLGI     50
MC4R-Zebrafish      HGLHHSFRNHSQGALPV---GKPSHGDRGSASGCYEQLLISTEIFLTLGL     53
MC5R-Human          ----LHFLDLNLNATEG---NLSGPNVKNKSSPC-EDMGIAVEVFLTLGV     48
MC5R-Mouse          SSTLTVLNLTLNASE-D---GILGSNVKNKSLAC-EEMGIAVEVFLTLGL     95
MC5R-Rat            SSHLTLLDLTLNASE-D---NILGQNVNNKSSAC-EDMGIAVEVFLTLGL     83
MC5R-Opossum        ----LHTLDLNLSSLGG---NMSGPMIKSKSSPC-EQVGIAVEVFLTLGI     48
MC5R-Chicken        ----LYVSELNLSAFGS---NFTVPTVKSKSSPC-EQVVIAAEVFLTLGI     48
MC5R-Zebrafinch     --------------------------------SPC-EQVVIAAEVFLTLGI     18
MC5R-Turkey         ----LYVSELNLSAFGS---NFTVPTVKSKSSPC-EQVVIAAEVFLTLGI     48
MC5R-Takifugu       IMGNSTWNPLSYQPNFT---LSPPLLPKTKTAAC-EQLHIAIEVFLTLGI     59
MC5R-Tetraodon      ILGNSTWNSLFHQPNSS---LSPPPLPKNKTAAC-EQLHIAIEVFLILGI     59
MC5R-Sickleback     FTLAYSYHHQNYTPVPP---LHPDKTGTSKPAAC-EQVHIAIEVFLILGI     63
MC5R-Medaka         ANSTWNFYQQNYTIAPP---SLKDKTSPSAAAAC-EQVHIAVEVFLILGI     64
MC5Rb-Zebrafish     --PTLSPNSSLSQANLS---DESSRPKTSASAAC-EQVHIAPEVFLTLGL     50
MC5Ra-Zebrafish     --TLPFWGMHVNSSPASYILNATETPSHNKPKAC-EQLNIATEVFLILGI     53
MC5R-Callorhinchus  ---------LANVTTAV---MNTSGFMNGSGGIC-RQLEIPLEVYLILGG     37
MCAR-Lampetra       LFPNPFVGTSGPDDN-G---TASASANRTRFSPC-HNFSIPTEVFLALGI     51
MCAR_Lamprey        --------------N-G---TAVASANRTRFSPC-HNFSIPNEVFLTLGI     31
MCBR-Lampetra       VNNHHHGANHQGGGNHS---GHGNATGGGHGRPC-EQVLIPIEVFLILGV     57
MCBR_Lamprey        ----------------------------------C-EQVLIPVEVFLILGV     16
MCRL-Branchiostoma  ------------------------------------------------VGA      3
```

```
                    TM1      |           |       TM2         |
MC1R-Human          VSLVENALVVATIAKNRNLHSPMYCFICCLALSDLLVSGSNVLETAV-IL   99
MC1R-Mouse          VSLVENVLVVIAITKNRNLHSPMYYFICCLALSDLMVSVSIVLETTI-IL   97
MC1R-Rat            VSLVENVLVVVAITKNRNLHSPMYYFICCLALSDLMVSVSIVLETTI-IL   99
MC1R-Opossum        VSLVENMMVVVAIIKNRNLHSPMYYFVCCLALSDLLVSVSNLLETSV-ML   99
MC1R-Chicken        VSLVENLLVVAAILKNRNLHSPTYYFICCLAVSDMLVSVSNLAKTLF-ML   97
MC1R-Zebrafinch     VSLVENLLVVAAILKNRNLHSPTYYFIGCLAVSDMLVSISNLAEMLF-ML   97
MC1R-AnoleLizard    ISFVENLLVVAAIAKNHNLHSPMYYFICCLAMSDMLVSISNVVETFF-ML   85
MC1R-Xenopus        FSLLENILVVIAIFRNHNLHSPMYYFICCLAASDMLVSSSNLGETLI-IF   90
MC1R-Takifugu       ISLVENILVILAIMKNRNLHSPMYYFICCLALSDMLVSVSNVVETVF-ML   97
MC1R-Tetraodon      ISLVENILVILAIIRNRNLHSPMYYFICCLAVSDMLVSVSNVVETIF-ML  106
MC1R-Medaka         ISLVENILVILAIIRNRNLHSPMYYFICCLAVSDMLVSVSNVVETIV-ML   98
MC1R-Sickleback     ISLMENILVVLAILKNRNLHSPMYYFICCLAVSDMLVSVSNVVETIF-ML  104
MC1R-Zebrafish      ISLVENILVVVAIIKNRNLHSPMYYFICCLAVADMLVSVSNVVETLF-ML  104
MC1R-Callorhinchus  VSLVENILVMTAIIKNRNLHSPMHYFICCLAAADMLVSVSNMVETIV-LI   70
MC2R-Human          VGVLENLIVLLAVFKNKNLQAPMYFFICSLAISDMLGSLYKILENIL-II   85
MC2R-Mouse          IGILENLIVLLAVIKNKNLQSPMYFFICSLAISDMLGSLYKILENIL-IM   85
MC2R-Rat            IGVLENLIVLLAVIKNKNLQSPVYFFICSLAISDMLGSLYKILENIL-IM   85
MC2R-Opossum        IGVLENLLVLLAVIKNRNLHSPMYFFICSLAVSDMLGSLYKILENIL-II   94
MC2R-Chicken        AGILENLLVLVAVIRNKNLHLPMYFFICSLAISDMLGSLYKTLENIF-II  100
MC2R-Zebrafinch     AGILENLLILIAVVRNKNLHLPMYFFICSLAISDMLGSLYKTLENIF-II   98
MC2R-Turkey         AGILENLLVLVAVIRNKNLHLPMYFFICSLAISDMLGSLYKTLENIF-II   89
MC2R-AnoleLizard    LGLLENLLVLIAVGRNKNLHSPMYIFICSLAVSDMLGSLYKAVENIFYII   61
MC2R-Takifugu       VSLLENLLVIGAISWNRNLHSPMYCFIGSLAAFNTVASVTKTWENLM-IT   88
MC2R-Tetraodon      ----------------------------------------------VM-IM    4
MC2R-Sickleback     VSLAENLLVVVAVIRNRNLHSPMYCFICSLAAFNTIASLTKTWENLM-IV   75
MC2R-Medaka         VSLAENLLVVLAVILNRNLHSPMYCFICSLAAFNTIASLTKTWENLM-IV   80
MC2R-Zebrafish      ASLSENLLVIVAVIKNKNLHSPMYCFICNLAVFNTISSFSKALENIL-LL   79
MC3R-Human          VSLLENILVILAVVRNGNLHSPMYFFLCSLAVADMLVSVSNALETIM-IA   99
MC3R-Mouse          VSLMENILVILAVVRNGNLHSPMYFFLCSLAAADMLVSLSNSLETIM-IA   99
MC3R-Rat            VSLMENILVILAVVRNGNLHSPMYFFLCSLAAADMLVSLSNSLETIM-IV   99
MC3R-AnoleLizard    VGLLENIFVILAVIKNRNLHSPMYFFLCSLAAADMLVSISNSLETIM-IV   67
MC3R-Xenopus        ISLLENILVILAILKNKNLHSPMYFFLCSLAVADMLVSVSNALETIV-IA   99
MC3R-Zebrafinch     ISLLENILVILAVLKNGNLHSPMYFFLCSLAVADMLVSMSNALETVM-IA   73
MC3R-Turkey         ISLLENILVILAVLKNGNLHSPMYFFLCSLAVADMLVSMSNALETVM-IA   99
MC4R-Xenopus        VSLMENILVIAAISRNKNLHSPMYFFICSLAVADMLVSVSNGFETVV-IT  103
MC3R-Zebrafish      VSLLENILVISAVVKNKNLHSPMYFFLCSLAAADMLVSVSNSLETIV-IA   99
MC3R-Callorhinchus  ISLLENILVILAILKNKNLHSPMYFFLCSLAVADMLVSVSNALETIV-MA   96
MC4R-Human          ISLLENILVIVAIAKNKNLHSPMYFFICSLAVADMLVSVSNGSETIV-IT  105
MC4R-Mouse          ISLLENILVIVAIAKNKNLHSPMYFFICSLAVADMLVSVSNGSETIV-IT  105
MC4R-Rat            ISLLENILVIVAIAKNKNLHSPMYFFICSLAVADMLVSVSNGSETIV-IT  105
MC4R-Opossum        ISLLENILVIVAIAKNKNLHSPMYFFICSLAVADMLVSVSNGSETIV-IT  105
MC4R-Chicken        ISLLENVLVIVAIAKNKNLHSPMYFFICSLAVADMLVSVSNGSETIV-IT  104
MC4R-Turkey         ISLLENVLVIVAIAKNKNLHSPMYFFICSLAVADMLVSVSNGSETIV-IT  104
MC4R-Zebrafinch     ISLLENVLVIVAIAKNKNLHSPMYFFICSLAVADMLVSVSNGSETIV-IT  104
MC4R-AnoleLizard    VSLLENVLVIVAIAKNKNLHSPMYFFICSLAVADLLVSVSNGSETIV-IT  105
MC4R-Takifugu       ISLLENILVVAAIVKNKNLHSPMYFFICSLAVADMLVSVSNASETIV-IA   71
MC4R-Tetraodon      VSLLENILVVAAIVKNKNLHSPMYFFICSLAVADMLVSVSNASETIV-IA  101
MC4R-Sickleback     ISLLENILVVAAIVKNKNLHSPMYFFICSLAVADMLVSVSNASETIV-IA  104
MC4R-Medaka         ISLLENILVVAAIVKNKNLHSPMYFFICSLAVADMLVSVSNASETIV-IA   99
MC4R-Zebrafish      VSLLENILVIAAIVKNKNLHSPMYFFICSLAVADLLVSVSNASETVV-MA  102
MC5R-Human          ISLLENILVIGAIVKNKNLHSPMYFFVCSLAVADMLVSMSSAWETIT-IY   97
MC5R-Mouse          VSLLENILVIGAIVKNKNLHSPMYFFVGSLAVADMLVSMSNAWETVT-IY  144
MC5R-Rat            VSLLENILVIGAIVKNKNLHSPMYFFVGSLAVADMLVSMSNAWETIT-IY  132
MC5R-Opossum        VSLLENILVIGAIVKNKNLHSPMYFFVCNLAVADMLVSVSNAWETIT-IY   97
MC5R-Chicken        VSLLENILVICAIVKNKNLHSPMYFFVCSLAVADMLVSVSNAWETIT-IY   97
MC5R-Zebrafinch     VSLLENILVICAIVKNKNLHSPMYFFVCSLAVADMLVSVSNAWETIT-IY   67
MC5R-Turkey         VSLLENILVICAIVKNKNLHSPMYFFVCSLAVADMLVSVSNAWETIT-IY   97
MC5R-Takifugu       ISLLENILVIMAIVKNKNLHSPMYFFVCSLAVADMLVSVSNASETII-IY  108
MC5R-Tetraodon      ISLLENILVITAIVKNKNLHSPMYFFVCSLAVADMLVSVSNAWETII-IY  108
MC5R-Sickleback     ISLLENILVITAIVKNKNLHSPMYFFVCSLAVADMLVSVSNAWETIV-IY  112
MC5R-Medaka         VSLLENILVITAIVKNKNLHSPMYFFVCSLAVADMLVSVSNAWETII-IY  113
MC5Rb-Zebrafish     ISLLENILVILAIVKNKNLHSPMYFFVCSLAVADMLVSVSNAWETIV-IH   99
MC5Ra-Zebrafish     VSLLENILVICAIVKNKNLHSPMYFFVCSLAVADMLVSVSNAWETIV-IY  102
MC5R-Callorhinchus  VGMLENLLVIIAVVNNRNLHSPMYLFICSLAMADMLVSVGKASEAVI-IF   86
MCAR-Lampetra       VSLVENALVIAAIARNRNMHSPMYCFICSLAVADLLVCLSNAWETIA-IA  100
MCAR_Lamprey        VSLVENALVIAAIARNRNMHSPMYCFICSLAVADLLVCLSNAWETIA-IA   80
MCBR-Lampetra       ISLLENILVITAILKNKNLHSPMYYFICSLAVADMLVSVSNAWETII-MA  106
MCBR_Lamprey        ISLLENILVITAILKNKNLHSPMYYFICSLAVADMLVSVSNAWETII-MA   65
MCRL-Branchiostoma  ASILMNGLVLSGIFRNPHLKEPMYVFVANLAAADCVAGLFSFFFCAI---   50
```

```
                                                                TM3
MC1R-Human             LLEAGALVARAAVLQQLDNVIDVITCS---SMLSSLCFLGAIAVDRYISI   146
MC1R-Mouse             LLEAGILVARVALVQQLDNLIDVLICG---SMVSSLCFLGIIAIDRYISI   144
MC1R-Rat               LLEAGILVARAALVQQLDNVIDVLICG---SMVSSLCFLGVIAIDRYISI   146
MC1R-Opossum           LLEKGVLMIQMPMLQQLDNVIDVLICG---SMMSSISFLGAIAVDRYISI   146
MC1R-Chicken           LMEHGVLVIRASIVRHMDNVIDMLICS---SVVSSLSFLGVIAVDRYITI   144
MC1R-Zebrafinch        LLEHGVLVMRPSIVRHMDSVIDTLICS---SVVSSLSFLGVIAVDRYITI   144
MC1R-AnoleLizard       LNDHGLLLLGKSTIKHMDIIMDMLICS---SLLSSLSFLSVIAIDRYITI   132
MC1R-Xenopus           MLKQGIIKSEPLLVKKMDYIFDTMICC---SLVTSLSFLGAIAIDRYITI   137
MC1R-Takifugu          LNDHGLMDMYPGMLRHLDNVIDVMICS---SVVSSLSFLCTIAADRYITI   144
MC1R-Tetraodon         LNDHGLMDMYPGMLRHLDNVIDAMICS---SVVSSLSFLCTIAADRYITI   153
MC1R-Medaka            LTDHGLLDVHPGMLRHLDNVIDVMICS---SVVSSLSFLCTIAADRYITI   145
MC1R-Sickleback        LNDHGLMDVHPGMLRHLDNVIDVMICS---SVVSSLSFLCTIAADRYITI   151
MC1R-Zebrafish         LTEHGLLLVTAKMLQHLDNVIDIMICS---SVVSSLSFLCTIAADRYITI   151
MC1R-Callorhinchus     LMERGVMVVQNYLLKQIDNLIDMMICS---SMVSSLSFLGAIAADRYITI   117
MC2R-Human             LRNMGYLKPRGSFETTADDIIDSLFVL---SLLGSIFSLSVIAADRYITI   132
MC2R-Mouse             FRNMGYLKPRGSFESTADDIIDCMFIL---SLLGSIFSLSVIAADRYITI   132
MC2R-Rat               FRNMGYLKPRGSLESTADDIIDCMFVL---SLLGSIFSLSVIAADRYITI   132
MC2R-Opossum           FRNTGYLKPRGDFETTADDVVDSLFIL---SLLGSIFSLSVIAADRYITI   141
MC2R-Chicken           LCKMGYLTRRGDFEKKLDDAMDSMFIL---SLLGSIFSLLAIAADRYITI   147
MC2R-Zebrafinch        LCKMGYLTRHGDFEKKLDDAMDSMFIL---SLLGSIFSLLAIAADRYITI   145
MC2R-Turkey            LCKMGYLTRRGDFEKKLDDAMDSMFIL---SLLGSIFSLLAIAADRYITI   136
MC2R-AnoleLizard       FCKMQYVKCRGKLAKTMDDILDFMFIL---SLLGSIFSLSAIAADRYITI   108
MC2R-Takifugu          FAEVGHLRKVGFSERKADDVVDSLLCM---SFLGSIFSFLAIAVDRYITI   135
MC2R-Tetraodon         FADVGHLRKVGYLELKLDDVVDSLLCM---SFLGSIFSFLAIAGDRYITI    51
MC2R-Sickleback        FANIGLLEKKGPSETNVDDVLDSLLCM---SFVGSIVSFLAIAADRYISI   122
MC2R-Medaka            FADVGQLEKRGPSETKLDDMMDSLLCM---SFVGSIFSFLAIAVDRYITI   127
MC2R-Zebrafish         FKDAGRLNSRGPFELKIDDIMDSLLCM---CFLGSIFSILAIAVDRYISI   126
MC3R-Human             IVHSDYLTFEDQFIQHMDNIFDSMICI---SLVASICNLLAIAVDRYVTI   146
MC3R-Mouse             VINSDSLTLEDQFIQHMDNIFDSMICI---SLVASICNLLAIAIDRYVTI   146
MC3R-Rat               VINSDSLTLEDQFIQHMDNIFDSMICI---SLVASICNLLAIAVDRYVTI   146
MC3R-AnoleLizard       LLEKGYLNINSRYIQPMDNVFDSMICI---SLIGSICNLLVIAIDRYITI   114
MC3R-Xenopus           I-QNKYLVIGDYLLQHLDDVFDSMICI---SLVASICNLLVIAIDRYITI   145
MC3R-Zebrafinch        ILSNGYLIIADHFIQHMDNVFDSMICI---SLVASICNLLVIAIDRYITI   120
MC3R-Turkey            ILSSGYLIIDDHFIQHMDNVFDSMICI---SLVASICNLLVIAIDRYITI   146
MC4R-Xenopus           LFNTTDKNTQ-HIIVNVDNILDSVICS---SLLASICSLLSIAVDRYFTI   149
MC3R-Zebrafish         VLNSRLVVASDQFVRLMDNVCDSMICI---SLVASICNLLAIAVDRYVTI   146
MC3R-Callorhinchus     LLNNGYLVANDQFIQIMDNVIDSLICI---SLVASICNLLVIAIDRYITI   143
MC4R-Human             LLNSTDTDAQ-SFTVNIDNVIDSVICS---SLLASICSLLSIAVDRYFTI   151
MC4R-Mouse             LLNSTDTDAQ-SFTVNIDNVIDSVICS---SLLASICSLLSIAVDRYFTI   151
MC4R-Rat               LLNSTDTDAQ-SFTVNIDNVIDSVICS---SLLASICSLLSIAVDRYFTI   151
MC4R-Opossum           LLNSTDTDAQ-SFTVNIDNVIDSVICS---SLLASICSLLSIAVDRYFTI   151
MC4R-Chicken           LLNNTDTDAQ-SFTINIDNVIDSVICS---SLLASICSLLSIAVDRYFTI   150
MC4R-Turkey            LLNNTDTDAQ-SFTINIDNVIDSVICS---SLLASICSLLSIAVDRYFTI   150
MC4R-Zebrafinch        LLNNTDTDAQ-SFTINIDNVIDSVICS---SLLASICSLLSIAVDRYFTI   150
MC4R-AnoleLizard       LLNNTDVGGH-SFTVSIDNIIDSVICS---SLLASICSLLSIAVDRYFTI   151
MC4R-Takifugu          LINSGTLTIPATLIKSMDNVFDSMICS---SLLASICSLLAIAVDRYITI   118
MC4R-Tetraodon         LINGGTLTIPARLIKSMDNVFDSMICS---SLLASICSLLAIAVDRYITI   148
MC4R-Sickleback        LIAGGTLTIPVALIRNMDNVFDSMICS---SLLASICSLLAIAVDRYITI   151
MC4R-Medaka            LINGGNLSIPVRLIKSMDNVFDSMICS---SLLASICSLLAIAVDRYITI   146
MC4R-Zebrafish         LITGGNLTNRESIIKNMDNVFDSMICS---SLLASIWSLLAIAVDRYITI   149
MC5R-Human             LLNNKHLVIADAFVRHIDNVFDSMICI---SVVASMCSLLAIAVDRYVTI   144
MC5R-Mouse             LLNNKHLVIADTFVRHIDNVFDSMICI---SVVASMCSLLAIAVDRYITI   191
MC5R-Rat               LINNKHVVIADTFVRHIDNVFDSMICI---SVVASMCSLLAIAVDRYITI   179
MC5R-Opossum           LINNKHLVMEDAFVRHIDNVFDSMICI---SVVASMCSLLAIAVDRYVTI   144
MC5R-Chicken           LINNRHIIMEDAFVRHIDNVFDSLICI---SVVASMCSLLAIAVDRYITI   144
MC5R-Zebrafinch        LINNRHVIMEDAFVRHIDNVFDSMICI---SVVASMCSLLAIAVDRYITI   114
MC5R-Turkey            LINNRHIIMEDAFVRHIDNVFDSLICI---SVVASMCSLLAIAIDRYITI   144
MC5R-Takifugu          LLNNKQLIAEDHLIRQLDNVFDSMICI---SVVASMCSLLAIAVDRYVTI   155
MC5R-Tetraodon         LLNNKQLVVEDHFIQQLDNVFDSMICI---SVVASMCSLLAIAVDRYVTI   155
MC5R-Sickleback        LLNNRQLVVEDHFIRQMDNVFDSMICI---SVVASMCSLLAIAVDRYVTI   159
MC5R-Medaka            LLNNRQLVVEEHFIRQLDNIFDSMICI---SVVASMCSLLAIAVDRYVTI   160
MC5Rb-Zebrafish        LLANRSLVIEDHFIRQMDNVFDSLICI---SVVGSMWSLLAIAVDRYVTI   146
MC5Ra-Zebrafish        LLTNRQLVVEDHFIRQMDNVFDSMICI---SVVASMCSLLAIAVDRYVTI   149
MC5R-Callorhinchus     LDQNSHLLTE-TLIDHLDYLFDSLICI---SLIASILSLGAIATDRYLTI   132
MCAR-Lampetra          LVHGRHVHIPARILQHVDNVFDSFICI---SVVASMCNLLAIAVDRYVTI   147
MCAR_Lamprey           LVHGRHVHIPAPILQHVDNVFDSFICI---SVVASMCNLLAIAVDRYVTI   127
MCBR-Lampetra          LLQNGSLAMQEDTLKQMDNIMDSMICT---SVVASMCSLLAIAVDRYVTI   153
MCBR_Lamprey           LLQNGSLAMQEDTLKQMDNIMDSMICT---SVVASMCSLLAIAVDRYVTI   112
MCRL-Branchiostoma     --------FYGEIMRPITVL--SLFCLFFFVLVLSAVGVILLSVDRYLAI    90
                                                                 ###
```

```
                         |        TM4         |   |
MC1R-Human          FYALRYHSIVTLPRARRAVAAIWVASVVFSTLFIAYYDHVAVLL--CLVV    194
MC1R-Mouse          FYALRYHSIVTLPRARRAVVGIWMVSIVSSTLFITYYKHTAVLL--CLVT    192
MC1R-Rat            FYALRYHSIVTLSRARRAVVGIWVVSIVSSTLFITYYKHTAVLL--CLVT    194
MC1R-Opossum        FYALRYHSIVTPCRAKGVLAGIWVSSAFSGTLFISYYNHNAVLL--CLIG    194
MC1R-Chicken        FYALRYHSIMTLQRAVVTMASVWLASTVSSTVLITYYRNNAILL--CLIG    192
MC1R-Zebrafinch     FYALRYHSIMTLQRAVVTMASVWLASTASSTVLITYYHSNTIRL--CLIG    192
MC1R-AnoleLizard    FYALRYHNIMTFQRALALILSIWAVCFVASIVFIL-NDSPAVIT--CLVV    179
MC1R-Xenopus        FYALRYHSIMTLRRVVIAIGVIWSVSLVCAAIFIVYHESRAVIL--CLIV    185
MC1R-Takifugu       FYALRYHSIMTTPRAITIIVIVWCASIASSILFIVYHTDNAVIV--CLVT    192
MC1R-Tetraodon      FYALRYHSIMTTQRAIAIIVTVWCASITSSTLFIVYHTDNAVIV--CLVA    201
MC1R-Medaka         FYALRYHSIMTSQRAVIIIVLVWLASLISSILFIVHDTDHAVIV--CLIT    193
MC1R-Sickleback     FYALRYHNIMTTQRAVIIIGLVWLASITSSILFIVYHTDIAVIV--CLVT    199
MC1R-Zebrafish      FYALRYHSIMTTQRAVGIILVVWLASITSSSLFIVYHTDNAVIA--CLVT    199
MC1R-Callorhinchus  FYALRYHTIMTTRRAVGVMVGIWVVSITSSAIFIVYSENSAVVI--CLIS    165
MC2R-Human          FHALRYHSIVTMRRTVVVLTVIWTFCTGTGITMVIFSHHVPTVI--TFTS    180
MC2R-Mouse          FHALQYHSIVTMRRTIITLTIIWMFCTGSGITMVIFSHHIPTVL--TFTS    180
MC2R-Rat            FHALQYHSIVTMRRTVITLTVIWMFCTGSGITMVIFSHHIPTVL--TFTS    180
MC2R-Opossum        FHALQYHNIMTMRRAISILAIIWAFCTGSGITMVIFSHDVPTVI--SFTS    189
MC2R-Chicken        FYALRYHNIMTLQRALVILAIIWTFCAGSSIAIALFSHEVATVI--PFTI    195
MC2R-Zebrafinch     FYALRYHNIMTLRRALVILAIIWAFCAGSSIAIALFSYEAATVI--PFTI    193
MC2R-Turkey         FYALRYHNIMTVRRALVILAIIWTFCAGSSIAIALFSHEIATVI--PFTI    184
MC2R-AnoleLizard    FYALRYHNIMTLKRALVMLGVIWAFCTGSGIAMVLFSYEAVTVV--SFTV    156
MC2R-Takifugu       FHALRYHNIMTMQRTGAILGLIWTTCGVSAMLMVRFFDSNLIMS--CFVV    183
MC2R-Tetraodon      FHALRYHDIMTLQRTGAILGVIWTTCGVSAVLMVRFFDSNLIMG--CFVV     99
MC2R-Sickleback     FHALRYHNIMTMRRTRAVLGVIWTLCGVSAVLMVRFFDSNFIMI--CFVV    170
MC2R-Medaka         FNALRYHNIMTMQRTGGILVVIWTTCGVSAVLMVRFFDFKFIMI--CFVV    175
MC2R-Zebrafish      FHALRYHMLMTMRRVLIILFTIWVLCGTSGALMVGFFEAATVKI--FFIV    174
MC3R-Human          FYALRYHSIMTVRKALTLIVAIWVCCGVCGVVFIVYSESKMVIV--CLIT    194
MC3R-Mouse          FYALRYHSIMTVRKALTLIGVIWVCCGICGVMFIIYSESKMVIV--CLIT    194
MC3R-Rat            FYALRYHSIMTVRKALSLIVAIWVCCGICGVMFIVYSESKMVIV--CLIT    194
MC3R-AnoleLizard    FYALRYHSIMTVKKALALIGLIWIACICCGITFIIYSESKMVII--CLIA    162
MC3R-Xenopus        FYALRYHSIMTVKKAIALIVVIWTSCIICGIVFIVFSESKTVIV--CLIT    193
MC3R-Zebrafinch     FYALRYHSIMTVKKALTLIVLIWVACIICGIIFIAYSESKTVIV--CLIT    168
MC3R-Turkey         FYALRYHSIMTVKKALTLIVLIWIACIICGIIFIAYSESKTVIV--CLIT    194
MC4R-Xenopus        FYALQYHNIITVRRAVVIISCIWTACSISGVLFIIYYDSAVVII--CLIS    197
MC3R-Zebrafish      FYALRYHSIVTVRRALVAIAVIWLVCVVCGIVFIVYSESKTVIV--CLIT    194
MC3R-Callorhinchus  FYALRYHSIMTVKRALLLIIVIWIACIFCGIIFIIYSNSKTVII--CLIT    191
MC4R-Human          FYALQYHNIMTVKRVGIIISCIWAACTVSGILFIIYSDSSAVII--CLIT    199
MC4R-Mouse          FYALQYHNIMTVRRVGIIISCIWAACTVSGVLFIIYSDSSAVII--CLIS    199
MC4R-Rat            FYALQYHNIMTVRRVGIIISCIWAACTVSGVLFIIYSDSSAVII--CLIT    199
MC4R-Opossum        FYALQYHNIMTVRRVGIIITCIWAACTVSGILFIIYSDSSAVII--CLIT    199
MC4R-Chicken        FYALQYHNIMTVKRVGVIITCIWAACTVSGILFIIYSDSSVVII--CLIS    198
MC4R-Turkey         FYALQYHNIMTVKRVGVIITCIWAACTVSGILFIIYSDSSVVII--CLIS    198
MC4R-Zebrafinch     FYALQYHNIMTVKRVGVIITCIWAACTVSGILFIIYSDSSVVII--CLIS    198
MC4R-AnoleLizard    FYALQYHNIMTVRRVGIIITCIWAACTVSGILFIIYSDSSVVII--CLIS    199
MC4R-Takifugu       FYALRYHNIVTLRRASLVISSIWTCCTVSGVLFIVYSESTTVLI--CLIT    166
MC4R-Tetraodon      FYALRYHNIVTLRRASLVISSIWTCCTVSGVLFIIYSESTTVLI--CLIT    196
MC4R-Sickleback     FYALRYHNIVTLRRATLVIGSIWTCCTVSGILFIIYSESTSVLI--CLIT    199
MC4R-Medaka         FYALRYHNIVTLRRAAVVISSIWTCCIVSGILFIIYSESTTVLI--CLIT    194
MC4R-Zebrafish      FYALRYHNIMTQRRAGTIITCIWTFCTVSGVLFIVYSESTTVLI--CLIS    197
MC5R-Human          FYALRYHHIMTARRSGAIIAGIWAFCTGCGIVFILYSESTYVIL--CLIS    192
MC5R-Mouse          FYALRYHHIMTARRSGVIIACIWTFCISCGIVFIIYYESKYVII--CLIS    239
MC5R-Rat            FYALRYHHIMTARRSGVIIACIWTFCISCGIVFIIYYESKYVIV--CLIS    227
MC5R-Opossum        FYALRYHNIMTVKRSGIIIACIWTFCTGCGIIFILYYESTYVII--CLIT    192
MC5R-Chicken        FYALRYHNIMTVKRSGLIIACIWTFCTGCGIIFILYYESTYVII--CLIT    192
MC5R-Zebrafinch     FYALRYHNIMTVKRSGLIIACIWTFCTGCGIIFILYYESTYVVI--CLIT    162
MC5R-Turkey         FYALRYHNIMTVKRSGLIIACIWTFCTGCGIIFILYYESTYVII--CLIT    192
MC5R-Takifugu       FYALRYHNIMTVRRAGCIIGGIWTFCTGCGIVFIIYSDTTPVII--CLVC    203
MC5R-Tetraodon      FYALRYHNIMTVRRAGCIIGGIWTFCTGCGIVFIIYSEKTPVII--CLVS    203
MC5R-Sickleback     FYALRYHNIMTVRRAGCIIGGIWTFCTGCGIIFIIYSDTTPVII--CLVS    207
MC5R-Medaka         FYALRYHNIMTMRRAGCIIGGIWIFCTGCGIVFILYSETTPVII--CLVS    208
MC5Rb-Zebrafish     FYALRYHNIMTVRRAGILIGSIWTFSTSCGIIFIIYSDTQPVVV--CLVA    194
MC5Ra-Zebrafish     FYALRYHNIMTVRRAALIIGGIWTFCTGCGIVFIIYSDNTSVIV--CLVS    197
MC5R-Callorhinchus  FHALRYHQIMTVKRAALIISALWTFCTFSGSFIIKFNRKNAFPG--SLIT    180
MCAR-Lampetra       FYALQYHSIVTMRRAAVVIACVWAACVVSGTLFITYWDHRTVIV--CLIA    195
MCAR_Lamprey        FYALQYH-------------------------------------------    134
MCBR-Lampetra       FYALRYHNIMTVRRAASIIGAIWGTCTLCGVIFIVYSDSTAVII--CLIT    201
MCBR_Lamprey        FYALRYHNIMTVRRAASIIGAIWGTCTLCGVIFIVYSDSTAVII--CLIA    160
MCRL-Branchiostoma  LHPIFYQTRISRQHAAVSLGIAWPVCAIACLSPLMGWNCIEIETEDCMTN    140
```

```
                      TM5
MC1R-Human            FFLAMLVLMAVLYVHMLARACQHAQGIARLH----KRQRP-VHQGFGLKG    239
MC1R-Mouse            FFLAMLALMAILYAHMFTRACQHAQGIAQLH----KRRRS-IRQGFCLKG    237
MC1R-Rat              FFLAMLALMAILYVHMLSRACQHAQGIARLH----KRRHS-IRQGFCLKG    239
MC1R-Opossum          FFLSMLGLMVVLYIHMFIQACQHARRIARLH-----KRHA-IHQLSTLKG    238
MC1R-Chicken          FFLFMLVLMLVLYIHMFALARHHVRSISSQQ------KQPTIYRTSSLKG    236
MC1R-Zebrafinch       FFLFMLVLMLVLYIHMFALARHHLHSISSQQ-----KPTT-AYRGGSLKG    236
MC1R-AnoleLizard      FFVAVVTLILALYTHMFILARHHARRMSHIP----KKQN--SPQMSSMKG    223
MC1R-Xenopus          FFLFMLALMVALYIHMFALARQHARSISALQKGKSRRITP-HQARANMKG    234
MC1R-Takifugu         FFCITLVFNAVLYVHMFVLAHVHSRRIMAFH-------KN-RRQSTSMKG    234
MC1R-Tetraodon        FFCTTLVFNAVLYLHMFLLAHVHSRRIMAFH-------KN-RRQSTSMKG    243
MC1R-Medaka           FFFTTLVFNAVLYLHMFILAHVHSRRITAFH-------KS-RRPSTSMKG    235
MC1R-Sickleback       FFCATLVFNAVLYLHMFFLAHVHSRRIIAFN-------KN-RRQSTSMKG    241
MC1R-Zebrafish        FFGVTLVFTAVLYLHMFILAHVHSRRITALH-------KS-RRQTTSMKG    241
MC1R-Callorhinchus    FFFVMVIFMGALYLHMFTLARIHAKRIMAQH-----KKRT-LHQATSMKG    209
MC2R-Human            LFPLMLVFILCLYVHMFLLARSHTRKISTLP-------------RANMKG    217
MC2R-Mouse            LFPLMLVFILCLYIHMFLLARSHARKISTLP-------------RTNMKG    217
MC2R-Rat              LFPLMLVFILCLYIHMFLLARSHARKISTLP-------------RANMKG    217
MC2R-Opossum          LFPLMLIFILCLYVHMFLLARSHAKKIISLP------SSR-VQPRANMKG    232
MC2R-Chicken          LFPLMMIFILCLYIHMFLLARSHAKKIASLP------TSA-VHQRTNMKG    238
MC2R-Zebrafinch       LFPLMMFFILCLYIHMFLLARTHAKKIASLP--------STIHHRTNMKG    235
MC2R-Turkey           LFPLMMFFILCLYIHMFLLARSHAKKIASLP------TGA-VHQRTNMKG    227
MC2R-AnoleLizard      LFCFMLILILCLYIHMFLLARSHAKKIALMT----TSSVH-Q--GANMKG    199
MC2R-Takifugu         FFIISLAIIYILYVYMFILARVHARKIAALPNGSGKHQHQ-RRWGHGMRG    232
MC2R-Tetraodon        FFIISLVIIYFLYVYMFILSRVHARRIAALPGGSGKHQHL-RRWGNGMRG    148
MC2R-Sickleback       FFVVSLAIICFLYVYMFMLARIHARKIAALP------------ASNGMRG    208
MC2R-Medaka           FFIVSLAIICFLYVYMFLLARIHAKSIAALP-------------------    206
MC2R-Zebrafish        LFFTALLLILLLYVHMFLLARHHANRIASMP-----GAQA-QHRKSGLRG    218
MC3R-Human            MFFAMMLLMGTLYVHMFLFARLHVKRIAALP----PADGVAPQQHSCMKG    240
MC3R-Mouse            MFFAMVLLMGTLYIHMFLFARLHVQRIAVLP----PAGVVAPQQHSCMKG    240
MC3R-Rat              MFFAMVLLMGTLYIHMFLFARLHVQRIAALP----PADGVAPQQHSCMKG    240
MC3R-AnoleLizard      MFFTMLFLMASLYVHMFMFARLHIKRIAAFP-----VDDV-PQQRTCMKG    206
MC3R-Xenopus          MFFTMLVLMATMYVHMFLFARLHVKRIAALP-----VDGV-VQQRTCMKG    237
MC3R-Zebrafinch       MFFTMLLLMASLYVHMFLFARLHVKRIAALP-----VEGV-PPQRTCMKG    212
MC3R-Turkey           MFFTMLFLMASLYVHMFLFARLHVKRIAALP-----VDGV-PSQRTCMKG    238
MC4R-Xenopus          IIFTMLALMASLYVHMFMLARLHIKRIAVLP-----GTNS-VRQVTNMKG    241
MC3R-Zebrafish        MFFAMLVLMATLYVHMFLLARLHVQRIAALPPAAPGAGNPAPRQRSCMKG    244
MC3R-Callorhinchus    MFFTMLVLMTTLYVHMFMLARLHIKKIAALP-----VDGI-VRPRTCMKG    235
MC4R-Human            MFFTMLALMASLYVHMFLMARLHIKRIAVLP-----GTGA-IRQGANMKG    243
MC4R-Mouse            MFFTMLVLMASLYVHMFLMARLHIKRIAVLP-----GTGT-IRQGTNMKG    243
MC4R-Rat              MFFTMLVLMASLYVHMFLMARLHIKRIAVLP-----GTGT-IRQGANMKG    243
MC4R-Opossum          MFFTMLALMASLYVHMFLMARLHIKRIAVLP-----GTGT-IRQGANMKG    243
MC4R-Chicken          MFFTMLILMASLYVHMFMMARMHIKKIAVLP-----GTGP-IRQGANMKG    242
MC4R-Turkey           MFFTMLILMASLYVHMFMMARMHIKKIAVLP-----GTGP-IRQGANMKG    242
MC4R-Zebrafinch       MFFTMLILMASLYVHMFMMARMHIKKIAVLP-----GTGP-VRQGANMKG    242
MC4R-AnoleLizard      MFFTMLVLMASLYVHMFLLARLHIKKIAILP-----GTGP-ICQRANMKG    243
MC4R-Takifugu         MFFTMLVLMASLYVHMFLLARLHMKRIAAMP-----GNAP-IHQRANLKG    210
MC4R-Tetraodon        TFFTMLVLMASLYVHMFLLARLHMKRIAAMP-----GNAP-IHQRANMKG    240
MC4R-Sickleback       MFFTMLVLMASLYVHMFLLARLHIKRIAALP-----GHAP-IHQRANMKG    243
MC4R-Medaka           MFFTMLVLMASLYVHMFLLARLHMKRIAALP-----GNAP-IHQRANMKG    238
MC4R-Zebrafish        MFFTMLALMASLYVHMFLLARLHMKRIAALP-----GNGP-IWQAANMKG    241
MC5R-Human            MFFAMLFLLVSLYIHMFLLARTHVKRIAALP-----GASS-ARQRTSMQG    236
MC5R-Mouse            MFFTMLFFMVSLYIHMFLLARNHVKRIAASP-----RYNS-VRQRTSMKG    283
MC5R-Rat              MFFTMLFFMVSLYIHMFLLARNHVKRIAASP-----RYNS-VRQRASMKG    271
MC5R-Opossum          MFFTMLFLMVSLYIHMFLLARTHVKRIAALS-----GYSS-VRQRISMKG    236
MC5R-Chicken          MFFTMLFLMVSLYIHMFLLARTHVKKIAALP-----GYNS-VHQRTSMKG    236
MC5R-Zebrafinch       MFFTMLFLMVSLYIHMFLLARTHVKKIAALP-------------GTSMKG    199
MC5R-Turkey           MFFTMLFLMVSLYIHMFLLARTHVKKIAALP-----GYNS-VHQRTSMKG    236
MC5R-Takifugu         MFFAMLLIMASLYSHMFMLARSHVKRIAALP-----GSNS-IHQRASMKG    247
MC5R-Tetraodon        MFFAMLLIMASLYSHMFMLARSHVKRIAALP-----GSSS-IQQRANMKG    247
MC5R-Sickleback       MFFAMLVIMASLYSHMFMLARSHVKRIAALP-----GYNS-IHQRTSMKG    251
MC5R-Medaka           MFFAMLLIMASLYSHMFMLARSHVKRIAALP-----GYNS-IHQRASMKG    252
MC5Rb-Zebrafish       MFFAMLLMMASLYSHMFLLARSHVKRMAALP-----GYNANIRQRASMKG    239
MC5Ra-Zebrafish       MFFIMLALMASLYSHMFMLARSHVKRIAALP-----GYNS-IHQRASMKA    241
MC5R-Callorhinchus    MYFTTLFVIVSLYVYMFLLARRHAQCIRSLP-----GQR--VHQGTSLKG    223
MCAR-Lampetra         LFVTMLVLMASLYAHMFALARSHAQRISAQP--RSSRQGQ-QNGAASLKG    242
MCAR_Lamprey          ------------------------RRISAQP--RSSRQGQ-QHGAASLKG    157
MCBR-Lampetra         MFFTMLVLMASLYVHMFMLARLHAKRIAALP-----ASGI-IQHKTSMRG    245
MCBR_Lamprey          MFFTMLVLMASLYVHMFMLARLHAKRIAALP-----ASGI-IQHKTSMRG    204
MCRL-Branchiostoma    APVNYLILINAILAENLPAARRREQRVAARE----QRQL-----KLSLKT    181
```

```
                         |       TM6          |              |
MC1R-Human          AVTLTILLGIFFLCWGPFFLHLTLIVLCPEHPTCGCIFKNFNLFLALIIC   289
MC1R-Mouse          AATLTILLGIFFLCWGPFFLHLLLIVLCPQHPTCSCIFKNFNLFLLLIVL   287
MC1R-Rat            AATLTILLGIFFLCWAPFFLHLLLIVLCPQHPTCSCIFKNFNLFLILIIL   289
MC1R-Opossum        AITLMILLGIFFLCWAPFFLHLTLIVLCPKHPTCSCYFQNFNFFLILIIC   288
MC1R-Chicken        AVTLTILLGVFFICWGPFFFHLILIVTCPTNPFCTCFFSYFNLFLILIIC   286
MC1R-Zebrafinch     AVTLTILLGVFFICWGPFFFHLILIVTCPTNPFCTCFFSYFNLFLVLMIC   286
MC1R-AnoleLizard    AITLTILLGVFLICWGPFFLHLTLIIICPFR--CACYFNYFSLYLILIIC   271
MC1R-Xenopus        AITLTLLLGVFFLCWGPLFLHLTLFVSCPGHHICNSYFYYFNIYLLLVIC   284
MC1R-Takifugu       AITLTILLGVFILCWGPFFLHLILILTCPTSVFCNCYFRNFNLFLILIIC   284
MC1R-Tetraodon      AITLTILLGVFIVCWGPFFLHLILILTCPNNPLCNCYFRNFNLFLILIIC   293
MC1R-Medaka         AITLTILLGVFIICWGPFFLHLILTLACPTSPFCNCFFRNFNLFLILIIC   285
MC1R-Sickleback     AMTLTILLGVFIVCWGPFFLHLILILTCPTSPFCNCFFRNFNLFLILIIC   291
MC1R-Zebrafish      AITLTILLGVFILCWGPFFLHLILILTCPTNPYCKCYFSHFNLFLILIIC   291
MC1R-Callorhinchus  AITLTILLGVFLICWSPFFLHLILIILCPTNPYCQCFTSHFNMFLILIIC   259
MC2R-Human          AITLTILLGVFIFCWAPFVLHVLLMTFCPSNPYCACYMSLFQVNGMLIMC   267
MC2R-Mouse          AMTLTILLGVFIFCWAPFVLHVLLMTFCPNNPYCVCYMSLFQVNGMLIMC   267
MC2R-Rat            AMTLTILLGVFIFCWAPFVLHVLLMTFCPNNPYCVCYMSLFQVNGMLIMC   267
MC2R-Opossum        AITLTILLGVFLCCWAPFVLHILLVTFCPNNPYCACYLSIFQVNGMLIMC   282
MC2R-Chicken        AITLTIFLGVFLCCWAPFVLHILLARFCPHNPYCACYMSIFHVNGTLIMC   288
MC2R-Zebrafinch     AITLTIFLGVFLCCWAPFVLHILLARFCPHNPYCACYMSIFHVNGTLIMC   285
MC2R-Turkey         AITLTIFLGVFLCCWAPFVLHILLARFCPHNPYCACYMSIFHVNGTLIMC   277
MC2R-AnoleLizard    AITLTVLVAVFLFCWSPFVLHMLLMTFCPQNPYCICYGSIFHVHGMLIMC   249
MC2R-Takifugu       ILTLTILFGAFMVCWAPFFLHLIFLMACPMNPYCECYRSMFQLHLVLLMS   282
MC2R-Tetraodon      IMTLTILFGAFMVCWAPFFLHLIILMVCPMNPYCECYRSLFELHVVLLMS   198
MC2R-Sickleback     ALTLTILFGAFVVCWAPFFLHLIIIMLCPTNPYCECYRSLFQLHVVLLMS   258
MC2R-Medaka         --------------------------------------------------     -
MC2R-Zebrafish      ALTLTILIGVFVACWAPFSLHLLIMMICPENPYCECYRSLFQLHVVLLVS   268
MC3R-Human          AVTITILLGVFIFCWAPFFLHLVLIITCPTNPYCICYTAHFNTYLVLIMC   290
MC3R-Mouse          AVTITILLGVFIFCWAPFFLHLVLIITCPTNPYCICYTAHFNTYLVLIMC   290
MC3R-Rat            AVTITILLGVFIFCWAPFFLHLVLIITCPTNPYCICYTAHFNTYLVLIMC   290
MC3R-AnoleLizard    AVTITILLGVFIVCWGPFFLHLILIISCPTNPHCFCYISHFNTYLVLIMC   256
MC3R-Xenopus        AITITILLGVFVVCWAPFFLHLILIISCPSNSYCVCYTSYFNTYLILIMC   287
MC3R-Zebrafinch     AVTITILLGVFIVCWAPFFLHLILIISCPTNPYCICYTAHFNAYLVLIMC   262
MC3R-Turkey         AVTITILLGVFIVCWAPFFLHLILIISCPMNSYCVCYTSHFNTYLVLIMC   288
MC4R-Xenopus        AITLTILLGVFVACWSPFFLHLIFYVSCPRNPYCVCFMSHFNIYLILIMC   291
MC3R-Zebrafish      AVTISILLGVFVCCWAPFFLHLILLVSCPHHPLCLCYMSHFTTYLVLIMC   294
MC3R-Callorhinchus  AITITILLGIFIICWAPFFLHLILIISCPKNAYCICYTSHFNTYLILIMC   285
MC4R-Human          AITLTILIGVFVVCWAPFFLHLIFYISCPQNPYCVCFMSHFNLYLILIMC   293
MC4R-Mouse          AITLTILIGVFVVCWAPFFLHLLFYISCPQNPYCVCFMSHFNLYLILIMC   293
MC4R-Rat            AITLTILIGVFVVCWAPFFLHLLFYISCPQNPYCVCFMSHFNLYLILIMC   293
MC4R-Opossum        AITLTILIGVFVVCWAPFFLHLIFYISCPQNPYCVCFMSHFNFYLILIMC   293
MC4R-Chicken        AITLTILIGVFVVCWAPFFLHLIFYISCPYNPYCVCFMSHFNFYLILIMC   292
MC4R-Turkey         AITLTILIGVFVVCWAPFFLHLIFYISCPYNPYCVCFMSHFNFYLILIMC   292
MC4R-Zebrafinch     AITLTILIGVFVVCWAPFFLHLIFYISCPYNPYCVCFMSHFNFYLILIMC   292
MC4R-AnoleLizard    AITLTILIGVFVVCWAPLFFHLLFYISCPHNPYCICFMSHFHLYLILIMC   293
MC4R-Takifugu       AITLTILLGVFVVCWAPFFLHLILMITCPKNPYCTCFMSHFNMYLILIMC   260
MC4R-Tetraodon      AITLTILLGVFVVCWAPFFLHLILMITCPKNPYCTCFMSHFNMYLILIMC   290
MC4R-Sickleback     AITLTILLGVFVVCWAPFFLHLILMITCPRNPYCTCFMSHFNMYLILIMC   293
MC4R-Medaka         AITLTILLGVFVVCWAPFFLHLILMITCPRNPYCTCFMSHFNMYLILIMC   288
MC4R-Zebrafish      AITITILLGVFVVCWAPFFLHLILMISCPRNPYCVCFMSHFNMYLILIMC   291
MC5R-Human          AVTVTMLLGVFTVCWAPFFLHLTLMLSCPQNLYCSRFMSHFNMYLILIMC   286
MC5R-Mouse          AITLTMLLGIFIVCWSPFFLHLILMISCPQNVYCSCFMSYFNMYLILIMC   333
MC5R-Rat            AITLTMLLGIFIVCWSPFFLHLILMISCPQNVYCACFMSYFNMYLILIMC   321
MC5R-Opossum        AITLTMLLGVFIVCWAPFFLHLILMISCPQNLYCICFMSHFNMYLILIMC   286
MC5R-Chicken        AITLTMLLGIFIVCWAPFFLHLILMISCPQNLYCVCFMSHFNMYLILIMC   286
MC5R-Zebrafinch     AITLTMLLGIFIVCWAPFFLHLILMISCPQNLYCVCFMSHFNMYLILIMC   249
MC5R-Turkey         AITLTMLLGIFIVCWAPFFLHLILMISCPQNLYCVCFMSHFNMYLILIMC   286
MC5R-Takifugu       AITLTILLGIFIICWAPFFLHLILMISCPRNLYCMCFMSHFNMYLILIMC   297
MC5R-Tetraodon      AITLTILLGIFIVCWAPFFLHLILMISCPRNVYCVCFMSHFNMYLILIMC   297
MC5R-Sickleback     AITLTILLGIFIVCWAPFFLHLILMISCPRNLYCVCFMSHFNMYLILIMC   301
MC5R-Medaka         AITLTILLGIFIVCWAPFFLHLILMISCPRNLYCMCFMSHFNMYLILIMC   302
MC5Rb-Zebrafish     AVTLTILLGIFIVCWAPFFLHLILMISCPRNIYCVCFMSHFNMYLILIMC   289
MC5Ra-Zebrafish     AVTLTILLGIFIVCWAPFFLHLILMISCPRNLYCMCFMSHFNMYLILIMC   291
MC5R-Callorhinchus  AITLTILLGIFIICWAPFFLHLILVLACPSNPYCTCYMSLFQVDLILIMC   273
MCAR-Lampetra       AVTLSILLGVFVFCWAPFFLHLTFIISCPANPYCCAYIAYFPLYLLLIMI   292
MCAR_Lamprey        AVTLSILLGVFVFCWAPFFLHLTFIISCPANPYCCAYIAYFPLYLLLIMI   207
MCBR-Lampetra       AITLTILLGVFIVCWAPFFLHLILIVSCPRSPYCVCYMSHFNLYLVLIML   295
MCBR_Lamprey        AITLTILLGVFIVCWAPFFLHLILIVSCPRSPYCVCYMSHFNLYLVLIML   254
MCRL-Branchiostoma  AVTVVMIAAMFIVIWLPICIGVVRGISCHADENCETEARPY-WGLLIAFC   230
```

```
                    TM7
MC1R-Human          NAIIDPLIYAFHSQELRRTLKEVL-TC-SW--------------------   317
MC1R-Mouse          SSTVDPLIYAFRSQELRMTLKEVL-LC-SW--------------------   315
MC1R-Rat            SSIVDPLIYAFRSQELRMTLKEVL-LC-SW--------------------   317
MC1R-Opossum        NSIIDPLIYAFRSQELRKTFKEVI-LCSC----------------------   316
MC1R-Chicken        NSVVDPLIYAFRSQELRRTLREVV-LC-SW--------------------   314
MC1R-Zebrafinch     NSVIDPLIYAFRSQELRRTLREVV-TC-SW--------------------   314
MC1R-AnoleLizard    NSVVDPVIYAFRSQELKRTLKDML-FL-VW--------------------   299
MC1R-Xenopus        NSVIDPLIYAFRSQELRKT-------------------------------   303
MC1R-Takifugu       NSLIDPLIYAYRSQELRKTLQELV-LC-SW--------------------   312
MC1R-Tetraodon      NSLIDPLIYAYRSQELRKTLQELL-LC-SWCLGL----------------   325
MC1R-Medaka         NSLIDPLIYAYRSQELRKTLQEML-MC-SF--------------------   313
MC1R-Sickleback     NSLIDPLIYAYRSHELRRTLRELV-LC-SWCFGV----------------   323
MC1R-Zebrafish      NSLIDPLIYAYRSQELRKTLKELI-FC-SWCFAV----------------   323
MC1R-Callorhinchus  NSVIDPII------------------------------------------   267
MC2R-Human          NAVIDPFIYAFRSPELRDAFKKMIFCSRYW--------------------   297
MC2R-Mouse          NAVIDPFIYAFRSPELRDAFKRML-FCNRY---------------------   296
MC2R-Rat            NAVIDPFIYAFRSPELREAFKKML-FCNRY---------------------   296
MC2R-Opossum        NAIIDPMIYAFRSPELRSTFKKMF-CCSGYK--------------------   312
MC2R-Chicken        NAIIDPMIFAFRSPELRSTFKKMF-CCARYNWNWWKLNEGEYYRSTPMQH   337
MC2R-Zebrafinch     NAIINPMIFAFRSPELRSTFKKMF-YCSRSNCSW-----------------   318
MC2R-Turkey         NAIIDPMIFAFRSPELRSTFKKMF-CCARHNW-------------------   308
MC2R-AnoleLizard    NAVIDPMIYAFRSPELRSTFRRFI-SC-SY--------------------   277
MC2R-Takifugu       HALIDPVIYAFRIPELRHTFRRML-PCLNWR--------------------   312
MC2R-Tetraodon      HALIDPGIYAFRIPELRHTFRRML-LCLNWRLSLT----------------   232
MC2R-Sickleback     HAFIDPAIYAFRSVELRHTFRKML-LCSDWKRCS-----------------   291
MC2R-Medaka         --------------------------------------------------     -
MC2R-Zebrafish      HAVIDPAIYAFRSVELRNTYKKML-LSSASRICKRCA--------------   304
MC3R-Human          NSVIDPLIYAFRSLELRNTFREILCGCNGMNLG-----------------   323
MC3R-Mouse          NSVIDPLIYAFRSLELRNTFKEILCGCNSMNLG-----------------   323
MC3R-Rat            NSVIDPLIYAFRSLELRNTFKEILCGCNGMNVG-----------------   323
MC3R-AnoleLizard    NSVIDPIIYAFRSLEMRKTFKEMVCCCFGLSSG-----------------   289
MC3R-Xenopus        NSIIDPLIYAFRSLEMRKTFKEII-CCYGLDLLIGWGGRY----------   326
MC3R-Zebrafinch     NSVIDPLIYAFRSLEMRKTFKEIMCCCYGMSVG-----------------   295
MC3R-Turkey         NSVIDPLIYAFRSLEMRKTFKEIVCCCYGVSVG-----------------   321
MC4R-Xenopus        NSVIDPLIYALRSQELRKTFKEMM-CCYCMSMGKTFL-------------   327
MC3R-Zebrafish      NSVIDPLIYACRSLEMRKTFKEIL-CCFGCQP---AL-------------   327
MC3R-Callorhinchus  NSVIDPMIYAFRSQEMRKTFKEIA-CCYGMNLNSRCKY------------   322
MC4R-Human          NSIIDPLIYALRSQELRKTFKEII-CCYPLGG---LCD----LSSRY---   332
MC4R-Mouse          NAVIDPLIYALRSQELRKTFKEII-CFYPLGG---ICE----LSSRY---   332
MC4R-Rat            NAVIDPLIYALRSQELRKTFKEII-CFYPLGG---ICE----LPGRY---   332
MC4R-Opossum        NSIIDPLIYALRSQELRKTFKEII-CCYTLGG---PCD----LAGRY---   332
MC4R-Chicken        NSIIDPLIYAFRSQELRKTFKEII-CCCNLRG---LCD----LPGKY---   331
MC4R-Turkey         NSIIDPLIYAFRSQELRKTFKEII-CCCSLRG---LCD----LPGKY---   331
MC4R-Zebrafinch     NSIIDPLIYAFRSQELRKTFKEII-CCCSLRG---LCD----FPGKY---   331
MC4R-AnoleLizard    NSIIDPLIYAFRSQELRKTFKEIM-CCYSLRE---LCD----FSSKY---   332
MC4R-Takifugu       NSVIDPIIYAFRSQEMRKTFKEIF-CCSQMLLMNTCTEFCNGYDSKY---   306
MC4R-Tetraodon      NSVIDPIIYAFRSQEMRKTFKEIF-CCSQALL---CM-------------   323
MC4R-Sickleback     NSVIDPIIYAFRSQEMRKTFREIF-CCSHALL---FV-------------   326
MC4R-Medaka         NSVIDPIIYAFRSQEMRKTFKEIF-CCSNALV---CF-------------   321
MC4R-Zebrafish      NSVIDPLIYAFRSQEMRKTFKEIC-CC--WYGLASLCV------------   326
MC5R-Human          NSVMDPLIYAFRSQEMRKTFKEII-CCRGFRI---ACS----FPRRD---   325
MC5R-Mouse          NSVIDPLIYALRSQEMRRTFKEIV-CCHGFRR---PCR----LLGGY---   372
MC5R-Rat            NSVIDPLIYALRSQEMRRTFKEII-CCHGFRR---TCT----LLGRY---   360
MC5R-Opossum        NSVIDPLIYAFRSQEMRKTFKEII-CCYGLRM---TCG----FPIKY---   325
MC5R-Chicken        NSVIDPLIYAFRSQEMRKTFKEII-CCYSVRM---VCG----LSNKY---   325
MC5R-Zebrafinch     NSVIDPLIYAFRSQEMRKTFKEII-CCYSLR-------------------   279
MC5R-Turkey         NSVIDPLIYAFRSQEMRKTFKEII-CCYSVRT---VCG----LSNKY---   325
MC5R-Takifugu       NSVIDPLIYAFRSQEMRKTFKEII-FCYSLRN---TCSTICTLPGKY---   340
MC5R-Tetraodon      NSVIDPLIYAFRSQEMRKTFKEII-CCYTLRN---ACSSFCTFTGKY---   340
MC5R-Sickleback     NSVIDPLIYAFRSQEMRKTFKEII-CCYSLRN---ACTNICALTGKY---   344
MC5R-Medaka         NAVIDPLIYAFRSQEMRKTFKEII-FCFSLTN---ICTNLCALTGKY---   345
MC5Rb-Zebrafish     NSVIDPLIYALRSQEMRKTFKEIV-CCEGLRS---FCN----MVSKY---   328
MC5Ra-Zebrafish     NSVIDPLIYAFRSQEMRKTLKEII-CCYSLRN---VFG----MSR-----   328
MC5R-Callorhinchus  NSIIDPLIFAFRSPELRNTFKKMC-IC-----------------------   299
MCAR-Lampetra       NSVIDPLIYAFRSPELRVIIRDTLRKCGRGRGRGANGTRGSSCCCVQVR-   341
MCAR_Lamprey        NSVIDPLIYAFRSPELRVIIRDTLRKCGR---------------------   236
MCBR-Lampetra       SSVIDPIIYAFRSHEMRHTFKEIV-CCYSGSL---YCA--LPATWKY---   336
MCBR_Lamprey        SSVIDPIIYAFRSHEMRHTFKEIV-CCYSGSL---Y--------------   286
MCRL-Branchiostoma  GSVVNPVIYAFRMKKIRE--------------------------------   248
```

```
MC1R-Human         --------------------     -
MC1R-Mouse         --------------------     -
MC1R-Rat           --------------------     -
MC1R-Opossum       --------------------     -
MC1R-Chicken       --------------------     -
MC1R-Zebrafinch    --------------------     -
MC1R-AnoleLizard   --------------------     -
MC1R-Xenopus       --------------------     -
MC1R-Takifugu      --------------------     -
MC1R-Tetraodon     --------------------     -
MC1R-Medaka        --------------------     -
MC1R-Sickleback    --------------------     -
MC1R-Zebrafish     --------------------     -
MC1R-Callorhinchus --------------------     -
MC2R-Human         --------------------     -
MC2R-Mouse         --------------------     -
MC2R-Rat           --------------------     -
MC2R-Opossum       --------------------     -
MC2R-Chicken       HFAELKILTQNDTTLAGNCQ   357
MC2R-Zebrafinch    --------------------     -
MC2R-Turkey        --------------------     -
MC2R-AnoleLizard   --------------------     -
MC2R-Takifugu      --------------------     -
MC2R-Tetraodon     --------------------     -
MC2R-Sickleback    --------------------     -
MC2R-Medaka        --------------------     -
MC2R-Zebrafish     --------------------     -
MC3R-Human         --------------------     -
MC3R-Mouse         --------------------     -
MC3R-Rat           --------------------     -
MC3R-AnoleLizard   --------------------     -
MC3R-Xenopus       --------------------     -
MC3R-Zebrafinch    --------------------     -
MC3R-Turkey        --------------------     -
MC4R-Xenopus       --------------------     -
MC3R-Zebrafish     --------------------     -
MC3R-Callorhinchus --------------------     -
MC4R-Human         --------------------     -
MC4R-Mouse         --------------------     -
MC4R-Rat           --------------------     -
MC4R-Opossum       --------------------     -
MC4R-Chicken       --------------------     -
MC4R-Turkey        --------------------     -
MC4R-AnoleLizard   --------------------     -
MC4R-Zebrafinch    --------------------     -
MC4R-Takifugu      --------------------     -
MC4R-Tetraodon     --------------------     -
MC4R-Sickleback    --------------------     -
MC4R-Medaka        --------------------     -
MC4R-Zebrafish     --------------------     -
MC5R-Human         --------------------     -
MC5R-Mouse         --------------------     -
MC5R-Rat           --------------------     -
MC5R-Opossum       --------------------     -
MC5R-Chicken       --------------------     -
MC5R-Zebrafinch    --------------------     -
MC5R-Turkey        --------------------     -
MC5R-Takifugu      --------------------     -
MC5R-Tetraodon     --------------------     -
MC5R-Sickleback    --------------------     -
MC5R-Medaka        --------------------     -
MC5Rb-Zebrafish    --------------------     -
MC5Ra-Zebrafish    --------------------     -
MC5R-Callorhinchus --------------------     -
MCAR-Lampetra      --------------------     -
MCAR_Lamprey       --------------------     -
MCBR-Lampetra      --------------------     -
MCBR_Lamprey       --------------------     -
MCRL-Branchiostoma --------------------     -
```
