# Supplementary figures and images for: Spliceosomal Intron Insertions in Genome Compacted Ray-Finned Fishes as Evident from Phylogeny of MC Receptors, Also Supported by a Few Other GPCRs
Source: PLoS One. 2011 Aug 5;6(8):e22046. doi: 10.1371/journal.pone.0022046 (PMC3151243; doi:10.1371/journal.pone.0022046)

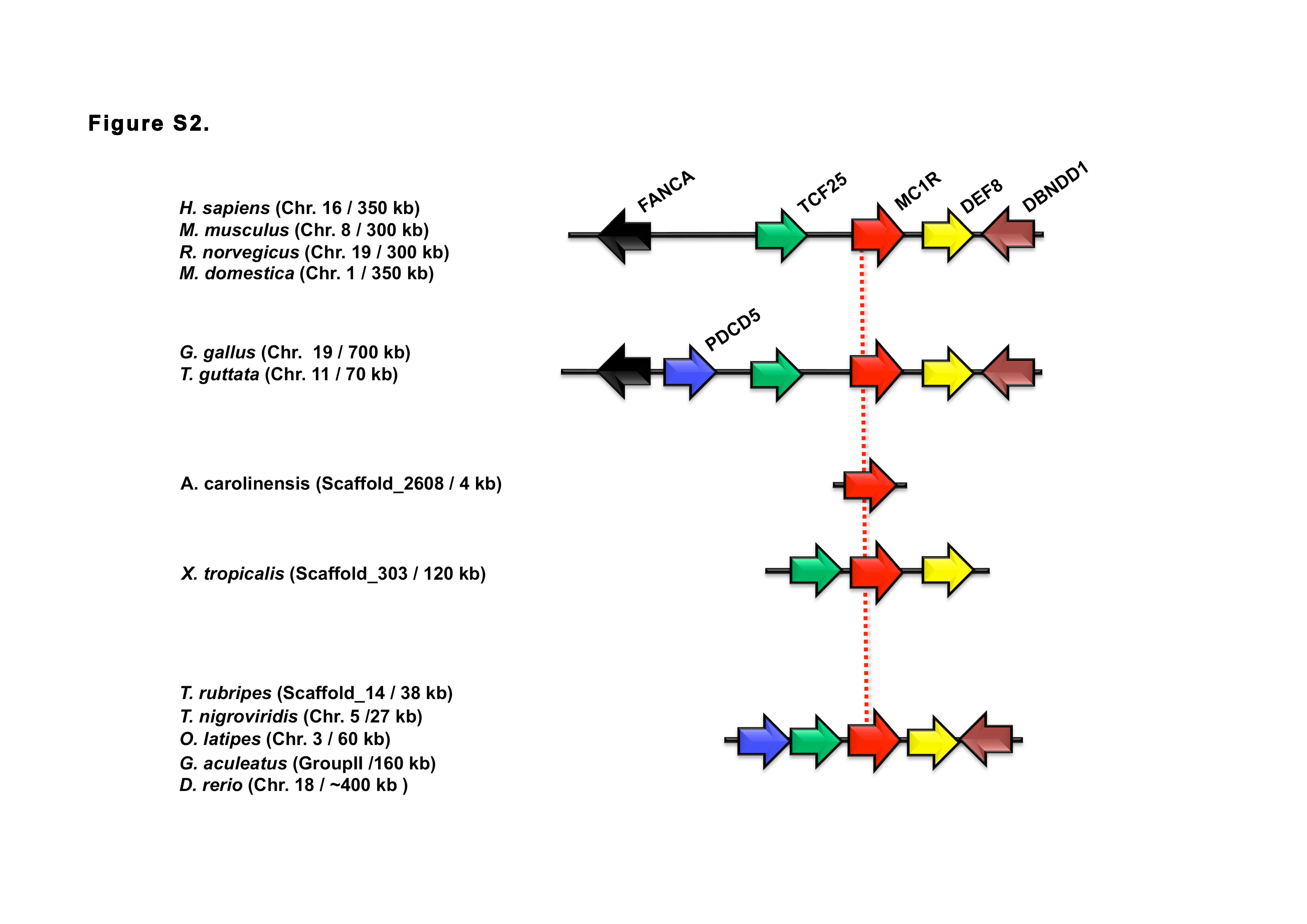

Supplement: Figure S2 — Chromosomal mapping of MC1 receptors. This figure illustrates that MC1R ortholog is found conserved from teleost fishes to mammals. (TIFF) [file pone.0022046.s002.tiff]

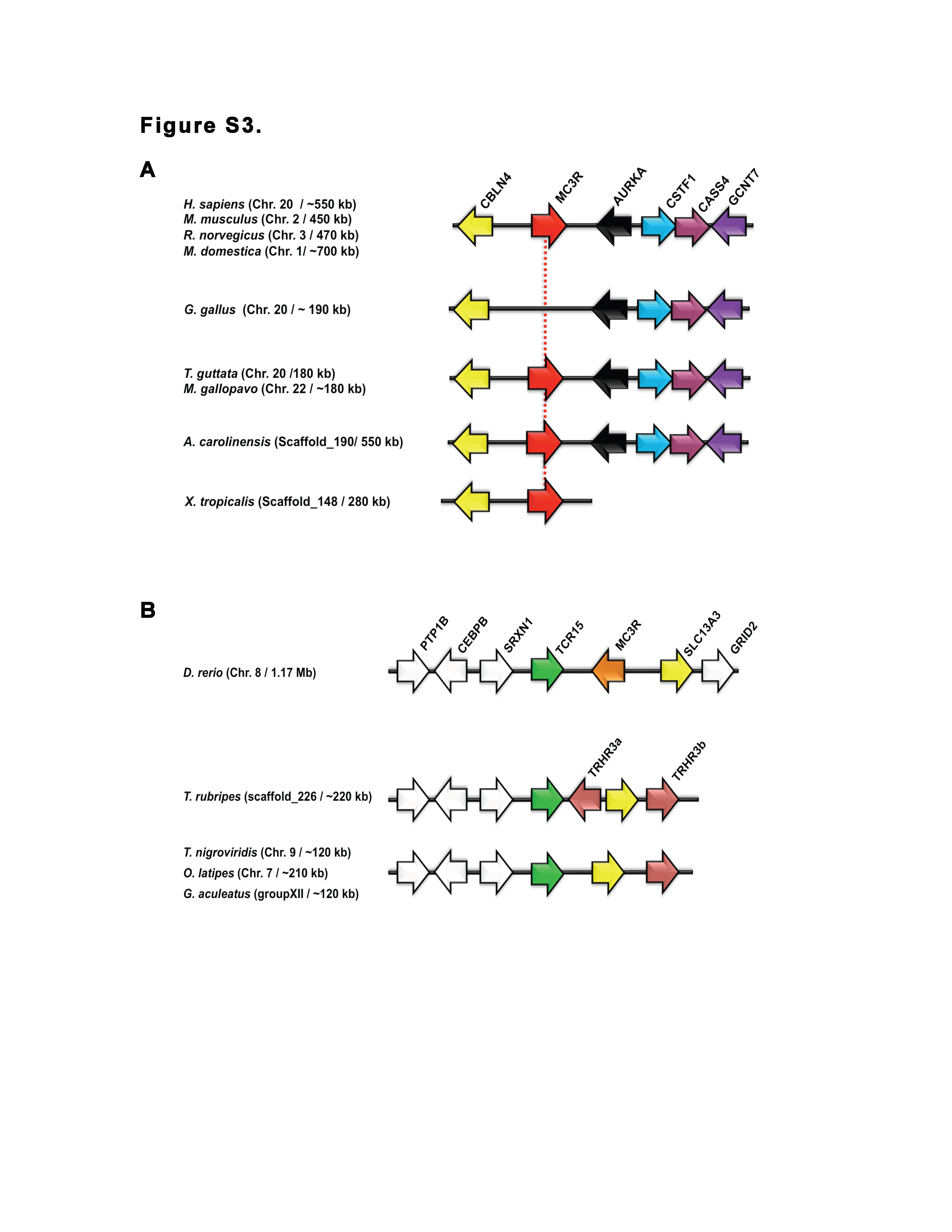

Supplement: Figure S3 — Micro-synteny analysis of MC3 receptors. A. Ortholog of MC3R is conserved in tetrapods. B. MC3R like gene is found in only zebrafish whereas other ray-finned fishes have another thyrotropin-releasing hormone receptor 3 (TRHR3) gene at this locus instead of MC3R. Takifugu has two copies of TRHR3, which are named as TRHR3a-b. (TIFF) [file pone.0022046.s003.tiff]

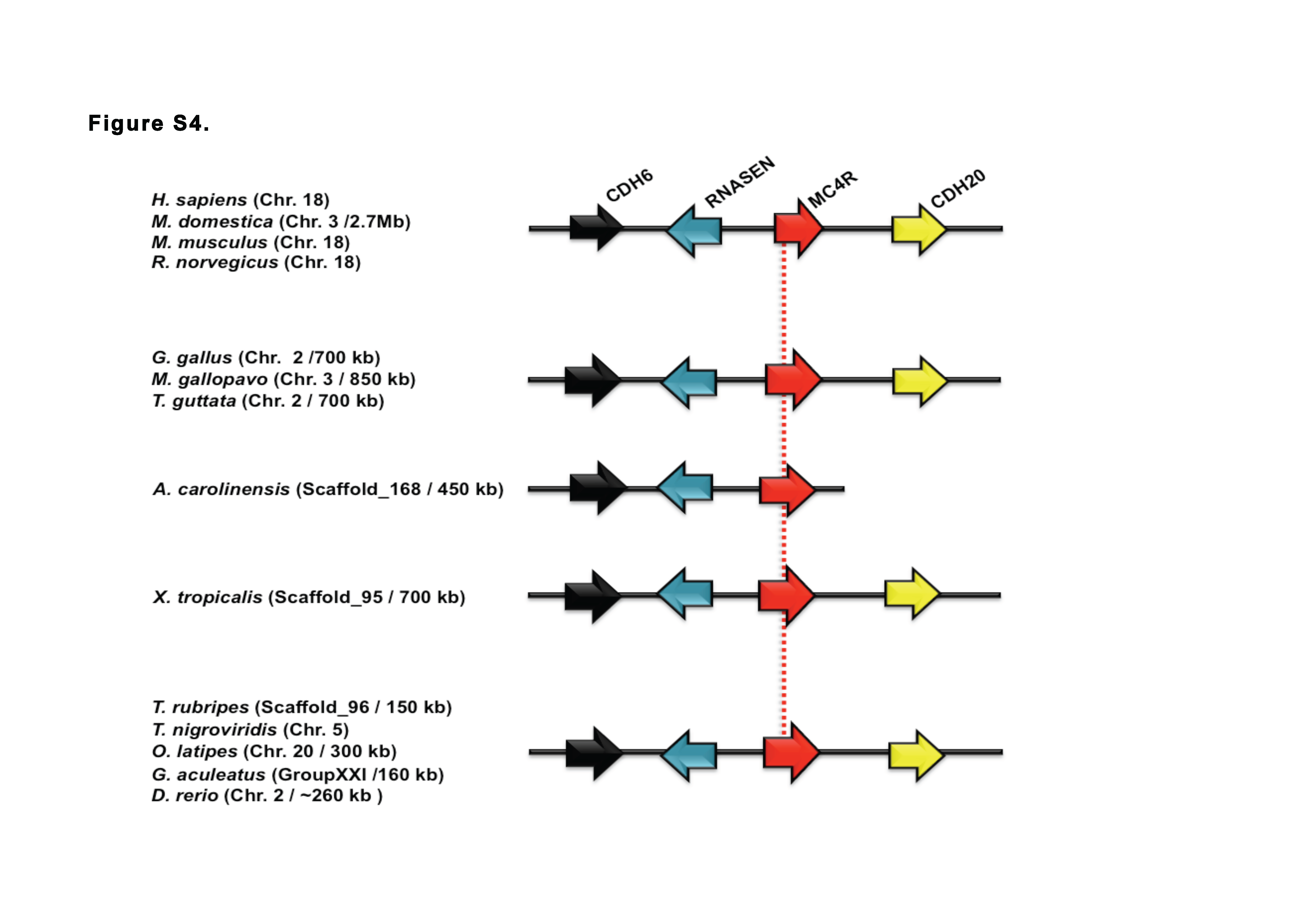

Supplement: Figure S4 — Micro-synteny analysis of MC4R genes depicting presence of orthologs of MC4R gene from fish to mammals. (TIFF) [file pone.0022046.s004.tiff]
