# Supplementary material for: Spliceosomal Intron Insertions in Genome Compacted Ray-Finned Fishes as Evident from Phylogeny of MC Receptors, Also Supported by a Few Other GPCRs
Source: PLoS One. 2011 Aug 5;6(8):e22046. doi: 10.1371/journal.pone.0022046 (PMC3151243; doi:10.1371/journal.pone.0022046)
Supplement: Figure S5 — Intron insertions in MC5 receptor during diversification of ray-finned fishes. There are three intron inserted at positions 41a, 77c and 140c (numbering human MC5R with suffix a–c for intron phasing; blue background) in MC5Rs of four fishes - Takifugu, Tetraodon, stickleback and medaka, but not in MC5R like genes from zebrafish, elephant shark and tetrapods. Transmembrane regions are marked as TM1–TM7 (yellow bars) as predicted by TMHMM2.0 [106]. Residues conserved above 70% are marked by white on black background. In mouse and rat MC5R, there is one intron inserted in N-terminal extension marked as 34c-MMU (numbering according to mouse MC5R; red background). (PDF) [file pone.0022046.s005.pdf]

Figure S5.

```
MC5R-Human          -----------------------------------------------MNS     3
MC5R-Mouse          MQDQSPVNRRFNSQKPPGTREESCLPLRGAEQNGKSDAKKWGHSLPAMNS    50
MC5R-Rat            ------------SQKPPGTREESCLPLRGAEQNGKFEAKKWRHFLPAMNS    38
MC5R-Chicken        --------------------------------MNTS---SQLYVSELNLS    15
MC5R-Turkey         --------------------------------MNTS---SQLYVSELNLS    15
MC5R-Zebrafinch     --------------------------------------------------     -
MC5R-Takifugu       --------------------------------MNTSHRSSDPQEGIMGNS    18
MC5R-Tetraodon      --------------------------------MNATHGSSNPQEGILGNS    18
MC5R-Sickleback     --------------------------------MNLSDESSYREELLLGNF    18
MC5R-Medaka         -----------------------------TAPMEVTDKTNSLHKAQLANS    21
MC5Ra-Zebrafish     --------------------------------MNTS-ETTLPFWGMHVNS    17
MC5Rb-Zebrafish     --------------------------------MNSSEWPTLSPNSSLSQA    18
                                                      34c-MMU
```

```
                                                          TM1
MC5R-Human          SFHLHFLDLNLNATEGNLSGPNVKNKSSPCEDMGIAVEVFLTLGVISLLE    53
MC5R-Mouse          SSTLTVLNLTLNASEDGILGSNVKNKSLACEEMGIAVEVFLTLGLVSLLE   100
MC5R-Rat            SSHLTLLDLTLNASEDNILGQNVNNKSSACEDMGIAVEVFLTLGLVSLLE    88
MC5R-Chicken        AFGS-----NFTVPTV-------KSKSSPCEQVVIAAEVFLTLGIVSLLE    53
MC5R-Turkey         AFGS-----NFTVPTV-------KSKSSPCEQVVIAAEVFLTLGIVSLLE    53
MC5R-Zebrafinch     ---------------------------SPCEQVVIAAEVFLTLGIVSLLE    23
MC5R-Takifugu       TWNPLSYQPNFTLSPPLLP----KTKTAACEQLHIAIEVFLTLGIISLLE    64
MC5R-Tetraodon      TWNSLFHQPNSSLSPPPLP----KNKTAACEQLHIAIEVFLILGIISLLE    64
MC5R-Sickleback     TLAYSYHHQNYTPVPPLHPDKTGTSKPAACEQVHIAIEVFLILGIISLLE    68
MC5R-Medaka         TWN--FYQQNYTIAPPSLKDKTSPSAAAACEQVHIAVEVFLILGIVSLLE    69
MC5Ra-Zebrafish     SPAS--YILNATETP-------SHNKPKACEQLNIATEVFLILGIVSLLE    58
MC5Rb-Zebrafish     NLS------DESSRPK-------TSASAACEQVHIAPEVFLTLGLISLLE    55
                                                     41a
```

```
                                              TM2
MC5R-Human          NILVIGAIVKNKNLHSPMYFFVCSLAVADMLVSMSSAWETITIYLLNNKH   103
MC5R-Mouse          NILVIGAIVKNKNLHSPMYFFVGSLAVADMLVSMSNAWETVTIYLLNNKH   150
MC5R-Rat            NILVIGAIVKNKNLHSPMYFFVGSLAVADMLVSMSNAWETITIYLINNKH   138
MC5R-Chicken        NILVICAIVKNKNLHSPMYFFVCSLAVADMLVSVSNAWETITIYLINNRH   103
MC5R-Turkey         NILVICAIVKNKNLHSPMYFFVCSLAVADMLVSVSNAWETITIYLINNRH   103
MC5R-Zebrafinch     NILVICAIVKNKNLHSPMYFFVCSLAVADMLVSVSNAWETITIYLINNRH    73
MC5R-Takifugu       NILVIMAIVKNKNLHSPMYFFVCSLAVADMLVSVSNASETIIIYLLNNKQ   114
MC5R-Tetraodon      NILVITAIVKNKNLHSPMYFFVCSLAVADMLVSVSNAWETIIIYLLNNKQ   114
MC5R-Sickleback     NILVITAIVKNKNLHSPMYFFVCSLAVADMLVSVSNAWETIVIYLLNNRQ   118
MC5R-Medaka         NILVITAIVKNKNLHSPMYFFVCSLAVADMLVSVSNAWETIIIYLLNNRQ   119
MC5Ra-Zebrafish     NILVICAIVKNKNLHSPMYFFVCSLAVADMLVSVSNAWETIVIYLLTNRQ   108
MC5Rb-Zebrafish     NILVILAIVKNKNLHSPMYFFVCSLAVADMLVSVSNAWETIVIHLLANRS   105
                                          77c
```

```
                                        TM3
MC5R-Human          LVIADAFVRHIDNVFDSMICISVVASMCSLLAIAVDRYVTIFYALRYHHI   153
MC5R-Mouse          LVIADTFVRHIDNVFDSMICISVVASMCSLLAIAVDRYITIFYALRYHHI   200
MC5R-Rat            VVIADTFVRHIDNVFDSMICISVVASMCSLLAIAVDRYITIFYALRYHHI   188
MC5R-Chicken        IIMEDAFVRHIDNVFDSLICISVVASMCSLLAIAVDRYITIFYALRYHNI   153
MC5R-Turkey         IIMEDAFVRHIDNVFDSLICISVVASMCSLLAIAIDRYITIFYALRYHNI   153
MC5R-Zebrafinch     VIMEDAFVRHIDNVFDSMICISVVASMCSLLAIAVDRYITIFYALRYHNI   123
MC5R-Takifugu       LIAEDHLIRQLDNVFDSMICISVVASMCSLLAIAVDRYVTIFYALRYHNI   164
MC5R-Tetraodon      LVVEDHFIQQLDNVFDSMICISVVASMCSLLAIAVDRYVTIFYALRYHNI   164
MC5R-Sickleback     LVVEDHFIRQMDNVFDSMICISVVASMCSLLAIAVDRYVTIFYALRYHNI   168
MC5R-Medaka         LVVEEHFIRQLDNIFDSMICISVVASMCSLLAIAVDRYVTIFYALRYHNI   169
MC5Ra-Zebrafish     LVVEDHFIRQMDNVFDSMICISVVASMCSLLAIAVDRYVTIFYALRYHNI   158
MC5Rb-Zebrafish     LVIEDHFIRQMDNVFDSLICISVVGSMWSLLAIAVDRYVTIFYALRYHNI   155
                                                    140c
```

(Numbering according to human MC5R)

```
                      TM4                       TM5
MC5R-Human        MTARRSGAIIAGIWAFCTGCGIVFILYSESTYVILCLISMFFAMLFLLVS   203
MC5R-Mouse        MTARRSGVIIACIWTFCISCGIVFIIYYESKYVIICLISMFFTMLFFMVS   250
MC5R-Rat          MTARRSGVIIACIWTFCISCGIVFIIYYESKYVIVCLISMFFTMLFFMVS   238
MC5R-Chicken      MTVKRSGLIIACIWTFCTGCGIIFILYYESTYVIICLITMFFTMLFLMVS   203
MC5R-Turkey       MTVKRSGLIIACIWTFCTGCGIIFILYYESTYVIICLITMFFTMLFLMVS   203
MC5R-Zebrafinch   MTVKRSGLIIACIWTFCTGCGIIFILYYESTYVVICLITMFFTMLFLMVS   173
MC5R-Takifugu     MTVRRAGCIIGGIWTFCTGCGIVFIIYSDTTPVIICLVCMFFAMLLIMAS   214
MC5R-Tetraodon    MTVRRAGCIIGGIWTFCTGCGIVFIIYSEKTPVIICLVSMFFAMLLIMAS   214
MC5R-Sickleback   MTVRRAGCIIGGIWTFCTGCGIIFIIYSDTTPVIICLVSMFFAMLVIMAS   218
MC5R-Medaka       MTMRRAGCIIGGIWIFCTGCGIVFILYSETTPVIICLVSMFFAMLLIMAS   219
MC5Ra-Zebrafish   MTVRRAALIIGGIWTFCTGCGIVFIIYSDNTSVIVCLVSMFFIMLALMAS   208
MC5Rb-Zebrafish   MTVRRAGILIGSIWTFSTSCGIIFIIYSDTQPVVVCLVAMFFAMLLMMAS   205

                                                    TM6
MC5R-Human        LYIHMFLLARTHVKRIAALPGASS-ARQRTSMQGAVTVTMLLGVFTVCWA   252
MC5R-Mouse        LYIHMFLLARNHVKRIAASPRYNS-VRQRTSMKGAITLTMLLGIFIVCWS   299
MC5R-Rat          LYIHMFLLARNHVKRIAASPRYNS-VRQRASMKGAITLTMLLGIFIVCWS   287
MC5R-Chicken      LYIHMFLLARTHVKKIAALPGYNS-VHQRTSMKGAITLTMLLGIFIVCWA   252
MC5R-Turkey       LYIHMFLLARTHVKKIAALPGYNS-VHQRTSMKGAITLTMLLGIFIVCWA   252
MC5R-Zebrafinch   LYIHMFLLARTHVKKIAALPG--------TSMKGAITLTMLLGIFIVCWA   215
MC5R-Takifugu     LYSHMFMLARSHVKRIAALPGSNS-IHQRASMKGAITLTILLGIFIICWA   263
MC5R-Tetraodon    LYSHMFMLARSHVKRIAALPGSSS-IQQRANMKGAITLTILLGIFIVCWA   263
MC5R-Sickleback   LYSHMFMLARSHVKRIAALPGYNS-IHQRTSMKGAITLTILLGIFIVCWA   267
MC5R-Medaka       LYSHMFMLARSHVKRIAALPGYNS-IHQRASMKGAITLTILLGIFIVCWA   268
MC5Ra-Zebrafish   LYSHMFMLARSHVKRIAALPGYNS-IHQRASMKAAVTLTILLGIFIVCWA   257
MC5Rb-Zebrafish   LYSHMFLLARSHVKRMAALPGYNANIRQRASMKGAVTLTILLGIFIVCWA   255

                                          TM7
MC5R-Human        PFFLHLTLMLSCPQNLYCSRFMSHFNMYLILIMCNSVMDPLIYAFRSQEM   302
MC5R-Mouse        PFFLHLILMISCPQNVYCSCFMSYFNMYLILIMCNSVIDPLIYALRSQEM   349
MC5R-Rat          PFFLHLILMISCPQNVYCACFMSYFNMYLILIMCNSVIDPLIYALRSQEM   337
MC5R-Chicken      PFFLHLILMISCPQNLYCVCFMSHFNMYLILIMCNSVIDPLIYAFRSQEM   302
MC5R-Turkey       PFFLHLILMISCPQNLYCVCFMSHFNMYLILIMCNSVIDPLIYAFRSQEM   302
MC5R-Zebrafinch   PFFLHLILMISCPQNLYCVCFMSHFNMYLILIMCNSVIDPLIYAFRSQEM   265
MC5R-Takifugu     PFFLHLILMISCPRNLYCMCFMSHFNMYLILIMCNSVIDPLIYAFRSQEM   313
MC5R-Tetraodon    PFFLHLILMISCPRNVYCVCFMSHFNMYLILIMCNSVIDPLIYAFRSQEM   313
MC5R-Sickleback   PFFLHLILMISCPRNLYCVCFMSHFNMYLILIMCNSVIDPLIYAFRSQEM   317
MC5R-Medaka       PFFLHLILMISCPRNLYCMCFMSHFNMYLILIMCNAVIDPLIYAFRSQEM   318
MC5Ra-Zebrafish   PFFLHLILMISCPRNLYCMCFMSHFNMYLILIMCNSVIDPLIYAFRSQEM   307
MC5Rb-Zebrafish   PFFLHLILMISCPRNIYCVCFMSHFNMYLILIMCNSVIDPLIYALRSQEM   305

MC5R-Human        RKTFKEIICCRGFRIACS----FPRRD   325
MC5R-Mouse        RRTFKEIVCCHGFRRPCR----LLGGY   372
MC5R-Rat          RRTFKEIICCHGFRRTCT----LLGRY   360
MC5R-Chicken      RKTFKEIICCYSVRMVCG----LSNKY   325
MC5R-Turkey       RKTFKEIICCYSVRTVCG----LSNKY   325
MC5R-Zebrafinch   RKTFKEIICCYSLR-------------   279
MC5R-Takifugu     RKTFKEIIFCYSLRNTCSTICTLPGKY   340
MC5R-Tetraodon    RKTFKEIICCYTLRNACSSFCTFTGKY   340
MC5R-Sickleback   RKTFKEIICCYSLRNACTNICALTGKY   344
MC5R-Medaka       RKTFKEIIFCFSLTNICTNLCALTGKY   345
MC5Ra-Zebrafish   RKTLKEIICCYSLRNVFG----MSR--   328
MC5Rb-Zebrafish   RKTFKEIVCCEGLRSFCN----MVSKY   328
```
