# Supplementary material for: Spliceosomal Intron Insertions in Genome Compacted Ray-Finned Fishes as Evident from Phylogeny of MC Receptors, Also Supported by a Few Other GPCRs
Source: PLoS One. 2011 Aug 5;6(8):e22046. doi: 10.1371/journal.pone.0022046 (PMC3151243; doi:10.1371/journal.pone.0022046)
Supplement: Figure S6 — Analysis of intron insertions in MC2R from selected ray-finned fishes. There is one intron inserted at position 140c (numbering human MC5R with suffix a–c for intron phasing; blue background) in MC2Rs of four fishes - Takifugu, Tetraodon, stickleback and medaka, but not in zebrafish and tetrapods. This intron is also conserved in MC5Rs of these selected ray-finned fishes. Takifugu and stickleback has one intron inserted in their MC2R at intron positions 230c (or 225c-TRU, according to Takifugu MC2R numbering) and 236a, respectively. Transmembrane regions are marked as TM1–TM7 (yellow bars) as predicted by TMHMM2.0 [106]. Residues conserved above 70% are marked by white on black background. (PDF) [file pone.0022046.s006.pdf]

Figure S6.

```
MC2R-Human         -----------MKHI----INSYE-NIN--NTARNNSDCPRVVLPEEIFF   32
MC2R-Mouse         -----------MKHI----INSYE-HTN--DTARNNSDCPDVVLPEEIFF   32
MC2R-Rat           -----------MKHI----INSYE-HTN--NTARNNSDCPDVVVPEEIFF   32
MC2R-Opossum       --ERASELIKILGHN----GNPPE-NIT--DNATNNTDCIQVVVPEEVFF   41
MC2R-Chicken       MSTEKPFNLILSAHAGQTSIPSLE-NIT--DFSLNITDCNQVVVPEEVFF   47
MC2R-Turkey        -----------IKHAGQTSIPSLE-NIT--DFSLNITDCNQVVVPEEVFF   36
MC2R-Zebrafinch    MSTERPS--ILIKHPGQTSIPSLE-NIS--DFSLNITDCTQVVVPEEVFF   45
MC2R-AnoleLizard   -------------------------------------------VPEEIFF    7
MC2R-Takifugu      ---------SSKLQVLKFTSLAMN------ATTVNRSDCPEVNVPIHVFF   35
MC2R-Tetraodon     --------------------------------------------------    -
MC2R-Sickleback    ----------------------MD------TAAANRSDCPEVRVPVPLFF   22
MC2R-Medaka        -----------------SNFEVMN------TNSVNRTDCPEVKVPFLIFF   27
MC2R-Zebrafish     ------------------MNPSAE------SPSSIHTDCAEVQVPGQVFL   26
MC5R-Human         ------MNSSFHLHFLDLNLNATEGNLSGPNVKNKSSPCEDMGIAVEVFL   44

                       TM1                         TM2
MC2R-Human         TISIVGVLENLIVLLAVFKNKNLQAPMYFFICSLAISDMLGSLYKILENI   82
MC2R-Mouse         TISVIGILENLIVLLAVIKNKNLQSPMYFFICSLAISDMLGSLYKILENI   82
MC2R-Rat           TISIIGVLENLIVLLAVIKNKNLQSPVYFFICSLAISDMLGSLYKILENI   82
MC2R-Opossum       AISIIGVLENLLVLLAVIKNRNLHSPMYFFICSLAVSDMLGSLYKILENI   91
MC2R-Chicken       TVAAAGILENLLVLVAVIRNKNLHLPMYFFICSLAISDMLGSLYKTLENI   97
MC2R-Turkey        TVAAAGILENLLVLVAVIRNKNLHLPMYFFICSLAISDMLGSLYKTLENI   86
MC2R-Zebrafinch    TVAAAGILENLLILIAVVRNKNLHLPMYFFICSLAISDMLGSLYKTLENI   95
MC2R-AnoleLizard   IIATLGLLENLLVLIAVGRNKNLHSPMYIFICSLAVSDMLGSLYKAVENI   57
MC2R-Takifugu      TIGFVSLLENLLVIGAISWNRNLHSPMYCFIGSLAAFNTVASVTKTWENL   85
MC2R-Tetraodon     -------------------------------------------------V    1
MC2R-Sickleback    TIGVVSLAENLLVVVAVIRNRNLHSPMYCFICSLAAFNTIASLTKTWENL   72
MC2R-Medaka        TVGIVSLAENLLVVLAVILNRNLHSPMYCFICSLAAFNTIASLTKTWENL   77
MC2R-Zebrafish     VIAVASLSENLLVIVAVIKNKNLHSPMYCFICNLAVFNTISSFSKALENI   76
MC5R-Human         TLGVISLLENILVIGAIVKNKNLHSPMYFFVCSLAVADMLVSMSSAWETI   94

                                        TM3
MC2R-Human         L-IILRNMGYLKPRGSFETTADDIIDSLFVLSLLGSIFSLSVIAADRYIT  131
MC2R-Mouse         L-IMFRNMGYLKPRGSFESTADDIIDCMFILSLLGSIFSLSVIAADRYIT  131
MC2R-Rat           L-IMFRNMGYLKPRGSLESTADDIIDCMFVLSLLGSIFSLSVIAADRYIT  131
MC2R-Opossum       L-IIFRNTGYLKPRGDFETTADDVVDSLFILSLLGSIFSLSVIAADRYIT  140
MC2R-Chicken       F-IILCKMGYLTRRGDFEKKLDDAMDSMFILSLLGSIFSLLAIAADRYIT  146
MC2R-Turkey        F-IILCKMGYLTRRGDFEKKLDDAMDSMFILSLLGSIFSLLAIAADRYIT  135
MC2R-Zebrafinch    F-IILCKMGYLTRHGDFEKKLDDAMDSMFILSLLGSIFSLLAIAADRYIT  144
MC2R-AnoleLizard   FYIIFCKMQYVKCRGKLAKTMDDILDFMFILSLLGSIFSLSAIAADRYIT  107
MC2R-Takifugu      M-ITFAEVGHLRKVGFSERKADDVVDSLLCMSFLGSIFSFLAIAVDRYIT  134
MC2R-Tetraodon     M-IMFADVGHLRKVGYLELKLDDVVDSLLCMSFLGSIFSFLAIAGDRYIT   50
MC2R-Sickleback    M-IVFANIGLLEKKGPSETNVDDVLDSLLCMSFVGSIVSFLAIAADRYIS  121
MC2R-Medaka        M-IVFADVGQLEKRGPSETKLDDMMDSLLCMSFVGSIFSFLAIAVDRYIT  126
MC2R-Zebrafish     L-LLFKDAGRLNSRGPFELKIDDIMDSLLCMCFLGSIFSILAIAVDRYIS  125
MC5R-Human         T-IYLLNNKHLVIADAFVRHIDNVFDSMICISVVASMCSLLAIAVDRYVT  143
                                                                  140c
```

(Numbering according to human MC5R)

```
                                       TM4
MC2R-Human          IFHALRYHSIVTMRRTVVVLTVIWTFCTGTGITMVIFSHHVPTVITFTSL   181
MC2R-Mouse          IFHALQYHSIVTMRRTIITLTIIWMFCTGSGITMVIFSHHIPTVLTFTSL   181
MC2R-Rat            IFHALQYHSIVTMRRTVITLTVIWMFCTGSGITMVIFSHHIPTVLTFTSL   181
MC2R-Opossum        IFHALQYHNIMTMRRAISILAIIWAFCTGSGITMVIFSHDVPTVISFTSL   190
MC2R-Chicken        IFYALRYHNIMTLQRALVILAIIWTFCAGSSIAIALFSHEVATVIPFTIL   196
MC2R-Turkey         IFYALRYHNIMTVRRALVILAIIWTFCAGSSIAIALFSHEIATVIPFTIL   185
MC2R-Zebrafinch     IFYALRYHNIMTLRRALVILAIIWAFCAGSSIAIALFSYEAATVIPFTIL   194
MC2R-AnoleLizard    IFYALRYHNIMTLKRALVMLGVIWAFCTGSGIAMVLFSYEAVTVVSFTVL   157
MC2R-Takifugu       IFHALRYHNIMTMQRTGAILGLIWTTCGVSAMLMVRFFDSNLIMSCFVVF   184
MC2R-Tetraodon      IFHALRYHDIMTLQRTGAILGVIWTTCGVSAVLMVRFFDSNLIMGCFVVF   100
MC2R-Sickleback     IFHALRYHNIMTMRRTRAVLGVIWTLCGVSAVLMVRFFDSNFIMICFVVF   171
MC2R-Medaka         IFNALRYHNIMTMQRTGGILVVIWTTCGVSAVLMVRFFDFKFIMICFVVF   176
MC2R-Zebrafish      IFHALRYHMLMTMRRVLIILFTIWVLCGTSGALMVGFFEAATVKIFFIVL   175
MC5R-Human          IFYALRYHHIMTARRSGAIIAGIWAFCTGCGIVFILYSESTYVILCLISM   193
```

```
            TM5
MC2R-Human          FPLMLVFILCLYVHMFLLARSHTRKISTLP------------RANMKGAI   219
MC2R-Mouse          FPLMLVFILCLYIHMFLLARSHARKISTLP------------RTNMKGAM   219
MC2R-Rat            FPLMLVFILCLYIHMFLLARSHARKISTLP------------RANMKGAM   219
MC2R-Opossum        FPLMLIFILCLYVHMFLLARSHAKKIISLPSS-RVQP-----RANMKGAI   234
MC2R-Chicken        FPLMMIFILCLYIHMFLLARSHAKKIASLPTS-AVHQ-----RTNMKGAI   240
MC2R-Turkey         FPLMMFFILCLYIHMFLLARSHAKKIASLPTG-AVHQ-----RTNMKGAI   229
MC2R-Zebrafinch     FPLMMFFILCLYIHMFLLARTHAKKIASLPS--TIHH-----RTNMKGAI   237
MC2R-AnoleLizard    FCFMLILILCLYIHMFLLARSHAKKIALMTTS-SVHQ-----GANMKGAI   201
MC2R-Takifugu       FIISLAIIYILYVYMFILARVHARKIAALPNGSGKHQHQRRWGHGMRGIL   234
MC2R-Tetraodon      FIISLVIIYFLYVYMFILSRVHARRIAALPGGSGKHQHLRRWGNGMRGIM   150
MC2R-Sickleback     FVVSLAIICFLYVYMFMLARIHARKIAALPAS-----------NGMRGAL   210
MC2R-Medaka         FIVSLAIICFLYVYMFLLARIHAKSIAALP--------------------   206
MC2R-Zebrafish      FFTALLLILLLYVHMFLLARHHANRIASMPGAQAQHRK-----SGLRGAL   220
MC5R-Human          FFAMLFLLVSLYIHMFLLARTHVKRIAALPGASSARQ-----RTSMQGAV   238
                                                          230c    236a
                                                       225c-TRU
```

```
              TM6                            TM7
MC2R-Human          TLTILLGVFIFCWAPFVLHVLLMTFCPSNPYCACYMSLFQVNGMLIMCNA   269
MC2R-Mouse          TLTILLGVFIFCWAPFVLHVLLMTFCPNNPYCVCYMSLFQVNGMLIMCNA   269
MC2R-Rat            TLTILLGVFIFCWAPFVLHVLLMTFCPNNPYCVCYMSLFQVNGMLIMCNA   269
MC2R-Opossum        TLTILLGVFLCCWAPFVLHILLVTFCPNNPYCACYLSIFQVNGMLIMCNA   284
MC2R-Chicken        TLTIFLGVFLCCWAPFVLHILLARFCPHNPYCACYMSIFHVNGTLIMCNA   290
MC2R-Turkey         TLTIFLGVFLCCWAPFVLHILLARFCPHNPYCACYMSIFHVNGTLIMCNA   279
MC2R-Zebrafinch     TLTIFLGVFLCCWAPFVLHILLARFCPHNPYCACYMSIFHVNGTLIMCNA   287
MC2R-AnoleLizard    TLTVLVAVFLFCWSPFVLHMLLMTFCPQNPYCICYGSIFHVHGMLIMCNA   251
MC2R-Takifugu       TLTILFGAFMVCWAPFFLHLIFLMACPMNPYCECYRSMFQLHLVLLMSHA   284
MC2R-Tetraodon      TLTILFGAFMVCWAPFFLHLIILMVCPMNPYCECYRSLFELHVVLLMSHA   200
MC2R-Sickleback     TLTILFGAFVVCWAPFFLHLIIIMLCPTNPYCECYRSLFQLHVVLLMSHA   260
MC2R-Medaka         --------------------------------------------------     -
MC2R-Zebrafish      TLTILIGVFVACWAPFSLHLLIMMICPENPYCECYRSLFQLHVVLLVSHA   270
MC5R-Human          TVTMLLGVFTVCWAPFFLHLTLMLSCPQNLYCSRFMSHFNMYLILIMCNS   288
```

```
MC2R-Human        VIDPFIYAFRSPELRDAFKKMIFCSRYW-----------------------  297
MC2R-Mouse        VIDPFIYAFRSPELRDAFKRMLFCNRY------------------------  296
MC2R-Rat          VIDPFIYAFRSPELREAFKKMLFCNRY------------------------  296
MC2R-Opossum      IIDPMIYAFRSPELRSTFKKMFCCSGYK-----------------------  312
MC2R-Chicken      IIDPMIFAFRSPELRSTFKKMFCCARYNWNWWKLNEGEYYRSTPMQHHFA  340
MC2R-Turkey       IIDPMIFAFRSPELRSTFKKMFCCARHNW----------------------  308
MC2R-Zebrafinch   IINPMIFAFRSPELRSTFKKMFYCSRSNCSW--------------------  318
MC2R-AnoleLizard  VIDPMIYAFRSPELRSTFRRFISCSY-------------------------  277
MC2R-Takifugu     LIDPVIYAFRIPELRHTFRRMLPCLNWR-----------------------  312
MC2R-Tetraodon    LIDPGIYAFRIPELRHTFRRMLLCLNWRLSLT-------------------  232
MC2R-Sickleback   FIDPAIYAFRSVELRHTFRKMLLCSDWKRCS--------------------  291
MC2R-Medaka       --------------------------------------------------    -
MC2R-Zebrafish    VIDPAIYAFRSVELRNTYKKMLLSSASRICKRCA-----------------  304
MC5R-Human        VMDPLIYAFRSQEMRKTFKEIICCRGFRIACSFPRRD--------------  325


MC2R-Human        -----------------    -
MC2R-Mouse        -----------------    -
MC2R-Rat          -----------------    -
MC2R-Opossum      -----------------    -
MC2R-Chicken      ELKILTQNDTTLAGNCQ  357
MC2R-Turkey       -----------------    -
MC2R-Zebrafinch   -----------------    -
MC2R-AnoleLizard  -----------------    -
MC2R-Takifugu     -----------------    -
MC2R-Tetraodon    -----------------    -
MC2R-Sickleback   -----------------    -
MC2R-Medaka       -----------------    -
MC2R-Zebrafish    -----------------    -
MC5R-Human        -----------------    -
```
