# Supplementary material for: Spliceosomal Intron Insertions in Genome Compacted Ray-Finned Fishes as Evident from Phylogeny of MC Receptors, Also Supported by a Few Other GPCRs
Source: PLoS One. 2011 Aug 5;6(8):e22046. doi: 10.1371/journal.pone.0022046 (PMC3151243; doi:10.1371/journal.pone.0022046)
Supplement: Figure S7 — Intron insertions in P2Y2 receptor during diversification of ray-finned fishes. There are three introns inserted at positions 124a, 181c and 267a (human MC5R amino acid numbering with suffix a–c for intron phasing) in P2Y2 receptor of four fishes - Takifugu, Tetraodon, stickleback and medaka (blue background), but not in P2Y2 genes from zebrafish, elephant shark and tetrapods. Residues conserved above 70% are marked by white on black background. ### indicates location of highly conserved DRY motif. Transmembrane regions are marked as TM1–TM7 (yellow bars) as predicted by TMHMM2.0 [106]. (PDF) [file pone.0022046.s007.pdf]

Figure S7.

```
MC5R-Human          1  -------------MNSSFHLHFLDLNLNATEGNLSGPNVK-NKSSPCEDM   36
P2Y2-Human          1  --------------MAADLGPWNDTINGTWDGDELGYRCRFNEDFKYVLL   36
P2Y2-Takifugu       1  -------------------------------INP--LYCKFQEDFKYILL   17
P2Y2-Tetraodon      1  -----------------------MVALDNATVDP--LYCKFKEDFKYILL   25
P2Y2-Medaka         1  RALPSAVNLVNLASAGVSTSNMNASGLNLTENNSRSDFCRFNEDFKFILL   50
P2Y2-1-Stickleback  1  -------------IFITKMATWDNNVTSQTNYSN--FYCRFEERFKYILL   35
P2Y2-2-Stickleback  1  ---------------------WDNNVTSQTNYSN--FYCRFEERFKYILL   27
P2Y2-1-Danio        1  ------------------MATFNNTTIA-NASDL--YSCIFDETFKYILL   29
P2Y2-2-Danio        1  ------------------MAAFNNTGPT-NGSNE--TDCDLQDDFKHILL   29
P2Y2-3-Danio        1  ----MICFLCLNLRQIWIRMAMNNTTVSTNDIDI--YRCTLKEDFKYILL   44
P2Y2-Callorhinchus  1  ------------------------------------YNCTFNEEFKYILL   14

                                   TM1                           TM2
MC5R-Human         37  GIAVEVFLTLGVISLLENILVIGAIVKNKNLHSPMYFFVCSLAVADMLVS   86
P2Y2-Human         37  PVSYGVVCVPG---LCLNAVALYIFLCRLKTWNASTTYMFHLAVSDALYA   83
P2Y2-Takifugu      18  PVSYALVFVFG---LALNGTALYVIVFRTRTWKPSTIYMFNLTMCDTLYI   64
P2Y2-Tetraodon     26  PVSYALVFVFG---LALNGLALYVIVFRTKAWKPSTVYMFNLTMCDTLYI   72
P2Y2-Medaka        51  PVSYTLVFVVG---LALNFTALFVMVFRTKHWKPSTIYMFNLTMCDTLYI   97
P2Y2-1-Stickleback 36  PVSYSLVFVIG---LALNATALYVMLFRTKRWKSSTIYMFNLTVCDTLYV   82
P2Y2-2-Stickleback 28  PVSYSLVFVIG---LALNATALYVMLFRTKRWKSSTIYMFNLTVCDTLYV   74
P2Y2-1-Danio       30  PVSYSLVFVFG---LGLNITAMYVILFRTKQWKPNTIYMINLNACDTLYI   76
P2Y2-2-Danio       30  PVSYSLVFVFG---LGLNITAMYFIFFRTKQWKPNTIYIINLSICDTLYI   76
P2Y2-3-Danio       45  PVSYSLVFVFG---LGLNITAMYFILFQTKHWKPNTIYMINLNACDTLYI   91
P2Y2-Callorhinchus 15  PVSYGIVFVVG---LVLNCLAIWVFVFRMRPWNTTTTYMFNLAISDTMYV   61

                                                         124a
                                                               TM3
MC5R-Human         87  MSSAWETITIYLLNNKHLVIADAFVRHIDNVFDSMICISVVASMCSLLAI  136
P2Y2-Human         84  ASLPL--LVYYYARGDHWPFSTVLCKLVRFLFYTNLYCSILFLTC----I  127
P2Y2-Takifugu      65  LTLPF--LIYYYADENDWPFSEPLCKIIRFLFYANLYGSILFLCC----I  108
P2Y2-Tetraodon     73  LTLPF--LIYYYADENDWPFSEPFCKIIRFLFYANLYGSILFLCC----I  116
P2Y2-Medaka        98  FTLPF--LIFYYADENDWPFTEPLCKMIRFLFYSNLYGSILFMCC----I  141
P2Y2-1-Stickleback 83  LTLPF--LIYYYADENDWPFSEPLCKIIRFLFYTNLYGSIMFLCC----I  126
P2Y2-2-Stickleback 75  LTLPF--LIYYYADENDWPFSEPLCKIIRFLFYTNLYGSIMFLCC----I  118
P2Y2-1-Danio       77  LTLPF--LIYYYADENAWPFGELMCKLIRFLFYTNLYGSILFLSC----I  120
P2Y2-2-Danio       77  LTLPF--LIYYYADESDWPFGEAMCKFIRFLFYTNLYGSILFLSC----I  120
P2Y2-3-Danio       92  LTLPF--LIYYYAGANAWPFGDPMCRFIRFFFYTNLYGSILFLSC----I  135
P2Y2-Callorhinchus 62  ISLPL--LVYYYARRNNWPFGAALCKIVRFLFYTNLYCSILFLTC----M  105

                                                                   181c
                         ###                         TM4
MC5R-Human         137 AVDRYVTIFYALRYHHIMTARRSGAIIAGIWAF---CTGCGIVFILYSES  183
P2Y2-Human         128 SVHRCLGVLRPLRSLRWGRARYARRVAGAVWVLVLACQAPVLYFVTTSAR  177
P2Y2-Takifugu      109 SLHRFIGICYPVRSLYWLSTRRAKFISAAVWGVVLVCQGPVLYFSRIRDK  158
P2Y2-Tetraodon     117 SLHRFIGVCYPVRSLYWLSARRAKFISVAVWAVVLSCQSPVLYFSRIRDK  166
P2Y2-Medaka        142 SLHRFVGICFPVRSLSWVSTRRARLVSVAVWASVLFCQGPVLYFSRTRST  191
P2Y2-1-Stickleback 127 SLHRFIGVCYPVRSLSWVSARRAKLVSVAVWACVLLCQAPILYFSRTSKS  176
P2Y2-2-Stickleback 119 SLHRFIGVCYPVRSLSWVSARRAKLVSVAVWACVLLCQAPILFFTRTSVR  168
P2Y2-1-Danio       121 SVHRFIGVCHPVRSLSWTNARRARFISLCIWATMLIFQAPILYFSRMKSD  170
P2Y2-2-Danio       121 SVHRFIGVCHPVRSLSLLNTQYARLVSVGIWVIILILQAPILFFSRTTQK  170
P2Y2-3-Danio       136 SVHRFIGVCHPVRSLSWMNARRARWISVGIWLIILILQTPLLYFSRTRLN  185
P2Y2-Callorhinchus 106 SIHRFLGVCFPMESLRWVKVRNTRIVCAVVWVIVIMCQAPILAFVTTEDK  155
```

```
                                                    TM5
MC5R-Human            184   TYV-ILC--------LISMFF----AMLFLLVSLYIHMFLLARTHVKRIAA   221
P2Y2-Human            178   GGR-VTCHDTSAPELFSRFVAYSSVMLGLLFAVPFAVILVCYVLMARRLL   226
P2Y2-Takifugu         159   NSS-RTCYDTTSPELFDDFLVYSSVISVLMFALPFMVVLVCNGLMVRKLL   207
P2Y2-Tetraodon        167   NSS-RICYDTTSPELFDDFLVYSSVISVLMFALPFMIVMVCNGLMVRKLL   215
P2Y2-Medaka           192   TTKQIICYDTTSPELFDDFLLYSSVVSVLMFALPFMVLMVCYGLMVRKLL   241
P2Y2-1-Stickleback    177   ESV-RICYDTTSPDLFDDFLLYSSAVSVLMFALPFMVVMVCYGLMVRKLL   225
P2Y2-2-Stickleback    169   EGK-RICYDTTSPDLFNDFLLYSSAVSVLMFALPFMVVMVCYGLMVRKLL   217
P2Y2-1-Danio          171   T---MVCYDTTSKELFNDFLVYSSVVMLLLFVLPFGVVLVCNAMMVKKLR   217
P2Y2-2-Danio          171   KD--IVCHDTTREDLYDDFMVYSSVVMFLLFGLPFVVVLYCNAMMVKKLL   218
P2Y2-3-Danio          186   DEV-LVCHDTTIKELFDDFLVYSSVVMLLLFVLPFGVVLVCNFLIVKKLR   234
P2Y2-Callorhinchus    156   GTN-TLCYDTSSRENFNQFVIYNMVQLVLLFCGPFVIVMVCYWFMTKELL   204

                                                                  TM6
MC5R-Human            222   LPGASS--------ARQRTSMQGAVTVTMLLGVFTVCWAPFFLHLTLMLS   263
P2Y2-Human            227   KPAYGT------SGGLPRAKRKSVRTIAVVLAVFALCFLPFHVTRTLYYS   270
P2Y2-Takifugu         208   EFSWRS---------EGRAEGKSVKMIIIVLATFMLCFLPFHLTRSLYYS   248
P2Y2-Tetraodon        216   EVSWRS-GAKRGNLSARQSRQKSVKMIIIVLATFMLCFLPFHLTRSLYYS   264
P2Y2-Medaka           242   EPGWGP-GGE--VLTAQRTKQKSVKMIIIVLAAFMLCFLPFHLTRSLYYS   288
P2Y2-1-Stickleback    226   EPGWASEGGERGGLSARRLKQKSVKMIIVVLATFMICFLPFHLTRSLYYS   275
P2Y2-2-Stickleback    218   EPGWASEGGTRGGLSARRLKQKSVEMIIVVLAMFILCFLPFH--------   259
P2Y2-1-Danio          218   EPGVGD------GPMSQKSKQKSVKMIIIVLVAFMLCFLPFHVNRSIYYG   261
P2Y2-2-Danio          219   EPRVGG------GSTSQRSKQKSVKMIILVLLTFILCFLPFHVNRIVHYT   262
P2Y2-3-Danio          235   EPGVGD------APTSQRSKQKSVKMIIIVLVAFMLCFLPFHVNRSVYYT   278
P2Y2-Callorhinchus    205   RPNTAH-------PENSKSRKKSIKMIVIILSVFVLCFLPFHITRSIYYS   247

                                      267a
                                       |                    TM7
MC5R-Human            264   CPQ--------NLYCSRFMSHFNMYLI---LIMCNSVMDPLIYAFRSQEM   302
P2Y2-Human            271   FRSL-------DLSCHTLNAINMAYKVTRPLASANSCLDPVLYFLAGQRL   313
P2Y2-Takifugu         249   FRYLRQISP-QQITCNTLEAINVAYKVTRPLASANSCLDPILYFLAGQDI   297
P2Y2-Tetraodon        265   FRYIR------QITCGMLEAINVAYKVTRPLASANSCLDPILYFLAGQDI   308
P2Y2-Medaka           289   LRYLR------KVSCSLLESSSVAYKVTRPLASANSCVDPILYFLAGQGT   332
P2Y2-1-Stickleback    276   FRYLRQVDPSQA-------SSVAYKVTRPLASTNSCVDPILYFLAGQNVR   318
P2Y2-2-Stickleback      -   --------------------------------------------------     -
P2Y2-1-Danio          262   FRYLDK----QVSCSMLEIASMAYKITRPLASANSCIDPILYFMAGQGFK   307
P2Y2-2-Danio          263   LRYM------DRSCDQLRLSNIYYQATRPLLSINSCIDPILYFMAGQSFR   306
P2Y2-3-Danio          279   LRYLD-----QANCTVIRLSNNVYKATRPLVSFNSCIDPILYFMAGQSFR   323
P2Y2-Callorhinchus    248   SRSL------HLSCSTLNAVNLAYKLTRPLASANSCLDPILYVLAGQ---   288

MC5R-Human            304   RKTFKEIICCRGFRIACSFPRRD---------------------------   325
P2Y2-Human            315   VRFARDAKPPTGPSPATPARRRLGLRRSDRTDMQRIEDVLGSSEDSRRTE   363
P2Y2-Takifugu         299   RSNLTKKSRTTTHKAGMKMCERKHRQITAMLHTAVTSPFMTS--------   339
P2Y2-Tetraodon        310   RSNLTKKCRTANQKLGT---------------------------------   325
P2Y2-Medaka           334   RSSFIR--------------------------------------------   338
P2Y2-1-Stickleback    319   SSLTKKIR------------------------------------------   326
P2Y2-2-Stickleback      -   --------------------------------------------------     -
P2Y2-1-Danio          308   SSIKRNKSPVARSGKKPPSTSL----------------------------   329
P2Y2-2-Danio          307   NNSTRSNPSSERSISTV---------------------------------   323
P2Y2-3-Danio          324   KSIKKTSSQRYDKSMSTY--------------------------------   341
P2Y2-Callorhinchus      -   --------------------------------------------------     -
```

**MC5R-Human**	**-**	**--------------**	**-**
**P2Y2-Human**	**364**	**STPAGSENTKDIRL**	**377**
**P2Y2-Takifugu**	**-**	**--------------**	**-**
**P2Y2-Tetraodon**	**-**	**--------------**	**-**
**P2Y2-Medaka**	**-**	**--------------**	**-**
**P2Y2-1-Stickleback**	**-**	**--------------**	**-**
**P2Y2-2-Stickleback**	**-**	**--------------**	**-**
**P2Y2-1-Danio**	**-**	**--------------**	**-**
**P2Y2-2-Danio**	**-**	**--------------**	**-**
**P2Y2-3-Danio**	**-**	**--------------**	**-**
**P2Y2-Callorhinchus**	**-**	**--------------**	**-**
