# Supplementary material for: Spliceosomal Intron Insertions in Genome Compacted Ray-Finned Fishes as Evident from Phylogeny of MC Receptors, Also Supported by a Few Other GPCRs
Source: PLoS One. 2011 Aug 5;6(8):e22046. doi: 10.1371/journal.pone.0022046 (PMC3151243; doi:10.1371/journal.pone.0022046)
Supplement: Figure S8 — Intron insertions in P2Y3-like (P2Y3L) receptor during diversification of ray-finned fishes. One novel intron is inserted at position 187c (human MC5R amino acid numbering with suffix a–c for intron phasing) in the P2Y3L receptor of four fishes - Takifugu, Tetraodon, stickleback and medaka (blue background), but not in P2Y3L genes from zebrafish, elephant shark and tetrapods. There is also species-specific introns in the P2Y3L gene such as S212a for stickleback, M234a in medaka and T252a for Takifugu (numbering is species specific numbering, due to gaps in the region and red background), localized in the loop between TM5 and TM6. Residues conserved above 70% are marked by white on black background. ### indicates location of highly conserved DRY motif. Gallus P2Y3 receptor was used as it a typical representative of tetrapod P2Y3. Transmembrane regions are marked as TM1–TM7 (yellow bars) as predicted by TMHMM2.0 [106]. (PDF) [file pone.0022046.s008.pdf]

Figure S8.

```
                                                                      TM1
MC5R-Human           1 MNSSFHLHFLDLNLNATEGNLSGPNVKNKSSP-CEDMGIAVEVFLTLG--   47
P2Y3-Gallus          1 --------------------MSMANFTGGRNS-CTFHEEFKQVLLPLVYS   29
P2Y3L-Takifugu       1 -----PPHVGVTILQEFAGTFSPPPSGTHQPPSCSIDESYKYIFLPICYS   45
P2Y3L-Medaka         1 -----STNISAAVIQDVLSSPYSTYSSTHSTSSCSIDESYKYIFLPICYS   45
P2Y3L-Stickleback    1 --------------------SSTPSTTTPSPS-CSIDESYKYIFLPICYS   29
P2Y3L-Danio          1 RSLRTMTTSWIETVTGVDARLSSSANSSGNGT-CNIDESYKYIFLPICYS   49
P2Y3-Callorhinchus   1 ---------------------------------CPLYEDYKHTLLPVTYS   17

                                                                 TM2
MC5R-Human          48 ---VISLLENILVIGAIVKNKNLHSPMYFFVCSLAVADMLVSMSSAWETI   94
P2Y3-Gallus         30 VVFLLGLPLNAVVIGQIWLARKALTRTTIYMLNLAMADLLYVCS--LPLL   77
P2Y3L-Takifugu      46 FTFIFSISLNSVILYRSVCRTKRWNASLIYMVNLATTDFMYGLS--LPFL   93
P2Y3L-Medaka        46 FTFIFSITLNSVVLYRSFR-TKRWNTSLIYMVNLASTDFMYGLS--LPFL   92
P2Y3L-Stickleback   30 FTFLFSISLNSVILFRSFRQTKRWNPSLIYMVNLASTDFMYGLS--LPFL   77
P2Y3L-Danio         50 FTFVFSITLNFVVLYRSFRRTRRWNASLIYMVNLATTDFMYGLS--LPFL   97
P2Y3-Callorhinchus  18 LVFLLGLTLNGAVLCLSWRLTRKWSCSTIYLVNLAVADLLYVCS--LPLL   65

                                                    TM3         ###
MC5R-Human          95 TIYLLNNKHLVIADAFVRHIDNVFDSMICISVVASMCSLLAIAVDRYVTI  144
P2Y3-Gallus         78 IYNYTQKDYWPFGDFTCKFVRFQF----YTNLHGSILFLTCISVQRYMGI  123
P2Y3L-Takifugu      94 VASYIMRDCWVFGDFMCRLVRFLF----YFNLYCSIFFLTCISVHRYLGI  139
P2Y3L-Medaka        93 IASYIMQDRWIFGDFMCRLVRFLF----YFNLYCSIFFLTCISVHRYLGI  138
P2Y3L-Stickleback   78 VASYVMRDRWVFGDFMCRLVRFLF----YFNLYCSIFFLTCISVHRYLGI  123
P2Y3L-Danio         98 VASYIMQDDWVFGDFMCRLVRFLF----YFNLYCSIFFLTCISVHRYLGI  143
P2Y3-Callorhinchus  66 ILNYIWRDRWPFGDFLCRLVRFLF----YANLYGSILFLTCISVHRYLGL  111

                                                                 187c
                                          TM4
MC5R-Human         145 FYAL-RYHHIMTARRSGAIIAGIWAFCTGCGIVFILYSESTYV--I----  187
P2Y3-Gallus        124 CHPLASWHKKKGKKLTWLVCAAVWFIVIAQCLPTFVFASTGTQRNR---T  170
P2Y3L-Takifugu     140 CHPM-RVITLETKKAVKCTCVVVWIVVFALTCPIFRFAQTGYVSRR---R  185
P2Y3L-Medaka       139 CHPM-KVISLETKKTVKYTCVLVWIVVFALTCPIFRFAQTGCVT-S---V  183
P2Y3L-Stickleback  124 CHPM-KVITLETKKAVKSTCVLVWIVVFALTCPIFRFAQTG----E----  164
P2Y3L-Danio        144 CHPM-KTITLESKRAVKGTCVLVWIAVFALTCPIFRFAQTQYLPREGKGT  192
P2Y3-Callorhinchus 112 CFPM-KSIEWRTRRLAVETCVVAWIMVGLQVVPTYLYAHTCSLANK---T  157

                                      TM5
MC5R-Human         188 L------CL---------ISMFFAMLFLLVSLYIHMFLLARTHVKRIAAL  222
P2Y3-Gallus        171 V------CYDLSPPDRSTSYFPYGITLTITGFLLPFAAILACYCSMARIL  214
P2Y3L-Takifugu     186 VVEEYQNCWDDAIDKEFPDYIPYGITLHLLGFFVPFSIIAWCYSHVVLTI  235
P2Y3L-Medaka       184 VIEQYQNCWDDAIDKEFPVYVPYGIILHLLGFFVPFSIIAWCYSHVVLTI  233
P2Y3L-Stickleback  169 ----YRNCWDDAIDKEFPDYVPYGIVLHLLGFFVPFSIIAWCYSHVVLTI  214
P2Y3L-Danio        193 REEVFNNCWDDAIDREFHDYIPYGVTLHFLGFFMPFSIIAWCYSRVVLTI  242
P2Y3-Callorhinchus 158 V------CYDLTSPDNFHNYLPYGTVLTVTGFVIPFVIIILSYCCIIKTL  201

                       S212a                              M234a     T252a
                       |                                  |         |
MC5R-Human         223 ---------------------------------PGASSAR-----QRTSM  234
P2Y3-Gallus        215 ----------------------------------CQKDELIGLAVHKKKD  230
P2Y3L-Takifugu     236 FR-----------------------------TLHSQPSSYRIPRGKSRRR  256
P2Y3L-Medaka       234 ----------------------------------CAHSPY-----ANRRR  244
P2Y3L-Stickleback  212 LRR-----------------------DEGTSIFLGAHSPY-----ANRRR  233
P2Y3L-Danio        243 FRTLNSEPPSQRARSGTGRESVAGIGGEGMSIILGARSPY-----VNRRR  287
P2Y3-Callorhinchus 202 -------------------------------IVSGDSTQLQ----KPVRT  216
```

```
                                      TM6
MC5R-Human           235 QGAVTVTMLLGVFTVCWAPFFLHLT--LMLSCPQNLYCSRFMSHFNMYLI 282
P2Y3-Gallus          231 KAVRMIIIVVIVFSISFFPFHLTKTIYLIVRSSASLPCPTLQAFAIAYKC 280
P2Y3L-Takifugu       257 KSIKTIITITLLFALCFFPFHVTRTIFLLLKVAKGVPCHTMTTVSMCYKI 306
P2Y3L-Medaka         245 KSIRTIIAITLLFALCFFPFHVTRTIVLLLKVSRRVPCHTMTTVSICYKI 294
P2Y3L-Stickleback    234 KSIKTIITITLLFALCFFPFHVTRTIFLVLKVTKGVPCHTMTMVSMCYKI 283
P2Y3L-Danio          288 KSIKTIITITLLFALCFFPFHVTRTIFLLLKLTSRVQCHTMRMVSICYKI 337
P2Y3-Callorhinchus   217 KSIRTIVLVCGLLAVCFVPFHITRTIYLFVRAYLAQDCNILQTVSLAYKI 266

                                 TM7
MC5R-Human           283 ---LIMCNSVMDPLIYAFRSQEMRKTFKEIICCRGFR-----------IA 318
P2Y3-Gallus          281 TRPFASMNSVLDPILFYFTQRKFRESTRYLLDKMSSK-------WRQDHC 323
P2Y3L-Takifugu       307 TRPLASFNAWLNALLYFLTKD----------------------------- 327
P2Y3L-Medaka         295 TRPLASFNAWLNALLYFLTKDKMG-----NHCCQAANTTSQ---QREGLL 336
P2Y3L-Stickleback    284 TRPLASFNAWLNALLYFLTKDK---------------------------- 305
P2Y3L-Danio          338 TRPLASFNAWLNALLYFLTKEKNG------PCCQKPEHAQHGLLWPLRML 381
P2Y3-Callorhinchus   267 WRPVISFNSCINPLLYFLSGD----------------------------- 287

MC5R-Human           319 CSFPRRD 325
P2Y3-Gallus          324 ISYGS-- 328
P2Y3L-Takifugu         - -------   -
P2Y3L-Medaka         337 LPLRIMD 343
P2Y3L-Stickleback      - -------   -
P2Y3L-Danio          382 GKGEEEE 388
P2Y3-Callorhinchus     - -------   -
```
