# Supplementary material for: Spliceosomal Intron Insertions in Genome Compacted Ray-Finned Fishes as Evident from Phylogeny of MC Receptors, Also Supported by a Few Other GPCRs
Source: PLoS One. 2011 Aug 5;6(8):e22046. doi: 10.1371/journal.pone.0022046 (PMC3151243; doi:10.1371/journal.pone.0022046)
Supplement: Figure S9 — Intron insertions in P2Y6 receptor during diversification of ray-finned fishes. One novel intron is inserted at positions 120c (human MC5R amino acid numbering with suffix a–c for intron phasing) in the P2Y6 receptor of four fishes - Takifugu, Tetraodon, stickleback and medaka (blue background), but not in P2Y2 genes from zebrafish, and tetrapods. Residues conserved above 70% are marked by white on black background. ### indicates location of highly conserved DRY motif. Transmembrane regions are marked as TM1–TM7 (yellow bars) as predicted by TMHMM2.0 [106]. (PDF) [file pone.0022046.s009.pdf]

Figure S9.

```
MC5R-Human          1   -------------------MNSS------------FHLHFLDLNLNATEGNL    21
P2Y6-Human          1   -------------------MAAD-------------LGPWNDTINGTWDGDE    20
P2Y6-Takifugu       1   -------------------FFTEGVISLTSLDFASSEPFTGPNTHNIIKGI    32
P2Y6-Tetraodon      1   ---------MPGSAHLGPFLTESVISRTSLAFPSSEPFADLNNTNNNTN-    40
P2Y6-Medaka         1   -------------------TEPY-------------GADIFSVGNSSSVAET    20
P2Y6-Sickleback     1   LGNPLLFSRRLLAGSVHLFQTASCEWETETLISDSFPSDSFSTSAWPPET    50
P2Y6-Danio          1   -------------------LKMD----------------------SLNVSTM    11

                                                             |       TM1
MC5R-Human         22   SGPN-------------VKNKSSPCEDMG--IAVEVFLTLGVISLLENIL    56
P2Y6-Human         21   LGYR--------------CRFN----EDFKYVLLPVSYGVVCVPGLCLNAV    53
P2Y6-Takifugu      33   SNSN--------------CTYK----EDFKRILLPTVYTMVCLLGLPLNTV    65
P2Y6-Tetraodon     41   ------------------CTYK----EDFKRILLPAVYTLVLLLGLPLNAV    69
P2Y6-Medaka        21   ASPR--------------CTYK----EDFKQILLPAVYSIVFLLGFPLNAA    53
P2Y6-Sickleback    51   YAADGFVANVSVSGALRCTYK----EDFKRILLPAVYTLVFLLGLPLNVA    96
P2Y6-Danio         12   ALPS--------------CTYK----EDFKRFLLPSVYSLVFIFGLPLNFI    44

                     |                |         TM2            |
MC5R-Human         57   VIGAIVKNKNLHSPMYFFVCSLAVADMLVSMSSAWETITIYLLNNKHLVI   106
P2Y6-Human         54   ALYIFLCRLKTWNASTTYMFHLAVSDALYAAS--LPLLVYYYARGDHWPF   101
P2Y6-Takifugu      66   VILKIWRARPNLSRSNIYMLNLAIADFLYVMS--LPLLVYNYGSRDYWPF   113
P2Y6-Tetraodon     70   VILKIWRTRPNLSRNNIYMLNLAIADFLYVTS--LPLLIYNYGSRDYWPF   117
P2Y6-Medaka        54   VILKIWRTRPNLSRSNIYMLNLALADFLYVMS--LPLLIYNYASHDYWPF   101
P2Y6-Sickleback    97   VILKIWRTRPKLSKSNVYMLNLAIADFLYVMS--LPLLIYNYGSHDHWPF   144
P2Y6-Danio         45   IILRVWGSRRALTRTKIYMLNLAVADFLYVCS--LPLLIYNYASKDYWPF    92

                                    120c
                                  |  |       TM3         |###
MC5R-Human        107   ADAFVRHIDNVFDSMICISVVASMCSLLAIAVDRYVTIFYALRYHHIMTA   156
P2Y6-Human        102   STVLCKLVRFLFYTNLYCSILFLTC----ISVHRCLGVLRPLRSL-RWGR   146
P2Y6-Takifugu     114   GEFTCKLVRFQFYSNLHGSILFLTC----ISVQRYVGVCHPLAMWHKKGG   159
P2Y6-Tetraodon    118   GELTCKLVRFQFYSNLHGSILFLTC----ISVQRYVGICHPLAMWHKRGG   163
P2Y6-Medaka       102   GEFTCKLVRFQFYSNLHGSILFLTC----ISMQRYMGICHPLAVWHKQGG   147
P2Y6-Sickleback   145   GEFTCKLVRFQFYSNLHGSILFLTC----ISVQRYVGICHPMAMWPRQGG   190
P2Y6-Danio         93   GDFTCRLVRFQFYSNLHGSIFFLTC----ISFQRYMGICHPLSLWPKQGG   138

                          |         TM4          |                       |
MC5R-Human        157   RRSG-AIIAGIWAFCTGCGIVFILYSES-------------------TYV   186
P2Y6-Human        147   ARYARRVAGAVWVLVLACQAPVLYFVTTSARGGRVTCHDTSAPELFSRFV   196
P2Y6-Takifugu     160   RKMAWRVCGGVWLLVIALCAPTFHFAATGVQRNRTVCYDLSTPAHSVDYY   209
P2Y6-Tetraodon    164   RKMAWRVCGAVWLLVIALCAPTFHFAETGIQRNRTVCYDLSTPARSVDYY   213
P2Y6-Medaka       148   RKKVWLICGGVWLLVITLCAPTFYFASTGTQRNRTVCYDLSTPQRSGDYY   197
P2Y6-Sickleback   191   IRLAWCICGGVWLVVVVLCAPTFHFAATGTQRNRTVCYDLSTPKSSLDYY   240
P2Y6-Danio        139   RRLAWWVCAGVWVAVAILCAPTFEFASTGIQRNRTVCYDLSEPSRSTSYF   188

                                  TM5          |
MC5R-Human        187   ILCLISMFFAMLFLLVSLYIHMFLLARTHVKRIAALPGASSARQRTSMQG   236
P2Y6-Human        197   AYSSVMLGLLFAVPFAVILVCYVLMARR-LLKPAY--GTSGGLPRAKRKS   243
P2Y6-Takifugu     210   PYGMALTCLGFLLPFLGIMVCYCRMAQI-LCRPVSYQGVSMATKEKRNKA   258
P2Y6-Tetraodon    214   PYGMALTCLGFVLPFLGVMVCYCRMAQI-LCRPVSYRGVSMATGEKRDKA   262
P2Y6-Medaka       198   PYGLALTLLGFLLPLIGVMVCYCQMARI-LCRPVSHQGVPMATGEKRDRA   246
P2Y6-Sickleback   241   PYGMALTCLGFLLPFIGVMVCYCRMACI-LCRPVTYRGVSMATGEKRDQA   289
P2Y6-Danio        189   PYGIALTFLGFVVPFLVIIVLYCKMARV-LWQVGEAREVSAA--EKKNKA   235
```

```
                                       TM6
MC5R-Human       237  AVTVTMLLGVFTVCWAPFFLHLTLMLSCPQ--NLYCSR---FMSHFNMYL  281
P2Y6-Human       244  VRTIAVVLAVFALCFLPFHVTRTLYYSFRSL-DLSCHTLNAINMAYKVTR  292
P2Y6-Takifugu    259  VRMIIVVATVFCISFLPFHLTKTVYLVVRSFPGTPCETKNLFSVIYKSTR  308
P2Y6-Tetraodon   263  VKMIIVVAAVFCISFLPFHFTKTVYMMVRSIQGMPCTTRNLFSVIYKSTR  312
P2Y6-Medaka      247  VRMIIVVASVFCVSFLPFHLTKTVYQVVRTLPHASCETRNLFSIIYKSTR  296
P2Y6-Sickleback  290  VRMIIVVATVFCISFLPFHFTKTVYLVVRTLPGAPCETRNLFSIIYKSTR  339
P2Y6-Danio       236  VRMIIIVMLVFAISFLPFHVTKTLYLLVRSLPTAPCELRNLLSVVYKSTR  285

                            TM7
MC5R-Human       282  ILIMCNSVMDPLIYAFRSQE-------------------------MRKTF  306
P2Y6-Human       293  PLASANSCLDPVLYFLAGQRLVRFARDAKPPTGPSPATPARRRLGLRRSD  342
P2Y6-Takifugu    309  PFASMNSFLDPILFYFTQPR-------------------------YRQST  333
P2Y6-Tetraodon   313  PFASMNSFLDPILFYFTQPR-------------------------YRQST  337
P2Y6-Medaka      297  PFASMNSFLDPILFYFTQPR-------------------------YRRST  321
P2Y6-Sickleback  340  PFASMNSFLDPILFYFTQPR-------------------------YR---  361
P2Y6-Danio       286  PFASMNSVLDPILFYFTQPQ-------------------------YRRST  310

MC5R-Human       307  KEIICCRGFRIACSFPRRD----------------  325
P2Y6-Human       343  RTDMQRIEDVLGSSEDSRRTESTPAGSENTKDIRL  377
P2Y6-Takifugu    334  KRFVLRVTTLKG-----------------------  345
P2Y6-Tetraodon   338  RRFVLRV----------------------------  344
P2Y6-Medaka      322  RRLLLRVTARGDKAPPTLKKHSTSASLYCRYEL--  354
P2Y6-Sickleback    -  -----------------------------------    -
P2Y6-Danio       311  KSLLLKITSLKDKTVKV------------------  327
```
