# Supplementary material for: Spliceosomal Intron Insertions in Genome Compacted Ray-Finned Fishes as Evident from Phylogeny of MC Receptors, Also Supported by a Few Other GPCRs
Source: PLoS One. 2011 Aug 5;6(8):e22046. doi: 10.1371/journal.pone.0022046 (PMC3151243; doi:10.1371/journal.pone.0022046)
Supplement: Figure S10 — Intron insertions in CHRM3 receptor during diversification of ray-finned fishes. There are two introns inserted at positions 67a, 166c (DRY intron or 140c by human MC5R numbering) in CHRM3 receptor of four fishes - Takifugu, Tetraodon, stickleback and medaka (blue background), but not in P2Y2 genes from zebrafish, elephant shark and tetrapods. Various species-specific introns in the large loop between TM5 and TM6 are marked by red background. Residues conserved above 70% are marked by white on black background. ### indicates location of highly conserved DRY motif. In this case, human CHRM3 amino acid numbering was followed as human MC5R was not suitable due to variable size of these receptor proteins with suffix a–c for intron phasing. Transmembrane regions are marked as TM1–TM7 (yellow bars) as predicted by TMHMM2.0 [106]. (PDF) [file pone.0022046.s010.pdf]

Figure S10.

```
CHRM3-Human         1   MTLHNNSTTSPLFPNISSSWIHSP--SDAGLPPGTVTHFGSYNVSRA---    45
CHRM3-Takifugu      1   ----------------------------------------MQEANLSYSS    10
CHRM3-Tetraodon     1   -----------------------------GDWTHHNVSHVLRSEGNMT-QS    21
CHRM3-Medaka        1   ---------------------------------------------FTDFQ     5
CHRM3-Stickleback   1   MNLTSSSDSIHFLTNRSSVFMDSPDLSVGGWKQHNVTHISRQGGNFTDSP    50
CHRM3-Danio         1   SALCTEYATMNLTLSAEPGLFLTN--SSPGMRANPVTAFMSHDLHPANDS    48
CHRM3-Eshark        -   --------------------------------------------------     -

                                              67a
                                              |   |        TM1        |
CHRM3-Human        46   AGNFSSPDGTTDDPLGGHTVWQVVFIAFLTGILALVTIIGNILVIVSFKV    95
CHRM3-Takifugu     11   ANGTWLPPSDSFDPLGGHTVWQVIIIVFLTGSLSLITVVGNILVLLSFKV    60
CHRM3-Tetraodon    22   LNGTWLPPADSFDPLGGHTVLQVIIIIFLTGSLSLVTVVGNILVLLSFKV    71
CHRM3-Medaka        6   AGRMSVLPTEKTNGSGGDPLWQVIIIVFLTGSLSVITVVGNILVLISFKI    55
CHRM3-Stickleback  51   VNGTLILPAEDFDPLGGHTVLQVIIIVFLTGSLSLVTVVGNILVLVSFKI   100
CHRM3-Danio        49   LHNITQIPSVLSDPLGGHSVLQVVMIALLCGSISLLTIIGNTLVLLSFKL    98
CHRM3-Eshark        1   ----------------GPSLLKVILIATITAIVSLVTIIGNILVIVSFKV    34

                                   |        TM2         |                 |
CHRM3-Human        96   NKQLKTVNNYFLLSLACADLIIGVISMNLFTTYIIMNRWALGNLACDLWL   145
CHRM3-Takifugu     61   NKALKTVNNYYLLSLAFADLTIGTLSMNLYTTYIIMDQWALGPVVCDLWL   110
CHRM3-Tetraodon    72   NKALKTVNNYYLLSLAFADLTIGTLSMNLYTTYIIMDQWALGPVVCDLWL   121
CHRM3-Medaka       56   NKTLKTVNNYYLLSLAFADLIIGTLSMNLYTTYIIMGHWALGPVLCDLWL   105
CHRM3-Stickleback 101   NKALKTVNNYYLLSLAFADLTIGTLSMNLYTTYIIMDQWALGPVVCDLWL   150
CHRM3-Danio        99   NKQLKTVNNYYLLSLAFADLIIGVLSMNLYTTYIIMDQWALGNFACDLWL   148
CHRM3-Eshark       35   NSQLKTVNNYYLLSLACADLIIGVFSMNLYTSYILVGHWSLGTLACDLWL    84

                                              166c
                           TM3              | |                   |
CHRM3-Human       146   AIDYVASNASVMNLLVIS-FDRYFSITRPLTYRAKRTTKRAGVMIGLAWV   194
CHRM3-Takifugu    111   AIDYVASNASVMNLLVIS-FDRYFSVTRPLTYRAKRTTKRAMTMICLAWS   159
CHRM3-Tetraodon   122   AIDYVASNTSVMNLLVISVLFRYFSVTRPLTYRAKRTTKRAMTMICLAWS   171
CHRM3-Medaka      106   AIDYVASNASVMNLLIIS-FDRYFSVTRPLTYRAKRTTKRTMAMIGLAWS   154
CHRM3-Stickleback 151   AIDYVASNASVMNLLVIS-FDRYFSVTRPLTYRAKRTTKRAMTMIGLAWS   199
CHRM3-Danio       149   AIDYVASNASVMNLLVIS-FDRYFSVTRPLTYRAKRTTKRAMTMIGLAWS   197
CHRM3-Eshark       85   ALDYVASNASVMNLLAIS-FDRYFSITRPLTYRAKRTPKRAAIMIGLAWM   133
                                           ###

                            TM4      |                      |        TM5
CHRM3-Human       195   ISFVLWAPAILFWQYFVGKRTVPPGECFIQFLSEPTITFGTAIAAFYMPV   244
CHRM3-Takifugu    160   ISFILWAPAILFWQYIVGERTVQPNECYIQFLSEPIITFCTAIAAFYLPV   209
CHRM3-Tetraodon   172   ISIILWAPAILFWQYIVGERTVQPNECYIQFLSEPIITFCTAIAAFYLPV   221
CHRM3-Medaka      155   ISFVLWAPAILFWQYVVGERTVPPEECYIQFLTEPVTTFCTAIAAFYLPV   204
CHRM3-Stickleback 200   ISFILWAPAILFWQYIVGERTVQPNECYIQFLSEPIITFSTAVAAFYLPV   249
CHRM3-Danio       198   ISFILWAPAILFWQYFVGERTVEPDKCYIQFISEPITTFCTAIAAFYLPV   247
CHRM3-Eshark      134   ISFILWAPAILCWQYLVGERTVPSSDCQIQFLSEPIITFGTAIAAFYIPV   183

                                                           282c
                              |                             |
CHRM3-Human       245   TIMTILYWRIYKETEKRTKELAGLQASGTEAETENFVHP-TGS-------   286
CHRM3-Takifugu    210   TIMAILFWKIYQETEKRAKEVQGLKGSGAGHGPTQPKNQGGAASGREG--   257
CHRM3-Tetraodon   222   TIMAILFWKIYQETEKRAKEVQGLKGSGTGNGPTQPKKQRQSS-------   264
CHRM3-Medaka      205   TVMAFLFWKIYQETEKRAKDIQSLKGS-----------------------   231
CHRM3-Stickleback 250   TIMSFLFWKIYQETEKRVKDMQGLKASGSGDSQSQSQKQGSGKGRASGEI   299
CHRM3-Danio       248   TIMTVLYWRIYKETENRSRELAGLKASGNVEEESVVVVPQTGS-------   290
CHRM3-Eshark      184   SVMTILYCRIYKETERRTKDLAELQGSNLGSENEIASASKKTL-------   226
```

```
CHRM3-Human        287   ---------------SRSCSSYELQQQSMKRSNRRKY----G-----RCH   312
CHRM3-Takifugu     258   ANNSQKDTSSVQCQGSQTCSSHDINQPAAEKSNKSNEANAER-----KHG   302
CHRM3-Tetraodon    265   ---------------SQTCSSYDLDQPASEKKKSNEA-NAEG-----KHG   293
CHRM3-Medaka       232   ------------------CASTNLNQTVSEMSE--------------GVG   249
CHRM3-Stickleback  300   ATNSQKDSSAIS---SQSCSSCELNQLASDKNSKKNAGIPGGTGSKARCG   346
CHRM3-Danio        291   ---------------SRSHSSYELQPAAPMRKTKT------------RRL   313
CHRM3-Eshark       227   ---------------FRSCFGCE----GHKRSNRE------------RCQ   245


CHRM3-Human        313   -FWFTTKSWKPSSEQMDQDHSSSDSWNNNDAAASLENSASS------DEE   355
CHRM3-Takifugu     303   TFWQRISF-------MFLSHRSAKRSANNVTSGCEAEQSSYD-----SSN   340
CHRM3-Tetraodon    294   AFWLRISL-------LCFSHRSGKRPGNNVAAACDRRKVAVTILLKSALQ   336
CHRM3-Medaka       250   EGSIKSNQRNTSVKLHLVRTSSSRKLCHQDGPARDPRGSDF------DSA   293
CHRM3-Stickleback  347   ASCFQFSL-------LLSDRNASKRSVNSTTTIVGETEQS-------SCD   382
CHRM3-Danio        314   WFWSRGDV----------ERSSSDSWNNNETAISIDQ----------SDD   343
CHRM3-Eshark       246   VSWSSSSRSTTVTLRTTHAQSVVDEWSKSDKAGSFASYPS-------SDD   288

                             361b                        383b
                               |                           |
CHRM3-Human        356   DIGSETRAIYSIVLKLP-----GHSTILNSTKLPSSDNLQVPEEELGMVD   400
CHRM3-Takifugu     341   NVEVCATGDHS---GSE-----EELNGESPSTEYIHMNAAV-------FY   375
CHRM3-Tetraodon    337   EISQVQRRNWLV--RVH-----QQVRHYHLPTLFVCIIYKNNTCLLSALY   379
CHRM3-Medaka       294   SQSPTVCLEFE---------------------------------------   304
CHRM3-Stickleback  383   SLNNNQAGEQS---GSE-----DESDGADPTKAPT---------------   409
CHRM3-Danio        344   DEEEDAEHIYTI--------------------------------------   355
CHRM3-Eshark       289   EEKPTTKSVFSVSFKTQANNQKEEFNSEEDKKISLEDHPMINDYENSNCL   338

                         402b
                           |
CHRM3-Human        401   LE-RKADKLQAQKSVDDGGSFPKSFSKLPIQLESAVDTAKTSDVNSSVGK   449
CHRM3-Takifugu     376   LDDKKCKRMKKNKDRKRSSSRSQQ---------DSQTNHQGSPESTVSTA   416
CHRM3-Tetraodon    380   LDDKKYRKMKKNKDRQSSSSRSQQ---------GLQSNHQGSAENAASAA   420
CHRM3-Medaka       305   ----KSVEEEENKDLEQTSTIKRQ---------------HGSPLFKASSS   335
CHRM3-Stickleback  410   -DAKKSGRVKNNKDKHSSNKSGKG---------------SQSNPVNSSTA   443
CHRM3-Danio        356   ----------EKQDLEAKPSKFGV-------------------SKSSTL   375
CHRM3-Eshark       339   VSPAKCQKQKDKKCVSYKFNKLTI----------TNSSFQPTPDSSNGCT   378

                                           470c
                                             |
CHRM3-Human        450   STATLPLSFKE--ATLAKRFALKTRSQITKRKRMSLVKEKKAAQTLSAIL   497
CHRM3-Takifugu     417   TDRSGGITL-------------KAKVEINKRKNEKKANDKKAARTLSAIL   453
CHRM3-Tetraodon    421   ADRSGGITMKD--AAMAKRFASKAKVEINKRKNEKKANDKKAARTLSAIL   468
CHRM3-Medaka       336   NLPLGTMTIKD--AALAKRFALRAKPEINKHKNEKKANEKKAARTLSAIL   383
CHRM3-Stickleback  444   DQSTAAITIKD--ATMAKRFASKAKTEVNKRKNEKKANEKKAARTLSAIL   491
CHRM3-Danio        376   DSKDQPV---------------KSARKTNKRKKLSLVKEKKAAQTLSAIL   410
CHRM3-Eshark       379   QIKITPCSSTDSEAPFIKNLDQNLKNQITKRKRMVLVKERKAAQTLSAIL   428

                                 TM6                          TM7
CHRM3-Human        498   LAFIITWTPYNIMVLVNTFCDSCIPKTFWNLGYWLCYINSTVNPVCYALC   547
CHRM3-Takifugu     454   FAFITTWLPYNIMVLVNTFCQDCIPQTLWALGYWLCYVNSTVNPMCYALC   503
CHRM3-Tetraodon    469   FAFITTWLPYNIMVLVNTFCQDCIPETLWALGYWLCYVNSTVNPMCYALC   518
CHRM3-Medaka       384   LAFMMTWLPYNIMVLINTFCQNCIPEALWALGYWLCYVNSTVNPMCYALC   433
CHRM3-Stickleback  492   FVFITTWLPYNIMVLVNTFCQDCIPETLWALGYWLCYVNSTINPICYALC   541
CHRM3-Danio        411   LAFIITWTPYNIMVLLNAFCDGCIPETLWALGYWLCYVNSTVNPMCYALC   460
CHRM3-Eshark       429   LAFIITWTPYNIMVLVSTFCSNCIPVSLWHLGYWLCYVNSTVNPMCYALC   478
```

```
CHRM3-Human        548    NKTFRTTFKMLLLCQCDKKKRRKQQYQQRQSVIFHKRAPEQAL    590
CHRM3-Takifugu     504    NKTFRTTFRDILMCQWNQKK-NKPKFQERKN--FPKP------    537
CHRM3-Tetraodon    519    NKTFRTTFRDILMCQWNQKK-NKPKFQERLTVSFHKRAGEIPK    560
CHRM3-Medaka       434    NKTFRTTFRAILMCQWNGRK--KPHPHQRQA--FQTKNVRKAR    472
CHRM3-Stickleback  542    NKTFRTTFRDILMCQWNQKK-NQPNFPQRKAVAFKKKDPI---    580
CHRM3-Danio        461    NKAFRTTFRSILLCQWRKKKLNKAQIQKRAPAATYRKHTQSL-    502
CHRM3-Eshark       479    NKTFRKTFKTLLLCQWKNKR-NEEKLN------WYSQHP----    510
```
