# Supplementary material for: Spliceosomal Intron Insertions in Genome Compacted Ray-Finned Fishes as Evident from Phylogeny of MC Receptors, Also Supported by a Few Other GPCRs
Source: PLoS One. 2011 Aug 5;6(8):e22046. doi: 10.1371/journal.pone.0022046 (PMC3151243; doi:10.1371/journal.pone.0022046)
Supplement: Table S2 — List of CHRM3 genes from fishes. Codon usage in DRY motif is shown and codons of the R residue is marked by red color. The absence of intron is marked by grey background in R residue. (DOC) [file pone.0022046.s012.doc]

**Table S2.**

| **Gene** | **Ensembl Accession id** | **Genomic location** | **DRY intron** |
| --- | --- | --- | --- |
| TRHR3a-Takifugu | ENSTRUG00000007726 | scaffold_226: 428,680-431,390 | D--R— Y  GAACGCTAC |
| TRHR3b-Takifugu | ENSTRUG00000008184 | scaffold_226: 455,040-456,647 | D--R— Y  GAACGCTAC |
| TRHR3-Tetraodon | ENSTNIG00000000179 | Chr9: 9,343,075-9,344,477 | D--R— Y  GAACGCTAC |
| TRHR3-Medaka | ENSORLG00000015111 | Chr7: 22,477,503-22,480,808 | E R Y  GAGCGCTAC |
| TRHR3-Stickleback | ENSGACG00000011777 | groupXII: 15,499,788-15,501,673 | E R Y  GAGCGATAC |
