# Supplementary material for: Spliceosomal Intron Insertions in Genome Compacted Ray-Finned Fishes as Evident from Phylogeny of MC Receptors, Also Supported by a Few Other GPCRs
Source: PLoS One. 2011 Aug 5;6(8):e22046. doi: 10.1371/journal.pone.0022046 (PMC3151243; doi:10.1371/journal.pone.0022046)
Supplement: Table S3 — List of selected GPCRs used in this study, other than MC receptors. Codon usage in the DRY motif is shown and codon usage of the R residue is marked by red color. Proto-splice site forming codons are indicated by bold letters. The presence and the absence of intron is marked by blue background and grey background for R residue respectively. (DOC) [file pone.0022046.s013.doc]

## Table S3.

| **Gene** | **Ensembl Accession id** | **Genomic location** | **DRY intron** |
| --- | --- | --- | --- |
| P2Y2-Human | ENSG00000175591 | Chr11: 72,929,344-72,947,393 | H--R--C  CACAGCTAC |
| P2Y2-Takifugu | ENSTRUG00000005568 | scaffold_173: 403,633-406,537 | H--R--F  CATCGCTTC |
| P2Y2-Tetraodon | ENSTNIG00000010291 | Chr10: 9,958,574-9,959,932 | H--R--F  CATCGCTTC |
| P2Y2-Medaka | ENSORLG00000010300 | Chr13: 19,920,732-19,924,973 | H--R--F  CACCGGTTT |
| P2Y2-1-Sickleback | ENSGACG00000008233 | GroupI: 6,926,441-6,928,012 | H--R--F  CATCGCTTC |
| P2Y2-2-Sickleback | ENSGACG00000008228 | GroupI: 6,917,158-6,918,464 | H--R--F  CATCGCTTC |
| P2Y2-3-Danio | ENSDARG00000063545 | Chr13: 19,920,732-19,924,973 | H--R--F  CACAGATTC |
| P2Y2-1-Danio | ENSDARG00000063549 | Chr18: 2,099,809-2,100,834 | H--R--F  CACAGATTC |
| P2Y2-2-Danio | ENSDARG00000075370 | Chr18: 2,091,372-2,092,343 | H--R--F  CACAGATTC |
| P2Y2-ElephantShark | AAVX01198344 | N.A. | H--R--F  CATCGCTTC |
| P2Y3-Chicken | ENSGALG00000017327 | Chr1: 200,242,851-200,244,013 | Q--R--Y  CAGCGCTAC |
| P2Y3L-Takifugu | ENSTRUG00000005621 | scaffold_275: 233,270-234,925 | H--R--Y  CACAGCTAC |
| P2Y3L-Medaka | ENSORLG00000010357 | Chr18: 25,499,030-25,500,316 | H--R--Y  CACAGCTAC |
| P2Y3L-Stickleback | ENSGACG00000000730 | scaffold_47: 184,282-185,445 | H--R--Y  CACAGCTAC |
| P2Y3L-Danio | ENSDARG00000069459 | Chr7: 27,184,652-27,185,815 | H--R--Y  CACCGCTAC |
| P2Y3L-ElephantShark | AAVX01124620.1 | N.A. | H--R--Y  CATCGCTAC |
| P2Y6-Human | ENSG00000171631 | Chr11: 72,975,570-73,009,644 | Q R Y  CAGCGCTAC |
| P2Y6-Takifugu | ENSTRUG00000006443 | scaffold_173: 467,853-470,312 | Q R Y  CAGCGATAT |
| P2Y6-Tetraodon | ENSTNIG00000010288 | Chr10: 9,895,463-9,896,647 | Q R Y  CAACGCTAT |
| P2Y6-Medaka | ENSORLG00000010348 | Chr13: 20,047,465-20,049,757 | Q R Y  CAGCGCTAC |
| P2Y6-Sickleback | ENSGACG00000008198 | groupI: 6,839,106-6,841,996 | Q R Y  CAACGCTAC |
| P2Y6-Danio | ENSDARG00000042595 | Chr18: 2,711,904-2,712,887 | Q R Y  CAGCGCTAC |
| CHRM3-Human | ENSG00000133019 | Chr1: 239,549,865-240,078,750 | D--R--Y  GA**CAG**ATAC |
| CHRM3-Takifugu | ENSTRUG00000008654 | scaffold_29: 981,211-986,702 | D--R--Y  GA**CAG**GTAT |
| CHRM3-Tetraodon | ENSTNIG00000004464 | Chr2: 3,311,773-3,316,988 | F--R--Y  TT**CAG**GTAT |
| CHRM3-Medaka | ENSORLG00000018345 | ultracontig231: 553,737-555,656 | D--R--Y  GA**CAG**ATAT |
| CHRM3-Stickleback | ENSGACG00000015318 | scaffold_48: 1,058,562-1,062,976 | D--R--Y  GA**CAG**GTAT |
| CHRM3-Danio | ENSDARG00000071298 | Chr12: 48,724,380-48,725,885 | D--R- Y  GACCGCTAT |
| CHRM3-ElephantShark | AAVX01032697.1 | N.A. | D--R--Y  GACCGATAT |
