# Supplementary material for: Spliceosomal Intron Insertions in Genome Compacted Ray-Finned Fishes as Evident from Phylogeny of MC Receptors, Also Supported by a Few Other GPCRs
Source: PLoS One. 2011 Aug 5;6(8):e22046. doi: 10.1371/journal.pone.0022046 (PMC3151243; doi:10.1371/journal.pone.0022046)
Supplement: Table S5 — List of genomes used in this study. (DOC) [file pone.0022046.s015.doc]

**Table S5.**

| **Name of Genome** | **Version Assembly** | **Website** |
| --- | --- | --- |
| *Homo sapiens* | GRCh37 (Feb. 2009) | http://www.ncbi.nlm.nih.gov/projects/mapview/map_search.cgi?taxid=9606 |
| *Mus musculus* | NCBI m37 assembly (Apr. 2007) | http://www.broadinstitute.org/science/projects/mammals-models/mouse/mouse-genome-data |
| *Rattus norvegicus* | RGSC 3.4 assembly (Aug. 2006) | http://www.hgsc.bcm.tmc.edu/project-species-m-Rat.hgsc?pageLocation=Rat |
| *Monodelphis domestica* | MonDom5 assembly (Oct. 2006) | http://www.broadinstitute.org/mammals/opossum |
| *Taeniopygia guttata* | taeGut3.2.4 assembly (Aug 2008) | http://genome.wustl.edu/genomes/view/taeniopygia_guttata/ |
| Gallus gallus | WASHUC2.1 (May 2006) | http://www.ncbi.nlm.nih.gov/projects/mapview/map_search.cgi?taxid=9031 |
| *Meleagris gallopavo* | UMD2 (Nov 2009) | http://pre.ensembl.org/Meleagris_gallopavo/Info/Index |
| *Anolis carolinensis* | AnoCar1.0 assembly (Feb 2007) | http://www.broadinstitute.org/models/anole |
| ***Xenopus tropicalis*** | V.4.1 (Aug., 2005) | http://genome.jgi-psf.org/Xentr4/Xentr4.home.html |
| Fugu rubripes | V.4.0 (Oct., 2004) | http://genome.jgi-psf.org/Takru4/Takru4.home.html |
| *Tetraodon nigroviridis* | V7 (Apr. 2003) | http://www.genoscope.cns.fr/externe/tetranew/ |
| *Danio rerio* | Zv8 (Dec. 2008) | http://www.ncbi.nlm.nih.gov/projects/mapview/map_search.cgi?taxid=7955 |
| *Gasterosteus aculeatus* | BROAD S1 (Feb. 2006) | http://www.broadinstitute.org/models/stickleback |
| *Oryzias latipes* | MEDAKA1 (Oct. 2005) | http://utgenome.org/medaka/ |
| *Branchiostoma floridea* | V1 (March 2006) | http://genome.jgi-psf.org/Brafl1/Brafl1.home.html |
| *Callorhinchus milii* | Eshark 1.4X assembly | *http://esharkgenome.imcb.a-star.edu.sg/* |
| *Ciona intestinalis* | V2.0 (March 2005) | http://genome.jgi-psf.org/Cioin2/Cioin2.home.html |
| *Strongylocentrotus purpuratus* | Spur_2.1 (Sept., 2006) | http://www.hgsc.bcm.tmc.edu/projects/seaurchin/ |
| *Drosophila melanogaster* | FB2010_05 (May 2010) | http://www.fruitfly.org/ |
| *Caenorhabditis elegans* | Release WS187 (Feb. 2008) | http://www.wormbase.org/ |
