# Supplementary material for: Spliceosomal Intron Insertions in Genome Compacted Ray-Finned Fishes as Evident from Phylogeny of MC Receptors, Also Supported by a Few Other GPCRs
Source: PLoS One. 2011 Aug 5;6(8):e22046. doi: 10.1371/journal.pone.0022046 (PMC3151243; doi:10.1371/journal.pone.0022046)
Supplement: File S1 — Sequences of novel introns inserted into MC receptors of selected ray-finned fishes. Only introns mapping to core MCR domain (TM1–7) are considered. The numbering of intron positions refers to the full length amino acid numbering of human MC5R. (PDF) [file pone.0022046.s016.pdf]

Supplementary file S1: Sequences of novel introns inserted into MC receptors of selected ray-finned fishes. Only introns mapping to core MC receptor domain (TM1-TM7) are considered. The numbering of intron positions refers to the full length amino acid numbering of human MC5 receptor.

1. Introns of MC5 receptors mapping to position 41a

>MC5R-Takifugu-41a
GTACAGATAGTAATTACATTCTCTATGTACTTGAGTATCTGTAGCATGTTTATCAATCAT
CAGTTTTCTTACTTCCTGTCTCCTGGTGCTCCTCTATC**AG**

>MC5R-Tetraodon-41a
GTAAGGAAACGATGTTTGATTGCGCCTCACCCGTCGGCCTCTTTGGTAACTCTCTGTCTC
CTCGGCTGCTTCCTGTTTCCTGCCC**AG**

>MC5R-Sickleback-41a
GTATTAACACACACGGTGCTGAACGTGGAAATGTTTTTGTTTTGTTAAAGGTGAAACACT
CATAACGATTACCATGAAAATTGAAATATGAAAACATGAAATGCAACTATTTTTGTATCA
GGATTTTCTAAACTTTGTTGAAATTACAGATGGGGTATTCTGTTATGTTGTATGTGCTAA
TTTACATACTTTTACAGAATCAAAATCTGAACATATGAATAAAGACATTGTTTTCTTTTT
TTACTTTAGTCAAAGGTTAGAAAAGAAGATATGTTGGATATATTTTTGATTCACTCAATG
AATAAGAAAATATTGTCAACAGGCATAAAAAATAATAAACTTTATGATAAATTTGTCCAT
GTTTTTCAAATAAAATGGTATAAATCGGGCTATGAATAATTTATGAGGATATGGAGATTT
CTGGCAATAATTGAGAGAAAACAGCCTCACCTTGTGTTTAAGCCATGTGTGTCTTTTGTC
TCATTGTCGGTTTGTCTGTTATTTTGCGTGTTTTCTTTGTTCCCCAGGGAGAAGTTCTTC
TTGTATTTCTTGTCTTTTTTTTTACCTGTGACCCTCTGTTCCGTTGAGTTAACCATCGCG
CCGTCCCTCAGAGTTAGTTTGGGGGAAACCTGCTCCATTTCCGGCCACCCTGCCAATCTT
TGTATCTTCACTCCTCCAATTCTTCTCTCTCACACAGTGTTCTGCTCTCCTC**AG**

>MC5R-Medaka-41a
GTCTGTACCGAGGCGTGTAAGCTTGTTCCTGGCATTTCAGGTATCTTTGGAAAAACGTCA
ACCAGTGATGGGACTTTGAGTCAGGTCTCATTACAGTGATGCAACGCGTACCATCAAGCA
TGACACTTGTGTACATCTTTATTAGTTTCGCTGTCTTTGTAGCAAATGTAGCTAAGCAGA
GATGGATTTGTTCTTTCAGAAAGTTTGCATGAAACACTCTCGGGACTTGACTTGAACCAT
GAACATCTTTGATTTGTATTTTGCTGGATTCACACCGGACGCAATTTGCGCATCAAAATC
GCGTCTGCCGCCTCTCATTTGATGTAAATGTCAAATGTACTGATCAAGCCTTGACGCCCC
AGCCCCGCAGCAACCGCCAACGTTTTTCTGGACTTCACTTGTTGCCACCTCGAGGAAAAC
ATGGAGGATGTTGAGCAAGTAGCTTGGGGTCATATGTTTTGGAGAGCTCTACTGAGGCAC
CGTCAAGCACGCCGCTGAGTCTGGGTTCACCAGATCCTTCAAAGTGCTAACACACCAGCT
GGGTCGACTTGCATTTAAGTGGCCACTTTGAAAGAAAAGTCTATGCAAACGCATGCAGAC
CAGAATTTCTGGCTGTTGCGTCTGAAACGCACGTGATCGCGAGCTCTACGTACCAATTCA
ACCGGTTGAACTTTTTTACGCAGCAACGCAGAGATTTGAACAATCAGGGACTCGCATTTC
TTAGTGACATGTTAGTAAAGTCCAGACGTGTCATCTGTTGTAAACATGATCACAAAGGAG
ATAATAATCGCGGTTTGTGCCCAGCTGGATTAATATGACACAAAGATGGATATTTAAAGG
AACAGAGCTGGGAAAACAAAAAGCCTGGAGGAAAGTTTGCAGGACTTGCACTGCTTTGAC

TTTGCGCACTGAGAGTCAACCTATTGTATGTGTCGGACGAACGCTAGTAAACAGTAAAAA
CACAAAAGAAAAAGAATCCCCCCCTCCCTCAGGCTACCTGCGTGTCTGGCGGTGGTCCGG
TGTGAACACCACCGGCTGAATGTGAGTTCATGAATCATGCAGCACGTTCTTGAATGCACC
ACACACGGCCAGTCTGTATGCGCCTTCAGAGACTCTTCCGGTGCACTTCCGGTTGACAGG
CCGCTGCCGTCTGGATGGACCCTGGGTGGCGGCGGCCTTTCAAGGATGTATCCCGCCTGC
TCCCAACAGTAATCGGGACAGTCTCCAGCAACCCCGCGGCCCCAGCGGGTTAAGAAAATG
GATGGAAGTTCAGGACGAAAATCCTCGCATCGAGCACCCTTATTGAATTAATTTTATTCT
CATTTGATCTAGCTGTGAAAATGTCATGTGCGGAGTCCCTAATTGGTTGACACTCGTGTG
CTGCGCCTGAGACCATCGACCGAGACTCTACATTAACTTAACTTTAAAACTCGATGCCCC
TGAAGTGCAAATTGCGTCCAATGTGGACATACCTTTTGCTTATTCATCCATTGTTGTTTG
ATTGGTGCCTGCTTTTCTCCTCTGCAG

2. Introns of MC5 receptors mapping to position 77c

>MC5R-Takifugu-77c
GTGAACGTCTCATTAAAACTTCTCAAATTGGTTGACAGCATTTATTTGCTGTCCTCCCTT
TTCCCCCACAAATAAGAATCTTTTTTTTTTTTAAATTCTTGTGAACTTTTTCACCCAGCA
ATTATATAATAACACAGCTGTAGAGATTTGCGGGTTTTATGTTCATGTAATTGTGATTTT
TTTCTGGCAGCCTCGGCCTTGTGACTCTCAACAGGGAAAGTCAAACAAAGTAGACAGAAA
AATCGAATTAAAAACCATTTTGGCAGCTTAATGTTGGCGTTAATGTCAGACAGTTGTGAC
TGTAGTTGCCTGTGGGCCTCATGACTGGACGTGTTTTAAATTGTCGTGTACGTGGTGCCT
TCAGGGACAGAGGGGACTTTGATTTTCAGGGTATTTGATGGGTTCTTTGCTAAAAAAAAA
AAAGGGTGGACAGTGCAACAATCTTAGAGATCGAGGAAACATATTTGTTCAATAACATG
ATGGGGCATGAGCAGCGGAATAGCGAGCATAGGCAGCTAGCGAAGCACACCCAGAAAGAA
CCTGTAAATCAAAAGGCTGAGCTCTGATCCAGGAAGAGACACCGGTTAAAATAAGGTGAC
AACACAGGAAGCTGGAGGACACGAACAATGACAGATCTAAAAGGAAGCTTGCTGGTTTGC
TCGTTGC**AG**

>MC5R-Tetraodon-77c
GTGAATCTTTGAACATCTATGTAGAAACAAACATAAAGGGACTCATCTAAAAAACTTCAT
GGAGCTTTTGTCTGTTGTCCTCCTTCTTCCTCCTTCTTTCCAAATATGCATCTAATTTTC
TTAATGCACGTGAACTTTTGCACCCAAGGGTTGCGTAATAGCAGAGACGTATCAATTTGT
CCGTTTTTTGTTCATGTAATTGTGATTTCGACTGGCAGATGCGACCTTGTGACTCGGCAA
CAAAGCAGAAAGAGAAAAATGGAATTAAAAACCGGTTTCATGTTGGCGATGGCTGCAGAC
TGTGTGTGTGTGTGTGTGTGTGTGTGTGTGTGTGTGTGTGTGTGTGTGTGTGTGTCTGTG
ATGTATTCTGTGTTTTACTGTAAGAAACCCAGTGTTGTCACAGGAAGCAGGCTGAACTGG
GGCCTTTAGCAGTGGGAAGGTCCTGGATTCAATGTCCTTGCACATTAAGTTGAGCGGTTT
CTGCTGCTCCATGTGACCCAGGTCGGGAACAGGCAGAAAACAGACGGACGGAGCATTTGT
AGTGATCCAGAGAGCATATTATGTAAAGAGGCCTTTAGACTTATGACATTAGTGAATCCT
TGTTTAACTTGATTTTAGTATTGATAAAGCTTAATTGAAGACAGCGGGTGCTTGATCTAG
AGGATTTTAATGCAATAATAAAAGTAATTTATTAAGGATCTGCTATTCAAATGATTGAAT
TTACTAATTATTGTACCTATTTTTCCCTGAATTTTCTGTCACGGAATAATAGGTTGACTC
AATTACAAATAGGGTGGAATAGCAGCACATGCAGCTAGCGTAGCATACCCAGAATAAAAT
AAAAAAACATAAAAAGGTGTGGAGAAATGACCTCAGACCCCAGACGAGACGCAGGTTAAT
GCAAGGTGACAGGAGGACGCTGGAGCACAGGAACATTGGTCTAAAGTGAAGCTCTCTGCT
TTGGTGCTTCC**AG**

>MC5R-Sickleback-77c
GTAAACTTATTGACTCAAAACAATTGAATAGTCAACTCTCCATGCTGAGTCTTAAAGAAA

CTGATTTCTCATGAATTCTCTCTGCCTCTTTGTTTCACTTAAAAATATAAATTTGTGTTG
CCAGAGGAAAGACATTAATTTCTGAAACTGTCATTAATCCCAAATTGAAAGTGAAGGAAT
ATAAGTACATGCCTCAGGCCTTTTTGAGACTTTTTGCATTCATGTACCCTGCTGAGCTCA
TACAGACTTATTAATTAGTTTTATATAAGATTTTTGTAACTTTGCTGAAAAGTTCAATTT
GAATATGAAATATTATCTACTGGTAGCTTCTTGTACATTATGAGTGAAATCTACAAATGA
GCTGAGTGTCTACAAAGTGGACGCGGGGCTCTGTCTTGAAAATGTTTGGAAGAGTAAATA
CCCTTTTCATTGGGAACGCATAAGACGCTGGTGCGAATTGTGACTTTATGTGGATGAGAG
CCTTATGATGTAAAGCGTTAAATCCCCTACTCAAAACCAACGCAAAGAGACTCAGCTGAC
ACGCCAGCTGCGGGTTCTGGGACAAAGCAGACATTGAGTTCCCTGTGGAGTTGGTCAACA
TTAGATAATGCTTCCAATCATGTCGTTTTTTAATGATTTCTCTGAGGCAACTAGATTGTA
TCCCTGCAAAGAGTGACAGAGAACATCATAAAAACGTGCTTTTGCTGGCACCCTCTGCCA
CAGTGAGCCTCAAAGACAGAGCAGACATTAGGGACAACGAAGCTACAGACCAGCAGAGTA
CGAGGACGACTCTCCACTCAACGGTTAGAAACAGGCCGCTGGGGGCAACGAGAAGCAAAG
AAGAGCCAGGCTGAGGTTGAGATGCATTATTCACACAGAAGTTCGTGCCTTCACTACCGT
AACTCTCTCCTGGCAGGTCTTCCTGCTATCGTCATTTGACCTCTGCAGCTTATCCAGAGA
CAAGGGATGTTTCCTCAGTGTATTCTGCTGAATTGTAATTTTGTAGTGTTAGAGGGGATA
GATTAATGTCCATAAACCTTGTGCCGAATTGTAAGGTCAGTGGTTCTGCTGTGCAATGTA
TTTGGGATTCAAGTGCAATTGATAACACAATTATGCAATTTCAGAAGTGCTTAAAAATGC
ATTTCCATCTCATCATTCATGCATTTTCAAGTTGTTGGTTTTCGCTGGGACACAAATGTG
TTGTCTTGCAGGGGGGGCAGGGACAGTGCATATGAATGTCTACGCATTCATGTGCACTGT
CCTGAGCCTGACCAATCTCTTTTGGTTGTTCC**AG**

>MC5R-Medaka-77c
GTGAGCAAGTCATGTTGTGAGGGAAACATGAAACAGTTTTACACTTTTGTATTTCAACAA
TTCATTATATTGTTGAACCTGGTTTTAAATGTGCTTTGTAAGTTTGGTTAACGTCTTATT
CTCAGTGTTTAAAACTCCAGTTTCATTCATTAATGTATAGATTAAATTTCCCCCCAAGCA
TTATCCAGTGATTCCATAAACCTCTGTTATTGCTGTTTCAAAACAGTACTTTTGCCCTGC
AGCCTAAAGGCTATTTTAATTATACTTTATCAGATCACTCACTGTAGTTATTGCCACTGT
GAACCAGTGTTACCAGGTAGAATCAGTTTTTCAGCCCAGATAACATCTAAAAGCCAACAG
ACTCCCAAAAAAAACAAAAAAAACATCCAAGACGTTAATCAGAAATTATCTAGCCTGAAA
GCGTGGACAGTGTTATGAATATTTGCATGCATAAAAACCCAATACATTTGCCAACTACAA
ATTGTGTTTCATATCTTAAATCAGCATTCTTTTTACAAACAGAGCAGGTTCATTTTGGGC
CAGGAAGAATGTCTCCCAAAAACACAAACGTGAATGATACAGACAGACTATCAAATAGAC
ACAGCAAAATACAAGCTCTCTCTGACACACATCGTCGTGTGTGCACACCCACAAAATTCA
CACACTGGTTTTACCTTTAGTTGTAAACAAAGCACTTATGCCTCTTTTTCTGGACAAAAA
TCTCAGCGACTTTAGCAAAAACGTATTCCTTATGTCCATTTTTTAACATTTGCCTACTCT
GAGCAAGTGAGGCAGGTCTTGGTGCTGCCTCACCTCGCAGCCTGTGCTTTAGTTGAACAG
GACATTAGCAGAAAACAGTGATTTGGAGTATAAAAATGATCAGAATCACACACGTTGTTA
CCCTTTTGGCATATCCCTTGTGGAACCAGCTTCTACTGATCCACACAGTTGGTTTGGCAG
AGATTTCTACGCCAGATGCCCTTCCTGACACAACCCTGTATTTTATCTGGACTAGGGACT
GGCTCATGGGGACCCAGACTTGGGCCCCCTTGTGGCTACATAGTGAGGTAGTAGCGCGAA
GGGTCTCAATTTTATTTTCCATTTTATGTTTTTACTTAGATGTTATTTATTTAATTTTCA
CATCTTTCTGTAAGGCTTTAGGCAGACGTTTTAATATATATATTATACTCTAGGCTGGAA
CTTTGGAGTTTGAGGAGGCCTCAGGACCTTTCTGACCGTGAACAGCAGTGGTGACACTAG
TAGAAAGAGACCAATTAGTCCGGCTCAAAACTGCCACAAGACACAGTACAGGGTCTCTCA
CTTGGATGTTGGTGGCTGCTCCTGCTTTGAAGTTTTGCGTAGAAGGGTTGACATGTGAGG
TCGTCCTTAACGAGAAGTGACATCCAGCTCCAACCTAGCGATGGGTCCGGCAACACCAAT
GCGCAGGCTAACGTGTTGAGCTCAGAAAGTGAAACCCTGTGTCGGTGCGTGTCTCACTTC

AGGAAAATTCACGATACAGCCACTGTGTTGGTGTGTCACAGAGGGGCCAAACAGGGAAGT
GCATCTGCCAGCAGTGTTGCCAGATTCTGCGGTTTTCCACCCAGGTGGGTGGATTTGATT
TTGACATTGCAGAGAAAATCTGCTTTGGGCAGGTTTCGCATCAATTTGGTGGGGTTAATT
TAGTTTTATTTTGAGTCTAAGCTATCTTTAAATTTGAAAAATATTTTCTTTTGAAAAATT
ACTGGATCTTCTCGGTTACATTTCTGTCACTTGGGTGCTGAATTCTCGCACTGGCACTGG
CGTGGGGGAAACCCCCCGTTTCCCCCACCCATCTGTGTTTAACGTTTTCTTTTACTGAAT
CTTTCCTAAAGGTGTTAAATGTCGTGTTTTAATTAGGTTTGTGGAATTATTTGATAGTTT
TAATAGTGTTTCTGGCTTTTTGTCCCCCCCATATATGCATAATATTAATCAATTTCAAAC
TGACTCTTATTTGAAGTTAATCATTAATTATTCTGGAGGTTAAATGAGGCATTTGTTTAG
CAGAACTGTCAACCTTAAAAGCACCTCCTTAATGCTGGGGCATTATAGAACGAGACATGG
GAACGAAGAAGCAACAAATACAATGAGAAATATTGTGTTTACACTAGTTTATTTCATCAG
TACATCAGTAGAGTTTCTTCACTTGGTTACCTGCAATGATGTTTTCGGTCCAGCAGGAGT
CAGTATTGAGTGACACAGTATCAAAACAGTTTGGGCAATGTGCCGAAACGCTTCACGAAG
CTTCATCTACTCATCACTACTCCAATCCAGAAAGCTGTGTCCGAGTCCAGAGAAGGAGGG
CCAATAAAAAAGACTAAACAGGGTACAGGTATTTCATGTTTGTCTGTTGTTCATTTACTG
TTTGGATCTGACTTAAGCCACATTGAAAAGGCACAAGGTCAGGCAGGCAGTAGCTCTGTC
TTAGTGTAGTGGGGGCTCTTGAACTCATGTTTGTTCATCTGACAGCAAAAATAGCAGATG
TAATATTATTTGAATATGTCTAAATATTGGACTTTCAACGAAAACCTAAAGTAAAAATTA
AGGATGACTGTGCTGGACCAACACATCTCAACTGTTTCATCTCAAAGTTTCAGAAAATGA
CGAAGTGGAGAAATGGATTCATGAATCAGGTTTGAGGGACACTTATGAACTCAAGGTTCA
ATGAAGAATCAGTTGGAACAGTAATTGTGTGTCTGCATGCGTACTGGTCTTGTAGCAATT
ATTTATCTTTAGGCTATTTTTCCTTTTTTGAGGAGGAAATTCTATTAACATCTCTAGTTC
CACACCGAATAAAGATGACGATCCCTTGAGAAATTAATAAAAACAAGTATTTGGATATAT
TTTGTTGCTTTTTACATACTGTAGTTTATTTGCTGTTTGTTTGTTACTTTTGTCACTTAT
CTGATGTTTTTTCTACCCTCGTTGCACAATGTTTCCTTAAAGCAACAAATATATATCTGG
TCCAAACCTATCCGTTCTATCACACAATCAAATCCTGGAATGACCACATTCAGACTAGGG
TGTAACCATGTCTCCCACACATGCGATATTTTCAGAAAAATTCTTATCTGCACACACTTT
ATAAATTACAACTTTAACCACATCCCTTCTCTCAGTCACTGATATCGACACCTTTTTTCC
ACATATTCCTGTGATTTTCTTGGCCTTAGAAAGTTTTTCAGTATTTGAACAAAATAAAAC
ACTGGGTAAATTTTCAAAATAATTGAACCACAACCAGTTTTTAACTTCCTCACTACAGGT
CTGACACTACCGTAAATTAATTTGTTAATATTGTCTGAGTAGATTTGCCTTTTTAAAATT
CTTTTTGATTTAGTTTCACAATCTTTTGTATTGAAATCTTGCCAATAAAAACAAAAATCT
GCGAGGACAAGATGAAAAAGAGAGTTTATCTGGCTGTGACTCCCCATTTTGAGCAAGAGT
CACATTAGGTGTAACTCAGGAGCTGGAGGTGAAGGAGCCAAAATGCAGGAACCTGGGCTT
GGAGGTAGAAACGTAAACATAAAAGAAAAACTTTAAACAGGACAAAAACTGTGGAGAAAC
AGTGGATGACCTGACAATGTAGAACTGAAAACCACACACTTCGAGTCACTGAGGACAATG
AACAGAAATGAAGACAGCTGTGGATGATTTGCATGAACATAGAGGGACTAACCGGGGACA
GAACACCACTGAAAAACTGAACTAAACTGCAGAATCCTAGAACATTATGATAACGTTACA
TATAAAAGATTTCACCATTCTGGGGATGAAAATTGTAGGAAATCTGTGTTTAAAATATTA
CCATTTGTATAAAACTTGATTCATGTTGATTATGCCTGAAGCACATGTACCAACATAAAT
AGCTTCCAGTGTTTTATCGCTTTAATGTCTTCCAAGTCTATGATAAAATGAACAAACTTA
TGTTGACATTTGTTTATATAAAAAAAGACAATTGTTTCACTTTCACCTACACAGGACACA
TTGCTGTGTAGTTATTTATTAGTGCTTTATTTTTATACTTTACTTTTCATATTGTTTAAC
TCTTGTGCTATCTTAGATGACCCCACCCTTACATGTTACATGTTCTTCCTACCATGACAA
AGGTGGATAAAGGTGGAAAGATTTCCTGTAATCCATGGACACCAGTGAAGATCACAAATC
ATTGAAGAAAAAAGGTTCAGAGCACTGTCTAGTGGGTCTAGATGACCCAACTCCCAATGT
TAAAGTGCCTAGGATAGCACAAGGGTTACAGGAAAAAAAAGTGCTTTTGCAGCCCTTTTC
CGACTTCTGAAATCATTTGCACGCCCCTGAGCATCAGGTTAGGAGAAACGGATAACAGAG
AGCACACTGCTTTAGAAAATTGAGCACAACAGTCGCTTTCACATCAAAGAAGGGTTTGTT

TGCTTTCTTGAACATCTTTTGCATTAAAAAAACATTCATGAAGCTTTCTGGCTGTTGC**AG**

3. **Introns of MC5 receptors mapping to position 140c**

>MC5R-Takifugu-140c
GTAAGAACTTCCCGTTGGCCTCAGATGTAGACCTCTGATGTGGATTCCATTAAAGTTTAA
TTGAATTCAACCTTTTAGTGAATGTCACGGACATCTTGTGTTGCTTTGCTCATAAATGTA
AAAGGAATGTCTTTGGTTACGCCAATTGCACAGAAACAGTAAATTTAATTACATTTTAAT
GCTGCTTTATATAATTTACATTACTTGTGTCTTGAAATAGCTGGTTGTTTGTTGCAGCCC
AATTAAAAGGAACAAAAAGTCCATTTTCCAGAATATTCTGGTTGAATAAGAGTCGCAACG
TGGTATGAGCTGTAGTGTTCTGATTAAAGAGCACGAAGGAACAGATTCCCAGTTGATAGC
GGTTTATTGACTCGTGGTCTAAGATGCCTCCCACAAATTAATCAGAGAAAATCAGTTATG
AAATGAGTTCAGGAAAATCAACCTTAAATACTCATCACTCGATAGATCTAAATAGATCCA
ACATTTAACGTGTCCAGTCAACATGATTCCGAGTAATTCTGTCAGAATAGTAACGAAACA
TGAGATTTTCTCAGAACTATTAAGAAATTAAGAGCTATTAACAAATTAAGCGCCAACCTT
AATGTCAAACTCACCTTCTGTTATTCACACACACCAAAGCATTAATGAATCCAGGGAAGA
ACGAAGGGCTCCTCCCTCTGTAGACCACTGAACTAAGCAAAAACAAAACTGAACTAAACT
CATTTCCAGGTGCTGTGTATTAATTCCTCCGCACATCTGTGGTGCGTTCAAGACACACAT
GCATGGAAACAATCCCAATTAACTAAACACTATGCCACGTGGGCTTTGGAACATCCCACC
TAAAATCAACGCATAAAACACGTTTCCACCATGTCAGCACGTTCCAGTTTAATCCCAGTT
CAAGGCCGCCTGCTGCTGATCCAGATCTGAAACAAGGATCCTGATCTTTGTCATGGCCCA
AAATAACCCAATCCTTGGGTCGCTTCCAAACTCCTAACTCCAAAAATCTTGGGTTTGTGA
TAAACGATCAACAAAAACAAGCGCAGGGTCTGGTTGATTCCAGCAGTTATATCGATTTGA
GTAGACAAAGCGCAATCAAAAGTTGTCCTGGAGCCTGACCCCCGTCTTCCCGTCTGC**AG**

>MC5R-Tetraodon-140c
GTGAGAGCGCCGAAGCTCCTCAGTCTGAGCTGCTGATAAAAATGTCTGCTCGTTACCTTG
AAAGTTCTTCTGGCTGCCGCGAGAGACTGACGCCCATCCTACCGTCGGC**AG**

>MC5R-Sickleback-140c
GTAATGATGGAGGAAGGCGGCGTTGTATGAATAAATAAGCAGCATTGAGTCGTAACAGAG
ACTAGGGTGTATCCCAAAAACTTGGAGAGTTTGAGACCAAGTCAATATCAAGACCAGAGG
TCTCAAATTGTGCGTACTGTATATTTACGTACGTAGTGTTCCATGAGACAACAATCTTGC
ATTTGATGCATGGGGATATTTATTTGGTGTGCAACAGCAAACACATGCAAATAAAACTCA
AAGGAGCTTAAATCAACTTAATCATCTTCATTCAACACTTCTTTTTGACTCTACCTCTAC
AG

> MC5R-Medaka-140c

GTAAAGAACGGGAACATTTAATGATTTGATTACATCTAAATGGCATCTAATATCGAGTCATTACTTAT
CAGTTGTAAAACAGAATCTCATTCAAACGGAAATGCAATCACCAGGGAGACAACTGGCCTTAGAGCTT
TGTTAGAAATACAACCTTGATTAAACGTTGTGGTTTTGAAAAATATTAAGGGCTTCAGGGGTCTCTAAT
ATAGCTAATGTGTATGTGCAGGGGTGATGGGCAAGCTTCTGAAATGGTGGAGAAAACGAGACAAACA
AAGGAACAGCAGTAGGTCTGATAGACAGCAGCAGAACTAAAACTAAATGTGGATCCAACGGAATACT
CTTTCATCATATTGAGACAGAAATAAAGTTGCACACCGTTAAAATCCCTTTTTATGGAAGTTTATCTCC
CATAGTTTAAACTATAAAAATTCAATGGTTAACGGTTTTTGCCTCACAGCAAGAATGCCCCTGGCTCAA
GTCCTGCCTGGGGGACCTGAAACAGAACATCAATGGGGGACCTTTCTGTGTACATGGATGGGTTTTCT

CCGGAGACTGCGACTTCCTCCCACTGTCCAAAAAACGCTTCATTGGTTAGCTGGTTACTCTAAATTGTC
CCTAAGAGTGGATGTGAGTATGTATGGGTGTCTGATTTGTGGCCCTGGGACAGACTGGCGACCTGTCC
AGGATGTCCCATGCCTTCGCCCCAAAGAGTCCGGGGATAGCCCCGGGATAGGCTCCAGCAGCCCTGTG
ACCCCGAAAGGGAACAAACGGATTAAAAGATGAATGAATGAACGAATAATACTTCAATTTGCAATTT
AATCATGCAAATTTTAATGGCATTTTGCAATTTATCATTATAAATTACATTCAATCTCAAATTTTTTAAA
TTAAAGCAAAATCAATCACATTGTTGTGTTATTTCTTTAATTTACTGATTAACCCGCTTTTTCTTTCCTA
AAAATTTAGATTGGAATATGTCAGTTTCTCATCAAGTGTTTACCCACTTGCCTGGATTTTGCAAGTTCT
ACTCCCACTTTCTTTTGATAGACCATTTTAAAATATTAAAAAATACTCATCTAACAAATGTGATAATCG
GACACTGGGGAGTAGAGTAGAGAATGTACCACCCTATTAGATCGTATATTACCTACTAAGTTCATGTC
TGCAACAGATTTTTGTTCAGCCCTCAGAAGTTATTATTGGATCATTTCTTTATGGGTATTATGTCCCTTA
AAAGCTCCTGTAGGTATATGTTGTCACTTGTATGAATATGCATGGATTGGTCCATCTAGTTCTGCTGCA
TCAACAAGGGAGTATCACAATCGCAGTGAAGTCCTTAAACTGCCTCTTCACCGACCATCCAATGCGCA
GTACAGATACAGTGAGGACTTTTTAAGACCAAATGTTTGGTGCATGGGCTGATCCAGTGGGACATAAC
TGTAATTAGCAAGCCCAGCACACCAGGAGCAGCAGCTCTTGACAGCTTGCCTCCCAATTACCAGATGC
AGCAAAGTGACTGGCCCACCCTGCCACACAGAGAAAAGAGTTTTCATGCAATGGGGGCTAAGAAAGG
AGGCCACCAGTGCTTTTGAATAATCTGCACATAAGAAGCAACTCAGTGTTCGTTAGCCAGCCTAAAAA
AAGACAGCATCTGCCTTTAATCAAAACAGTCACTTTCAACACTGCAGAATGAAGCCGAGTTGGAAATA
CAACTCAGTTAAGAAGAAAGTTAATGCTGGAACGGAATTTGACCTTTTCAACACATAGATCATTGGCA
AAACCTCACAAACCGTCATGTAGAGAACAAATACCACACCTCCCATTTAAAAAAGTAAAATGTCAGAT
GAAGCAGCTGATAAAGCAGAAGATGGACCTTTCTGCTGGACCATGAACTGGTCATTGTTTTTTTTCACT
GAGATGCAGTTCGCCCTTTACTGAAGTTTCTTTTCATTTTTACCAAC**AG**

4. **Introns of MC2 receptors mapping to position 140c**

>MC2R-Takifugu-140c
GTTTGCATGATAGTCTTATTTATTCATTTTTACACACGGGTGTTTTGCCGTGTTGTTTTGTGCAACTCTC
AGAGGAGACACCATAAACCTCTTACCGGACGGATTTGTTTTCACTTTC**AG**

>MC2R-Tetraodon-140c
GTATGTTTGCCAGCCTTGTTATTTCTACAGACAGGCATGCCGCCATGTTGGTTTGTGTAACTCACAAAG
AACTTGTCGTTGTCCTTGTACACGGACGTATTTATTTCCCGTTC**AG**

>MC2R-Sickleback-140c
GTACGTTGGCAGTACAGACATGATTTAAATGAATATTTGGAGTGTTTTTAAGCTATTGCCGTGCCGTT
AAAGGAAGGATTGGATTTAAATCTGATGGGGTGAATGTTTTCCCCTCTAGCAGAGTAATCCACCAGAG
TCCCAGTATCAGACGAATCTCGAAGCTATTTGCTTCCATTTAAAATAAATGGAATAAATGTGAAGGAG
GCTGAACCAGGGTGGGTTTACTGAAGGCTTTAAAAGAAAGTTAAAGTATTTATTTGATCTGTTCACTGC
TGAGTTGGCTGTTTTGAACTGTCTCTGGTATGTGGACCCTGTCACCAATTATTCTCTGCTCTTTAAATTC
AAAGTCTCAACTACGACTCTGAAAATTTTTGAAACGTCTGAGCTTTTTTCTGGGCTCATAGATCCACAG
CTCATTAAAAACCCACTGCTCTTCCTATTACTGCTACGGTTACACTTTTTCACACCACGATTCAGCCCA
GTTCAGCATAGTATCACATCGTGAAGTTACATTTATTTTAGATAAAAATTCTGCCAACTTACATTTGTA
GCTCACATTACATTACATGTCATTTAGCTGACGCTTTTATCCAAAGTGCATTTACCAAATATGACATTG
CTGTGATGCTCACAGTGATACGGTATTAGTTATGAATAAAAATTCAACAATTCTAAAAAAATATGTTG
TAGTAATTTAATTAATTTTTCTGTTTCCTACAGTTATTATTGCTTACCAAACACAACAAAGACACTTTTG
TTGTGGTATTTGTTAAAAATGTTTGTGGGTACATTGCTGTTCAATGGGACTAATGACCGGGGGGGAAA
CGACGGCATCGCCGGGTTTCCCAAATAAAGAGTTACAATCGAGAGCAGTTTGTTCTCTCACTGTATTCA
AAAACATGACAGTAGTTGTTACAAATAACTTTGGTTCTGTCAACTCGCCGTGTGGAATGTATTTCAAAT
GAAAATGTCCACAAAAGAGTTTGCCACCTTTCTCCATTTCTCTGTTTCGGTTCATACAAGCAGTTTAAT
GCCGTCAAGGGTTTGGTACCTTTTGTACGACCTGCAGGCCTGGATGTGTAGCTCCATACAGCTGAGGG
GAATTGCAGAGTCAGGTGATCTCCGTGGGTTTTATTCATTGATTACCTGAGCAAGTTCAAGTGCAACTG

AAATTAAAATTATTATATATATTTTAACTATTGATTTCCATTTAATTATATTTTTGTAGTTTCTACCTGC
CGCCCCATAGTTTATCATGAATTCAATCGAACACCGCTCAGTTATCCGTGTAGATGGAGACCCTATTTT
GATACAGCAAATCAAATCTTGCATCACGTGTTAAATCAGAGCTTAGCTGGCTTTCATAAAAATGCTCA
CATGACAGAAACGTTTTCTTTTCCACCTCC**AG**

>MC2R-Medaka-140c

GTACGTAAACAGGAAATACTTCAGAACATTTAGGATGCTCTTTTGCAAACCACTGATTAAGCAATGCA
AATGTCCAGACCTTTATCATTAACGCTCACATTTCATTATGGAACTTTAGTCTGCTTCAGACATCTTAA
ATCAGGTTTTTTCCAGATGACTGCAGAACTACTGCCAAAGTGATCAGAGAGACTGGGAGGAAGGGACT
TGATGTATCATATAGAATGAGGAAAGAAGATCAAGGGGACTTGGTGGTGGAATGAAGAAGCTCAGGA
GTGCGTTAAAAGGAAAAGGTTCGCTGTGAAAACATGACGCACTGAGAGGACAGAAGGAGGAGTAGAT
ATAGCACAAGCTGAAAGTGGAGGTGGCAAAGGCCAAACAGAGGGTGTATGAGGGCTTGTATGACATG
TTGGAGGGAAGGAGAAGAGCAAGTGATGGATCTGAGATGGATCTGAACAGGTGATGCAGAGAAACA
GAGGGAAGGATAAGCCGCAGGTTAGCGTGACCAAGGCCTCTGATGAAAATATGTTGACAGCTTTGGA
AGGAGAACTTTGAAGACATTCTTAAAATGTTAGAGGGCACAAGGTTGAGGCAGGCAGTAGTACATTTT
ACCAAGGATTCATCCTTTAAACTGAAGTCAGGAAGTCATTGAAGATGATGAAGACAGCCAACAACAA
TCAAAGGTCAATAAAGATGCAGAACACAGTGCACCCTTTGAAAAAAGCATGCAAAGTTGCACCGGAA
GAAATTTACAAAACAGCAAACTGAAAGATGAACCATGATATAGCGAAGACACATTGTAGAGGAAGGT
AGACTGAGGTCAGAAGTGAACATTTGTGAGCCACAATGTTTTCATGAACTGTAAAAGAACCACAGATG
CAATATTTGCTTTGAGAATGCCTCTGGAGAAGAACAAGGTCAGAGGGATCTGTACTGTGCCTTTGTAG
ATCAAGAGAAGGTCTATGACAGAGTGTGACAGGGAGCTGTGGTTTGGATGAGGAAGTCCATGTATAG
AAATACGTTGGAGTTGTGCAGGACACGTATGAGGGCTGGAAGACAGTGGTGAGATGGGTTGTAGGTG
TGAGAGAGGAGTTGAAGGTGGAGGTGGGACTACACTGAGGTCAACGTTGAACCCTGTCTTGTTTGCTG
TGGTGATGGACAGACTGACAGACGAGGTTAGACTGGAATTTCTGTAGACTATGATACTGAACAGTCCA
GAAAGTGTAGTAAACAGGTGAAAAGGTGTCTGAAGCTAGTTGGAACGGGTGGAGGAATGTTTCAGGT
GTGATGTGTGACTAAACAGTGTTAGAATAATGAAAGGTAAAGGTGTAGAAGACTATGGTGAGAGCAG
CCATGTTGCTCTGATGCAGAGCTGGAGGCAGCAGAGATGAAGATGTTGAGGTTCTCTTTGGGAGAGAC
CGGGATGGATCAGGAATGAATCCATCAGAAATCAGATCATACTAAATGTTCTGGAGATCAATGCAGA
GAAGCAGATGGAGATGTTTGAACACGTGATGATGTTGGTGAAAGGATGCTGAGGTTGGAGCTACCAG
GAAAGAGAGGAAGACCAAAGAGGAGGTTCATGGATGTAGAGGAGGACATGAGGGGGTTGGTGTGAA
TGAGGAGGATGCAGAGAAAGGATTAGAGGGACATGCACGATTTACTGTGGGAACCCTAATCAGAACA
GCCAAGAGACAAAGAATAAGAAGATCAGATTTAATCATGTTTCATGACTGAAAGGTTTAAATTTTCAG
GCTAGCGCACTTCTGAAGATTTTTTTGTGACAATCTGGAGTTGTGATCTCCAAAAACTGTTGAGGGCAG
TAATAGCCCTTGAAATCATTTTCTTTCTGCCTTTC**AG**

5. **Intron of MC2 receptor mapping to position 230c (only in *Takifugu*).**

>MC2R-Takifugu-230c
GTGGGGCCATGGCATG**AG**

6. **Intron of MC2 receptor mapping to position 236a (only in sickleback).**

>MC2R-Sickleback-236a
GGCCGGCGGCGGCGGCGGCGGTGGGGCAGCG**GC**
